# Combination of betulinic acid and EGFR-TKIs exerts synergistic anti-tumor effects against wild-type EGFR NSCLC by inducing autophagy-related cell death via EGFR signaling pathway

**DOI:** 10.1186/s12931-024-02844-9

**Published:** 2024-05-20

**Authors:** Han Wang, Xiaohui Du, Wenwen Liu, Congcong Zhang, Ying Li, Jingwen Hou, Yi Yu, Guiru Li, Qi Wang

**Affiliations:** 1 The Second Hospital of Dalian Medical University, Dalian, 116023 China; 2 Guangzhou women and children’s medical center, Guangzhou Medical University, Guangzhou, 510623 China

**Keywords:** EGFR-TKIs, Betulinic acid, Wild-type EGFR, Autophagic cell death, Cell cycle arrest, Non-small cell lung cancer, Primary drug resistance, Combination therapy

## Abstract

**Background:**

Epidermal growth factor receptor (EGFR) tyrosine kinase inhibitors (TKIs) have revolutionized the treatment of lung cancer patients with mutated EGFR. However, the efficacy of EGFR-TKIs in wild-type EGFR tumors has been shown to be marginal. Methods that can sensitize EGFR-TKIs to EGFR wild-type NSCLC remain rare. Hence, we determined whether combination treatment can maximize the therapeutic efficacy of EGFR-TKIs.

**Methods:**

We established a focused drug screening system to investigate candidates for overcoming the intrinsic resistance of wild-type EGFR NSCLC to EGFR-TKIs. Molecular docking assays and western blotting were used to identify the binding mode and blocking effect of the candidate compounds. Proliferation assays, analyses of drug interactions, colony formation assays, flow cytometry and nude mice xenograft models were used to determine the effects and investigate the molecular mechanism of the combination treatment.

**Results:**

Betulinic acid (BA) is effective at targeting EGFR and synergizes with EGFR-TKIs (gefitinib and osimertinib) preferentially against wild-type EGFR. BA showed inhibitory activity due to its interaction with the ATP-binding pocket of EGFR and dramatically enhanced the suppressive effects of EGFR-TKIs by blocking EGFR and modulating the EGFR-ATK-mTOR axis. Mechanistic studies revealed that the combination strategy activated EGFR-induced autophagic cell death and that the EGFR-AKT-mTOR signaling pathway was essential for completing autophagy and cell cycle arrest. Activation of the mTOR pathway or blockade of autophagy by specific chemical agents markedly attenuated the effect of cell cycle arrest. In vivo administration of the combination treatment caused marked tumor regression in the A549 xenografts.

**Conclusions:**

BA is a potential wild-type EGFR inhibitor that plays a critical role in sensitizing EGFR-TKI activity. BA combined with an EGFR-TKI effectively suppressed the proliferation and survival of intrinsically resistant lung cancer cells via the inhibition of EGFR as well as the induction of autophagy-related cell death, indicating that BA combined with an EGFR-TKI may be a potential therapeutic strategy for overcoming the primary resistance of wild-type EGFR-positive lung cancers.

**Graphical abstract:**

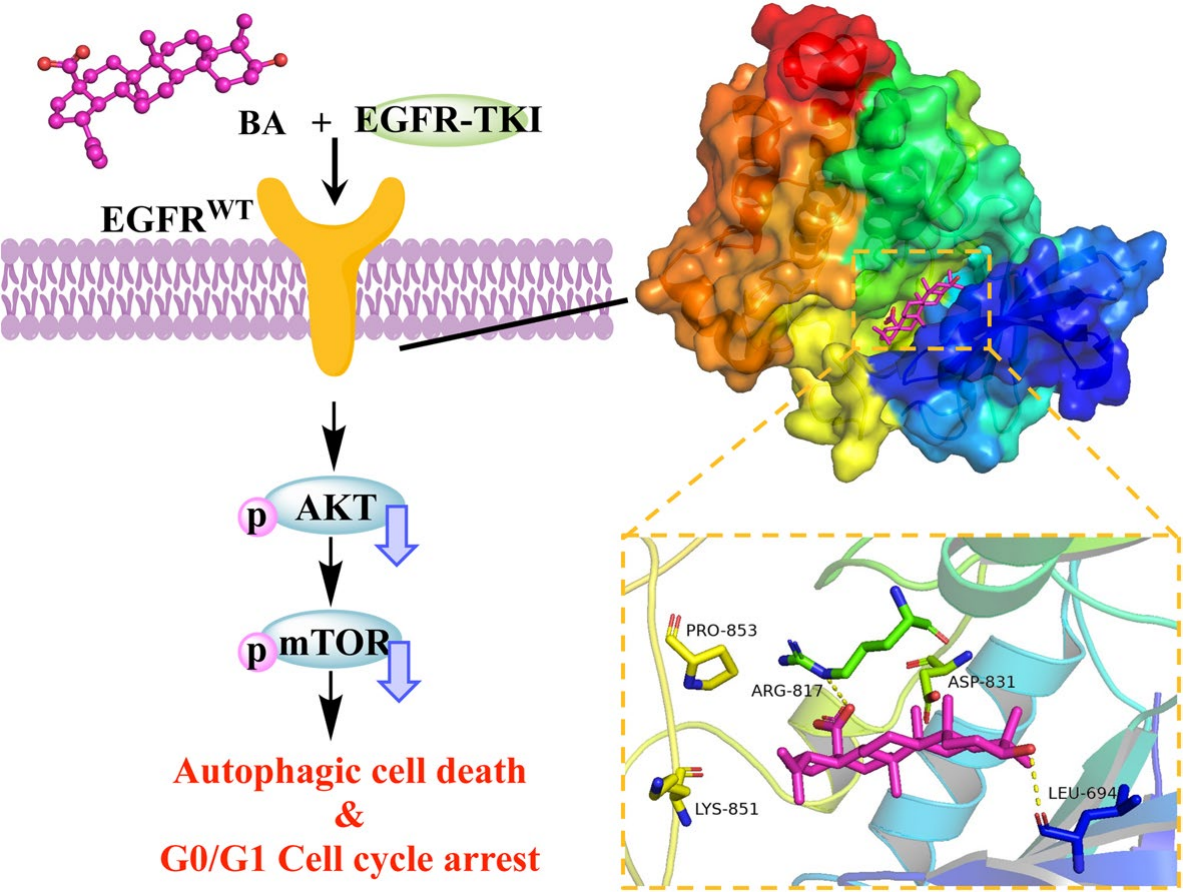

**Supplementary Information:**

The online version contains supplementary material available at 10.1186/s12931-024-02844-9.

## Highlights

1. BA is a potent wild-type EGFR inhibitor.

2. BA enhances the anticancer efficacy of erlotinib/gefitinib in NSCLC patients with wild-type EGFR.

3. The combination of BA and gefitinib/osimertinib synergistically blocks EGFR and inhibits the PI3K-AKT-mTOR signaling pathway, leading to autophagic cell death and cell cycle arrest.

4. This combination strategy targets wild-type EGFR in NSCLC and induces autophagy and tumor suppression in vivo.

BA, an EGFR inhibitor, sensitizes EGFR-TKIs’ activity, indicating that BA plus EGFR-TKIs may be a potential therapeutic strategy to overcome the primary resistance of wild-type EGFR NSCLC.

**Supplementary Information:**

The online version contains supplementary material available at 10.1186/s12931-024-02844-9.

## Introduction

Lung cancer is one of the most commonly diagnosed cancers and the leading cause of cancer-related deaths worldwide [1], and non-small cell lung cancer (NSCLC) represents the major histological subtype of the disease. In the most recent decade, the treatment of NSCLC has evolved dramatically. The discovery of multiple oncogenic driver alterations and the development of matched molecularly targeted therapies have allowed the creation of personalized targeted treatments [2]. Numerous tyrosine kinase inhibitors (TKIs) targeting epidermal growth factor receptor (EGFR) with demonstrated efficacy in patients with EGFR-mutant NSCLCs have been developed [3]. Gefitinib is a first-generation EGFR-TKI that was proven to be efficacious for treating NSCLC harboring an EGFR mutation. Osimertinib is a third-generation TKI that is effective against T790M-mutated EGFR, which is the most common mechanism of acquired resistance to gefitinib. EGFR-TKIs inhibit tumor growth by blocking the ATP-binding site in the EGFR kinase domain [4–6]. Patients with EGFR mutations are 100-fold more sensitive to EGFR-TKIs than patients with wild-type EGFR [3, 7]. Although TKIs have made significant advances in the care of patients harboring activating EGFR mutations, the indications for EGFR-TKIs in patients with wild-type EGFR (wt-EGFR) tumors are debated [7, 8]. Some tumors with wt-EGFR exhibit intrinsic resistance and fail to respond to initial treatment. Therefore, the underlying mechanism of primary EGFR-TKI resistance needs to be investigated in depth. The development of new EGFR-TKI sensitizers and combination strategies to overcome innate resistance to EGFR-TKIs is urgently needed for NSCLC patients with wt-EGFR.

Many efforts have been made to elucidate the underlying molecular mechanisms of EGFR-TKI resistance in NSCLCs expressing wild-type EGFR genes [9–11]. However, the prognosis of patients with wt-EGFR has not improved significantly, and platinum-based chemotherapy remains the backbone therapy for wt-EGFR NSCLC patients who cannot receive the corresponding targeted drugs. Although different strategies have been explored to overcome the primary resistance of wt-EGFR to TKIs [12–14], there is still a need to develop other strategies dedicated to different sensitization mechanisms, which may improve the understanding of resistance and provide therapeutic regimens that could maximize the therapeutic efficacy of EGFR-TKIs in wt-EGFR lung cancer. Recently, accumulating evidence has shown that autophagy limits tumorigenesis by promoting the degradation of accumulated damaged proteins and organelles. Under certain circumstances, the induction of autophagy not only promotes autophagic cell death but also enhances the sensitivity of various types of cancer to antitumor therapeutics. Moreover, not only has EGFR been reported to modulate autophagy in NSCLC [15] and other mammalian cancer cell lines [16], but all its downstream signaling pathways have also been demonstrated to regulate autophagy [17]. The concomitant targeting of EGFR and autophagy might offer a window of opportunity to combat survival advantage and drug resistance in cancer treatment. However, the relationship between EGFR-TKI resistance and autophagy is still poorly understood, and TKI-modulated autophagy in lung cancer cells has been postulated to exert either cytoprotective or cytotoxic effects [15].

To discover more potent EGFR-TKI chemosensitizers, we performed drug screening and identified betulinic acid (BA) as a promising wt-EGFR inhibitor that can block the activation of EGFR in wt-EGFR NSCLC cell lines. BA, a lupine-type pentacyclic triterpenoid, has antitumor activity [18–20] without significant adverse effects [21, 22], and its mechanisms of action mainly include the induction of mitochondrial apoptosis [23–25], the regulation of specific protein transcription factors [26, 27], the inhibition of STAT3 and NF-κB signaling pathways [28–30], and the mediation of autophagy [19]. In addition, BA has emerged as a promising therapy for overcoming drug and radiation resistance [27, 31, 32]. To improve the antitumor activity of BA, a suitable delivery system has become a research direction [33–35]. Current studies have shown that BA is a powerful anticancer agent even in drug-resistant cell lines, and its multitarget behavior provides a potential advantage to BA since resistance can be overcome by modulating more than one target. Here, we explored the effect of BA on the sensitization of wt-EGFR lung cancer cells to EGFR-TKIs (gefitinib and osimertinib). Our results suggested that BA potentiated the efficacy of EGFR-TKIs against wt-EGFR NSCLC through suppressing EGFR-induced proliferation and carcinogenesis. Mechanistic study revealed that the synergy between BA and EGFR-TKIs effectively induced autophagy-related cell death and G0/G1 cell cycle arrest. In particular, the EGFR-AKT-mTOR pathway may account for the main mechanism involved in overcoming innate TKI resistance. This study provides a new perspective for the development of BA and its more potent and selective derivatives, demonstrating the possibility of overcoming the primary resistance of wt-EGFR NSCLC by inducing autophagy-related cell death.

## Materials and methods

### Cell culture and compound reagents

All human NSCLC cells (A549, H1299, PC9, H827 and H1975) were obtained from the Chinese Academy of Sciences (Shanghai, China) and cultured in RPMI 1640 supplemented with 10% fetal bovine serum (FBS) and 1% penicillin‒streptomycin (all obtained from Gibco, Invitrogen, Inc., USA), and a humidified atmosphere containing 5% CO_2_ at 37 °C was used for maintenance. BA was purchased from Aladdin (Shanghai, China), gefitinib, osimertinib, chloroquine, 3-MA, 3BDO and MHY were obtained from MCE (USA).

### Molecular docking

The structures of WT EGFR (PDB: 4HJO and 4ZAU), L858R EGFR (PDB: 2ITV) and T790M EGFR (PDB: 3W2O) were obtained from the Protein Data Bank (PDB) database. Water molecules and ligands were removed from the crystal structures, followed by the addition of only polar hydrogen. Docking was performed based on the standard precision mode of Glide with default settings. Molecular docking among ligands and proteins was carried out using Discovery Studio. The docking poses for each receptor-ligand complex were then analyzed for binding modes, and the docking results were visualized using PyMOL. The hydrogen bond length was defined as the distance between the donor and acceptor atom centers.

### Western blotting and antibodies

Cells were lysed in SDS lysis buffer, total protein was harvested, and the protein concentration was determined using BCA protein assay kit (Meilunbio). Then, 30 *µ*g of lysate was separated via 4–12% SDS‒PAGE, followed by transfer onto a PVDF membrane. After blocking with 5% milk-TBST, the PVDF membrane was washed three times and then incubated with primary antibodies at 4 °C overnight and secondary antibodies at room temperature for 1 h. For western blotting, the following antibodies were used: EGFR (1:1000, CST, USA, 4267), p-EGFR (Tyr1068) (1:1000, CST, USA, 3777), p-MET (Tyr1234/1235) (1:1000, CST, USA, 3077), p-AXL (Tyr702) (1:1000, CST, USA, 5724), p-Her2 (Tyr1221/1222) (1:1000, CST, USA, 2243), C-PARP (1:1000, CST, USA, 5625), p-AKT (Ser473) (1:1000, CST, USA, 5625), p-Erk1/2 (Thr202/Tyr204) (1:1000, CST, USA, 4370), LC3 (1:1000, CST, USA, 12,741), p62 (1:1000, CST, USA, 8025), Beclin1 (1:1000, Abcam, USA, ab207612), Cyclin D1 (1:1000, CST, USA, 2978), Cyclin B1 (1:1000, CST, USA, 12,231), CDK4 (1:1000, CST, USA, 12,790). An antibody specific to GAPDH (1:10000, Proteintech, Wuhan, China, #60004-1-lg) was used as an internal control.

### Cell viability assay

Cell Counting Kit-8 (CCK-8, MCE, USA) was used to measure cell viability. The cells were seeded in 96-well plates, cultured overnight, and treated with a dilution series of test compounds for 72 h. Then, 10 *µ*l of CCK-8 reagent was added to each well, followed by 2 h of incubation at 37 °C. The reaction was evaluated according to the absorbance at 450 nm. All experiments were performed in six duplicate wells.

### Drug interaction analysis

To quantify the interactions between BA and gefitinib/osimertinib, SynergyFinder was used to estimate the synergy scores of the proposed drug pairs. All simulations were performed assuming that the two drugs were combined in a nonfixed ratio of doses with variable concentrations of both Drug 1 and Drug 2. Synergy scores < -10 indicated that the interaction between two drugs was likely antagonistic; scores from − 10 to 10 indicated that the interaction between two drugs was likely additive; and scores > 10 indicated that the interaction between two drugs was likely synergistic. Drug interactions in each combination were analyzed by ZIP models, which evaluate the drug interaction relationships by comparing the change in the potency of the dose-response curves between individual drugs and their combinations, assuming that two non-interacting drugs were expected to incur minimal changes in their dose-response curves [36–38].

### Colony formation

Cells were seeded into a 6-well culture plate (5 × 10^2^ cells/well) and incubated with compounds for 14 days, after which the medium was replaced every 2 days. The colonies were fixed with 4% paraformaldehyde (PFA) for 30 min and washed with PBS. Colonies were stained using crystal violet solution (0.1%). The stained colonies were photographed after washing with PBS and normalized to the control.

### Apoptosis and cell cycle assays

Cells were seeded 24 h prior to the experiment to a confluency of 30–50%. Forty-eight hours after the addition of drugs, the cells were harvested, rinsed with cold PBS and double-stained with Annexin-V FITC and PI for 15 min at room temperature in the dark. The subpopulation of apoptotic cells was analyzed by flow cytometry (Agilent NovoCyte Advanteon). The percentage of annexin-positive apoptotic cells was analyzed using NoveExpress software. For cell cycle analysis, the cells were suspended in 70% ethanol, incubated at -20 °C for 2 h, and collected by centrifugation to remove the ethanol. The cells were stained with PI staining solution and analyzed by flow cytometry (Agilent NovoCyte Advanteon).

### Tumor xenograft model

All procedures related to animal care, handling, and treatment were performed according to the guidelines approved by the Institutional Animal Care and Use Committee (IACUC) of Dalian Medical University (IACUC approval no. AEE22077). All efforts were made to minimize animal suffering and the number of animals used. Five-week-old female BALB/c nude mice were acclimated to the environment for approximately one week before in vivo experiments. A total of 1 × 10^6^ A549 cells were suspended in 100 *µ*l of PBS and subcutaneously injected into the flanks of the nude mice. After the tumor size reached 100 mm^3^, the tumor-bearing mice were randomly assigned to control or treatment groups and dosed with vehicle control or test compounds, respectively. For the in vivo experiments, gefitinib (30 mg/kg/day) was given by oral gavage every day, and BA was given at a dose of 30 mg/kg every two days by intraperitoneal administration. Body weight was recorded throughout the entire experimental period. Tumor volume was measured using calipers and calculated with the formula ([width]^2^ × [length]/2).

### Histology and immunohistochemistry (IHC)

The tumors were harvested for histopathological examination. Tissues were fixed in 4% (v/v) PFA for 24 h, washed with PBS, embedded in paraffin, and sliced into 5-*µ*m thick sections. Paraffin sections were dewaxed with xylene, rehydrated by passage through decreasing concentrations of ethanol, and then stained with hematoxylin and eosin (H&E). For the immunohistochemistry (IHC) assay, the tumor sections were incubated with primary antibodies specific for Ki67 (1:2000, Proteintech, Wuhan, China, #27309-1-AP) and LC3 (1:500, Proteintech, Wuhan, China, #81004-1-RR) overnight at 4 °C, followed by incubation with secondary antibodies. The sections were visualized with DAB kit, and images of three randomly chosen fields on each slide were captured under microscope.

### Statistical analysis

All experiments were performed at least three times. The data are expressed as mean ± standard deviation (SD). The analysis was carried out using GraphPad Prism 8. Difference between two groups were analyzed by Student’s *t* test, and significance was set at *p* < 0.05. The specific details about the statistical methods used are provided in the respective figure legends.

## Results

### BA targets wt-EGFR and blocks EGFR signaling pathway

To identify candidates that could overcome the intrinsic resistance of wt-EGFR, we established a focused drug screening system. Using a structure-based approach, we detected a series of natural compounds that target wt-EGFR with selectivity over mutant forms of EGFR. Molecular docking assays were performed to identify the binding mode mediating the interaction between compounds and EGFR proteins. Docking studies were performed against the ATP-binding sites of EGFR tyrosine kinases with different gene backgrounds, including wild-type (EGFR^WT^ PDB: 4HJO and 4ZAU) and mutant (EGFR^L858R^ PDB: 2ITV, and EGFR^T790M^ PDB: 3W2O) EGFR. Among the docked compounds, BA was identified as a distinct molecule with good binding affinity for EGFR (energy score of -6.45 to -8.86 kcal/mol).

**Fig. 1:**
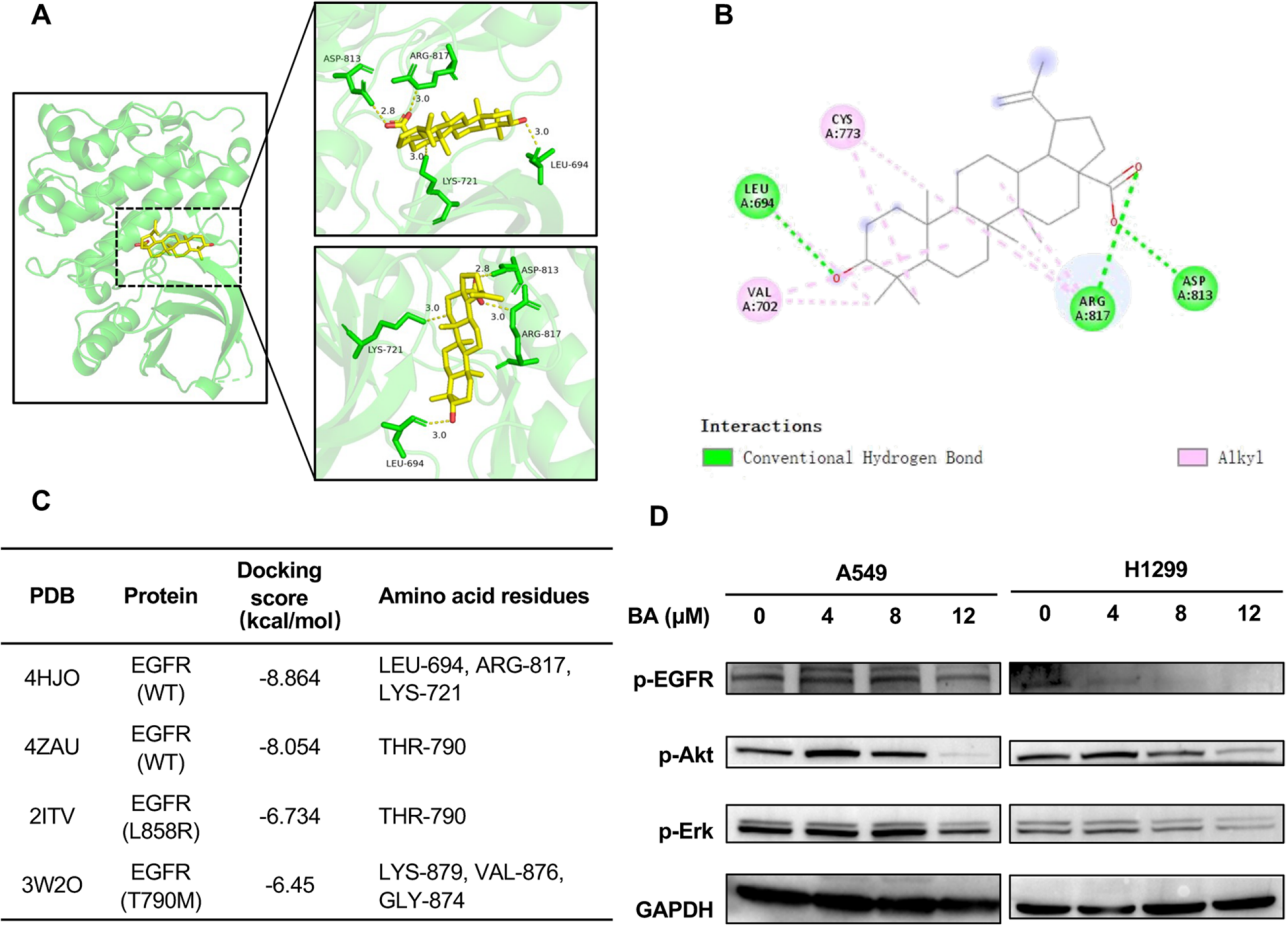
BA bound and inhibited the activation of wt-EGFR in lung cancer cells. **A** The BA-binding mode for wt-EGFR. BA is shown as sticks (C, yellow; O, red), and EGFR is depicted in cartoon representation with key residues indicated as green sticks and labeled. Hydrogen bonds are shown as yellow lines. **B** 2D diagram representation of BA in the wt-EGFR binding site using Discovery Studio Visualizer. **C** Docking study of BA with EGFR^WT^, EGFR^L858R^, and EGFR^T790M^. **D** BA specifically inhibited the phosphorylation of tyrosine residue 1068 on wt-EGFR and its downstream signaling in the A549 and H1299 cell lines

EGFR includes transmembrane domain, extracellular and intracellular kinase domains. The tyrosine kinase domain of EGFR consists of essential regulatory elements, including the αC helix (amino acids 729–744) and activation loop (amino acids 831–852) [39]. These elements are important for allosteric regulation and conformational changes and for controlling EGFR activation. Figure 1a and b show the 3D and 2D structures of ATP binding sites of wt-EGFR domain kinase (EGFR^WT^ PDB:4HJO), which was identified to have the highest binding affinity of -8.86 kcal/mol with BA. The carboxyl group of BA interacts with the active site of the receptor by forming two hydrogen bonds with the edge of the activation loop, Arg817 and Asp813 [40–42], and the 3-OH group couples with hydrophobic region II, generating a hydrogen bond with the Leu694 residue. Moreover, the BA skeleton generated one hydrogen bond with Lys721 and hydrophobic interactions with the amino acids Val702 and Cys773. The 4ZAU crystallographic structure contains the AZD9291 transferase inhibitor and the wt-EGFR domain kinase. The docking results suggested that BA could penetrate deeply into the ATP-binding pocket and form a hydrogen bond between the gatekeeper Thr790 and the carboxyl group of BA, with a binding energy of -8.05 kcal/mol (Fig. S1). In the L858R mutated EGFR pocket, the hydrogen bonds between the amino acids Leu777 and Leu778 and the 3-OH group of BA were also critical, which hindered the free movement of the Met790 side chain (Fig. S2). BA interacted with the active site of T790M-mutated EGFR via four hydrogen bonds with Gly874, Val876, Ile878 and Lys879, with a binding coefficient of -6.45 kcal/mol (Fig. S3).

These findings indicated that BA could target the ATP-binding domain of EGFR. To better understand the underlying mechanisms, we determined whether BA could affect the EGFR signaling pathway. The activity and selectivity of BA against cancer cells expressing EGFR were assessed in a panel of cell lines, including NSCLC cell lines harboring either the EGFR^L858/T790M^ double mutation (H1975 cells) or EGFR^exon19del^ (PC-9 and H827 cells) and two cell lines expressing wt-EGFR (A549 and H1299). BA dose-dependently decreased EGFR phosphorylation at the Y1068 site (Fig. 1d) and induced a more spindle or elongated cell shape in A549 and H1299 cells. Additionally, BA substantially inhibited the phosphorylation of Akt (Ser473) and Erk1/2 (Thr202/Tyr204) (Fig. 1d), which are well-known downstream targets of EGFR. However, it showed moderate or weak inhibitory activity on EGFR mutations (H1975, PC9 and H827 cell lines) (Fig. S4), demonstrating the selectivity of BA at the cellular level. Overall, these data showed that BA, as an EGFR blocker, could bind to the ATP-binding pocket of wt-EGFR and inactivate EGFR downstream signaling.

### BA potentiates the inhibitory effect of EGFR-TKIs on wt-EGFR cells

**Fig. 2:**
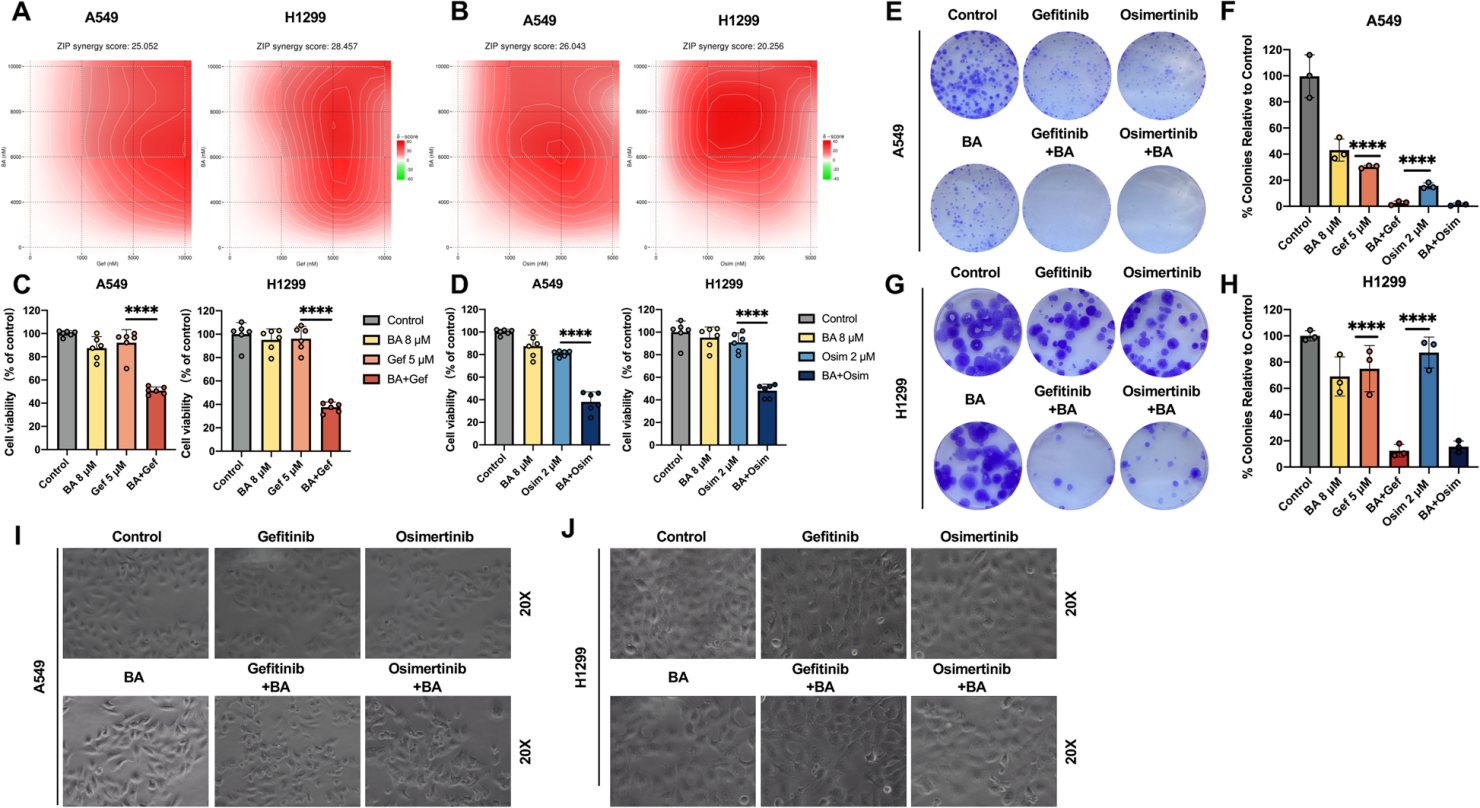
BA at a low dose synergistically enhanced the cytotoxicity of EGFR-TKIs against NSCLC cells expressing wt-EGFR. **A** and **B** Heatmaps of drug combination responses. BA combined with gefitinib (**A**) or osimertinib (**B**) acted synergistically on A549 and H1299 cells. BA and gefitinib/osimertinib at the indicated concentrations were used to treat cells for 48 h, and cell viability was assessed by a CCK-8 assay. ZIP synergy scores were calculated using SynergyFinder software. Scores > 10 were considered to indicate strong synergistic effects. The gradation of the red regions indicates the intensity of synergism. Mean ± SD, *n* = 3. **C** and **D** The combination of BA with gefitinib (**C**) or osimertinib (**D**) synergistically inhibited the growth of A549 and H1299 cells. The data (mean ± SD, *n* = 6) were analyzed using Student’s *t* test. **** *p <* 0.0001. **E** and **F** Colony formation assays showed that the combination of BA with gefitinib (**E**) or osimertinib (**F**) decreased the number of colonies. The data (mean ± SD, *n* = 3) were analyzed using Student’s *t* test. **** *p <* 0.0001. **G** and **H** Photographs showing morphological changes in A549 (**G**) and H1299 (**H**) cells after the indicated treatment for 24 h

EGFR-TKI, the current first-line therapy for NSCLC harboring EGFR mutations, is encountering the clinical challenge of primary resistance in patients with wt-EGFR lung cancer. The suppression of wt-EGFR signaling induced by BA in NSCLC may benefit EGFR-TKI therapy. To examine whether BA could potentiate EGFR-TKIs in lung cancer cells expressing wild-type EGFR gene, we evaluated the inhibitory effect of combination treatment with BA and EGFR-TKIs (gefitinib and osimertinib). Cells were treated with increasing concentrations of TKIs for 48 h in the absence or presence of different doses of BA, and survival inhibition was measured by a CCK-8 assay. Compared with A549 and H1299 cells treated with BA or EGFR-TKI alone, A549 and H1299 cells treated with the combination strategy displayed significantly decreased viability (Fig. S5 and S6). According to the concentration gradient and the corresponding inhibition indices, the drug-ZIP synergy scores were calculated with the online SynergyFinder software (Fig. 2a and b). BA combined with EGFR-TKIs had a strong synergistic effect on inhibiting tumor proliferation (ZIP synergy score > 10), and the average proportions of the antitumor response attributable to BA combined with gefitinib/osimertinib were 25.052/26.043 in A549 cells and 28.457/20.256 in H1299 cells, indicating that these combinations had synergistic inhibitory effects. Based on these results, the lowest concentration of BA, 6 *µ*M, had the greatest synergistic effect on both the A549 and H1299 cell lines. To further observe the synergistic effect and confirm the best combination concentration, we detected cell viability (Fig. 2c and d) and colony formation (Fig. 2e and f) under different treatment schedules. The most effective concentrations of BA (8 *µ*M), gefitinib (5 µM), and osimertinib (2 µM) were selected as the best combined concentrations for synergistic antiproliferative effects because they had synergistic effects. The results indicated that BA at a concentration well below the IC_50_ (higher than 20 *µ*M for both A549 and H1299 cells) could still synergistically potentiate the antitumor activity of gefitinib or osimertinib against wt-EGFR NSCLC cells. Based on these data, we speculated that the wt-EGFR inhibitor BA might be a potential sensitizer mediating resistance to EGFR-TKIs in lung cancer cells expressing wt-EGFR and that treatment with BA plus EGFR-TKI is a promising combination therapy for intractable NSCLC harboring wt-EGFR.

### Combination treatment induces autophagy-related cell death via EGFR-AKT-mTOR signaling pathway in NSCLC

**Fig. 3:**
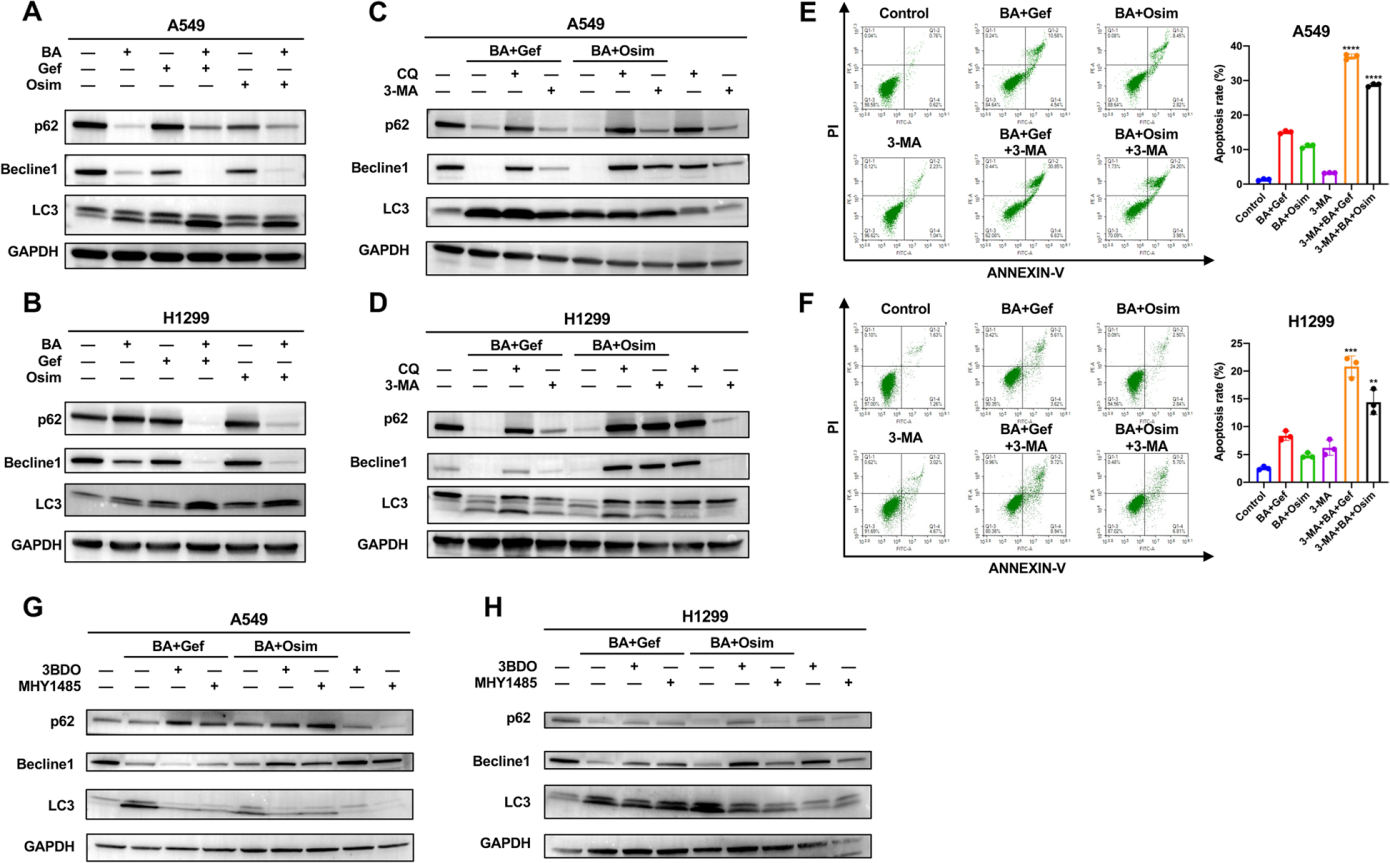
Combination treatment induces autophagy and autophagy-related cell death via AKT-mTOR pathway. **A** and **B** A549 and H1299 cells were treated with BA, gefitinib and osimertinib alone or in combination for 48 h. Whole-cell lysates were extracted, and the LC3, p62 and Beclin1 proteins were detected by immunoblotting. GAPDH was used as internal control. **C** and **D** A549 and H1299 cells were treated with the indicated supplements for 48 h in the absence or presence of chloroquine (10 *µ*M) or 3-MA (5 mM). LC3, p62 and Beclin1 proteins were detected by immunoblotting. GAPDH was used as an internal control. **E** and **F** A549 and H1299 cells were treated with the indicated supplements for 48 h in the absence or presence of 3-MA (5 mM), and cell apoptosis was analyzed by flow cytometry using Annexin V-FITC and PI cell apoptosis kits. Representative images are shown. Quantification of flow cytometric analysis of apoptosis. ** *p* < 0.01, *** *p* < 0.001, **** *p* < 0.0001. **G** and **H** Western blot analysis of the effect of mTOR on autophagy markers in A549 and H1299 cells treated with the combination strategy. Cells were treated with the indicated supplements for 48 h in the absence or presence of 3BDO (100 *µ*M) or MHY1485 (10 *µ*M). LC3, p62 and Beclin1 proteins were detected by immunoblotting. GAPDH was used as internal control. CQ, chloroquine. DMSO was used for the control group

We observed morphological changes in the cells after treatment with BA and gefitinib/osimertinib under a light microscope. The combination treatment resulted in apparent vacuolization in the cytoplasm (Fig. 2g and h), and the morphological changes became more obvious with increasing treatment time. To investigate whether cytoplasmic vacuolization is caused by autophagy, we first identified whether the treatment could induce autophagy. The expression of LC3 was analyzed. It is an important autophagy-related protein that exists as LC3-I and a phosphatidylethanolamine conjugate (LC3-II) in cells. During autophagy, the cytosolic form of LC3-I is converted to the phagophore (the autophagosome precursor) membrane-bound LC3-II [43], which is an autophagic biomarker and can be used to monitor autophagic activity. As shown in Fig. 3a and b and S11, BA and gefitinib/osimertinib treatment significantly augmented the expression levels of LC3-II in A549 and H1299 cell lines, and the treatments also resulted in a significant decrease in the expression levels of the key autophagy genes p62/SQSTM1 and Becline1. These results indicated that the autophagy level was significantly greater in the treated cells than in the control cells and that exposure to the combination strategy could cause autophagy in NSCLC cells.

The effect of combined treatments on autophagy in NSCLC cells was explored. To assess autophagic flux, the conversion of LC3-I to LC3-II in the presence of an autophagy inhibitor was analyzed [44]. We found that combination treatment with chloroquine (CQ), which blocks the later steps of autophagy [45], led to further accumulation of LC3-II (Fig. 3c and d), indicating that the combination treatment promoted autophagic flux in NSCLC cells. To further verify the effect of the treatment on autophagic flux, we examined the protein expression levels of p62, which is a common readout of autophagic activity [46]. Similar to the enhanced LC3-II turnover, combination with CQ resulted in a marked accumulation of p62 compared with treatment alone, suggesting that autophagy inhibitors blocked combined treatment-triggered autophagic flux in NSCLC cells. 3-MA, an early-stage autophagy inhibitor, was used to inhibit the formation of autophagosomes, and the effect of 3-MA on combination strategy-induced autophagy was explored. Pretreatment with 5 mM 3-MA inhibited the conversion of LC3-I to LC3-II and significantly rescued the expression of Beclin1. Moreover, cotreatment with the autophagy blocker CQ or 3-MA completely eliminated combined strategy-triggered cytoplasmic vacuolization (Fig. S7). These lines of evidence suggested that the combination treatment could increase autophagy flux and induce cytoplasmic vacuolization.

Previously, BA was found to mediate apoptosis, while all the findings of the present study revealed that the combination strategy was an inducer of autophagy. The interplay between autophagy and apoptosis is complicated and differs with cell type and specific stress-causing factors of cell death. To investigate the role of autophagy in the combined treatment-induced antitumor effect in NSCLC, we blocked autophagy with an autophagy inhibitor. Flow cytometry analysis revealed that the administration of the autophagy inhibitor 3-MA notably enhanced combined treatment-triggered apoptosis (Fig. 3e and f). Consistently, the cleavage of the pro-apoptosis protein PARP was also greater in the 3-MA or CQ cotreatment group than in the combined treatment group (Fig. S8). Then, we evaluated the cytotoxic effect, as shown in Fig. S9. Compared with the combination strategy, pharmacological inhibition of strategy-induced autophagy with 3-MA or CQ profoundly attenuated the cytotoxic effect. Taken together, these results demonstrated that the blockade of autophagy promoted combined treatment-mediated apoptosis and that the occurrence of cell death depended on autophagy in NSCLC cells.

The signaling pathways regulated by EGFR (PI3K-AKT-mTOR, RAF-MEK-ERK, and STAT3-PKR-ELF2) have been implicated in the modulation of autophagy [17, 47]. BA was reported to induce autophagy by inhibiting AKT-mTOR signaling in colorectal cancer cells [48]. To further elucidate the molecular mechanisms of combination strategy-triggered autophagic cell death, we explored whether the treatment could induce lung cancer cell autophagy through the inhibition of PI3K-AKT-mTOR signaling. mTOR is regarded as a master regulator of autophagy due to its energy sensing function and has been identified as a key negative regulator of autophagy [47]. We activated EGFR-PI3K-AKT-mTOR pathway with mTOR activators (3BDO and MHY) followed by combined treatments and then examined the expression of LC3-II in the presence or absence of mTOR activators using immunoblotting analysis. Consistent with this hypothesis, as shown in Fig. 3g and h, pretreatment with 3BDO or MHY markedly reduced the conversion of LC3-II compared with the combined strategy alone and weakened cytoplasmic vacuolization (Fig. S10), suggesting that the strategy-induced accumulation of LC3-II depended on inhibition of the EGFR-PI3K-AKT-mTOR signaling pathway. Furthermore, to confirm whether other EGFR-related pathways were involved, immunoblotting analysis of p-Erk and p-STAT3 was performed. Both RAF-MEK-ERK and STAT3-PKR-ELF2 can positively regulate autophagy, and combination therapy decreases the phosphorylation of Erk and STAT3, suggesting the possibility of regulating autophagy through these two pathways. Together, these findings suggest that combination treatment could directly promote autophagy and autophagic flux in lung cancer cells through the EGFR-PI3K-AKT-mTOR signaling pathway. Moreover, autophagy contributed to combination treatment-induced cell death in NSCLC.

### Combination treatment facilitates EGFR-mediated G0/G1 cell cycle arrest

**Fig. 4:**
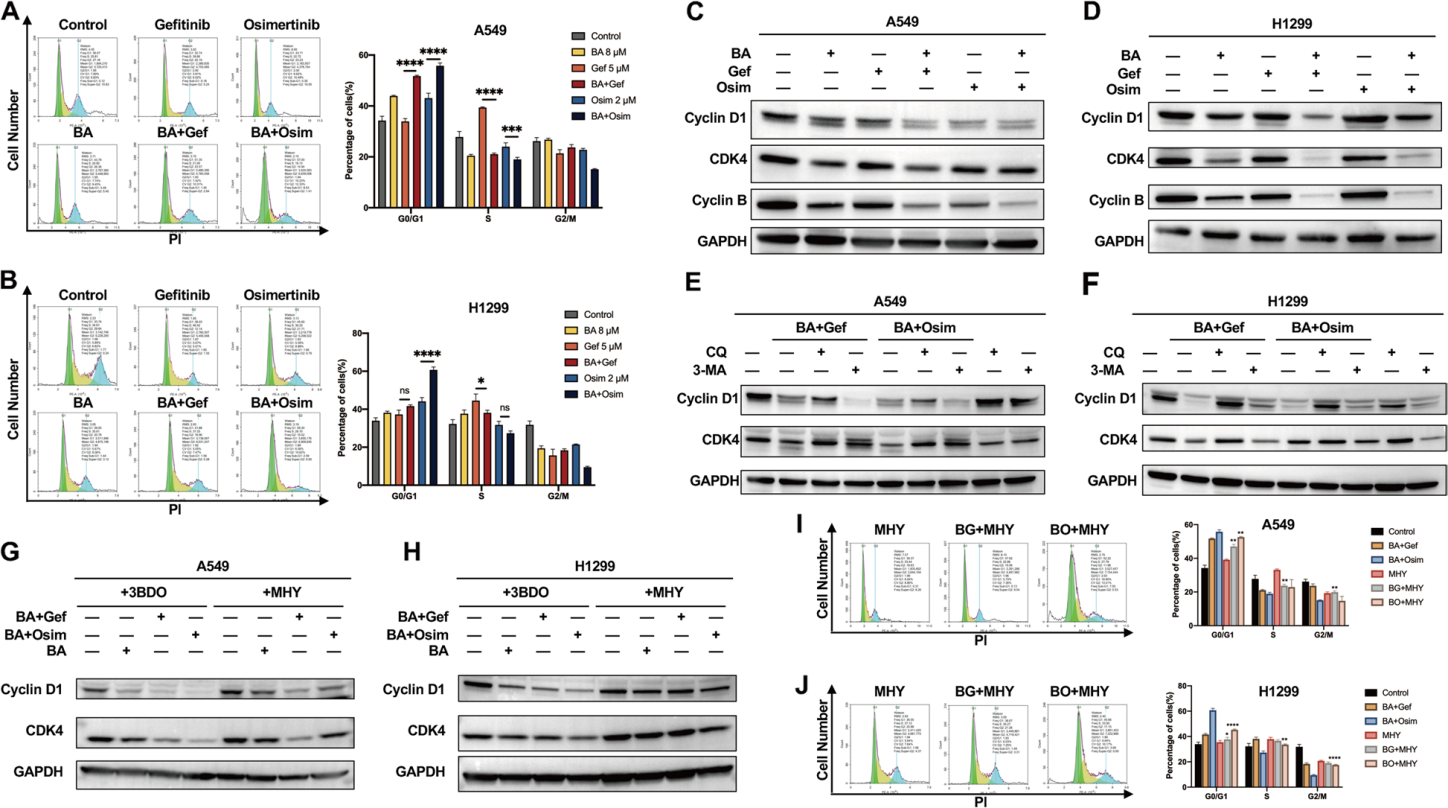
Combination treatment arrested cell cycle via EGFR-AKT-mTOR pathway. **A** and **B** Effect of the combination treatment on the cell cycle distribution of A549 and H1299 cells. All the data are presented as the means ± SDs (*n* = 3, * *p* < 0.05, *** *p* < 0.001, **** *p* < 0.0001). **C** and **D** Effect of the combination treatment on the protein levels of cell cycle markers. A549 and H1299 cells were treated with the indicated supplements for 48 h. Western blot assays were conducted with specific antibodies against Cyclin D1, CDK4 and Cyclin B1. GAPDH was used as internal control. **E** and **F** A549 and H1299 cells were treated with the indicated supplements for 48 h in the absence or presence of chloroquine (10 *µ*M) or 3-MA (5 mM). Cyclin D1 and CDK4 proteins were detected by immunoblotting. GAPDH was used as internal control. **G** and **H** Western blot analysis of the effect of mTOR on autophagy markers in A549 and H1299 cells treated with the combination strategy. Cells were treated with the indicated supplements for 48 h in the absence or presence of 3BDO (100 *µ*M) or MHY1485 (10 *µ*M). Western blot assays were conducted with specific antibodies against Cyclin D1 and CDK4. GAPDH was used as internal control. **I** and **J** A549 and H1299 cells were treated with MHY1485 (10 *µ*M) for 48 h in the presence of the indicated supplements. The cell cycle distribution was analyzed by flow cytometry. All the data are presented as means ± SDs (*n* = 3, * *p* < 0.05 ** *p* < 0.01, **** *p* < 0.0001)

Autophagy has been confirmed to be linked to cell cycle progression, exhibiting disparate biological effects under different conditions by removing key cell cycle regulators and other signaling adaptors [49]. To explore whether combination strategy-triggered autophagy impacted the cell cycle and further investigate the mechanism underlying the treatment-induced suppression of proliferation in NSCLC cells, the cell cycle distributions of A549 and H1299 cells were determined by flow cytometry after combined strategy treatment. Our results showed that cotreatment reduced the proportion of cells in the G2/M phase and blocked the G1 to S cell cycle transition in both cell lines, especially when BA combined with osimertinib was used (Fig. 4a and b). The impact of the treatments on cell cycle-related factors was analyzed, as shown in Fig. 4c and d. Consistent with the distribution of cells in different cell cycles, cotreatment downregulated the expression of the G1/S-phase transition regulator proteins Cyclin D1 and CDK4. Moreover, the level of Cyclin B, which is required for M-phase progression, was decreased in both cell lines. Since autophagy directly cross-talks with the cell cycle, to better define the autophagy (in)dependent role of autophagy in cell cycle arrest, we attempted to clarify whether cyclin D1 and CDK4 are regulated by autophagy. The cells were treated with the autophagy inhibitors (CQ and 3-MA) in combination for 48 h, and the immunoblotting results showed that the autophagy inhibitors reversed the changes in the expression levels of cyclin D1 and CDK4 (Fig. 4e and f). The above results showed that combined treatment triggered cell cycle arrest in G0/G1 phase, and this arrest could be mediated by autophagy.

To further evaluate the role of EGFR signaling pathway in regulating cell cycle arrest, MHY and 3BDO were employed as mTOR activators. The immunoblotting results showed that the mTOR activators increased the expression of cyclin D1 and CDK4 under the same combination treatment conditions (Fig. 4g and h). Furthermore, flow cytometric analysis confirmed that cotreatment with MHY markedly attenuated combination strategy-induced G0/G1 arrest. As shown in Fig. 4i and j, in the presence of MHY, the percentage of A549 cells in G0/G1 phase decreased significantly, from 51.97 to 47.02% (BA plus gefitinib) and from 55.77 to 52.56% (BA plus osimertinib), and these decreases were accompanied by increases in the percentage of cells in the S and G2/M phases. Similar effects were observed in H1299 cells, in which the percentage of G0/G1 cells decreased from 41.64 to 37.73% (BA plus gefitinib) and from 60.68 to 45.28% (BA plus osimertinib). Taken together, our data indicated that the combination strategy had remarkable antiproliferative effects on both A549 and H1299 cells and that the autophagic degradation machinery induced by inactivating EGFR-AKT-mTOR signaling is partially involved in the regulation of cell cycle progression via the degradation of cyclin D1 and CDK4.

### Combination treatment synergistically inhibits signaling pathways associated with resistance to EGFR TKIs

**Fig. 5:**
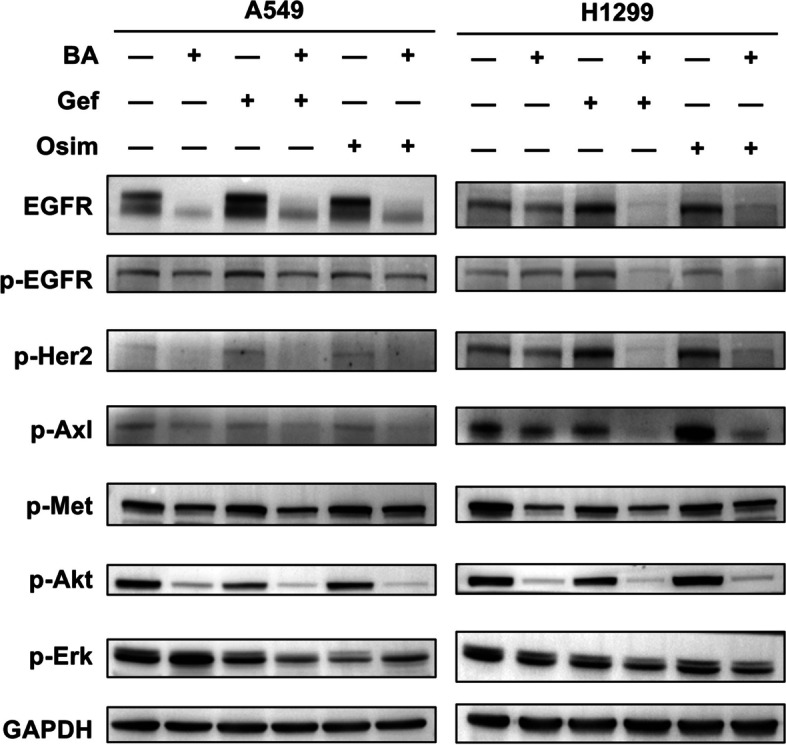
Combination treatment enhanced the suppression of EGFR and its downstream signaling and bypass pathways in wt-EGFR NSCLC cells. A549 and H1299 cells were treated with DMSO, BA, gefitinib, osimertinib or the indicated combination and then harvested for preparation of whole-cell protein lysates and subsequent western blotting to detect the indicated proteins

Tumor cell alterations conferring resistance to EGFR-TKIs may occur via proteins other than the targeted oncoprotein. It is well known that bypass receptor tyrosine kinase amplification and activation of compensatory signaling pathways are involved in resistance to targeted therapies. The off-target alterations could activate signaling downstream or in parallel to the targeted EGFR protein, sustaining oncogenic pathways and favoring cancer cell survival and growth. To further understand the benefit of BA in combination with gefitinib and osimertinib and to determine the molecular basis of the sensitization of primary TKI-resistant NSCLC cells to EGFR-TKIs, we investigated the effects of BA, gefitinib/osimertinib, and their cotreatment on EGFR bypass pathways [50]. Treatment with BA alone (8 *µ*M), without gefitinib (5 *µ*M) or osimertinib (2 *µ*M) for 48 h significantly inhibited the expression of p-Her2, p-Axl and p-Met in A549 cells, and the addition of gefitinib (5 *µ*M) or osimertinib (2 *µ*M) potentiated the inhibitory effect on p-EGFR, p-Her2, p-Axl and p-Met in H1299 cells (Fig. 5). The phosphorylation of Her2, Met and Axl receptors activates downstream effectors such as RAS-RAF-MER-ERK-MAPK and PI3K-AKT-mTOR, which are major EGFR downstream signaling pathways, and their activation initiates the proliferation and migration of tumors [51, 52]. Additionally, the activation of bypass pathways, including Her2, Axl, and c-Met pathways, is thought to play important roles in NSCLC resistance to EGFR-TKIs. These results suggested that BA could reverse NSCLC cell resistance to TKIs by interfering with multiple bypass receptor tyrosine kinases and cell proliferation pathways.

EGFR regulates multiple signaling pathways via the activation of downstream kinases, such as the ERK/MEK and AKT/mTOR pathways. We then evaluated the effects of BA combined with gefitinib/osimertinib on EGFR downstream signaling pathways. Cotreatment was more potent than either agent alone in decreasing the levels of p-Erk1/2 (Thr202/Tyr204) and p-Akt (S473) in both cell lines (Fig. 5). The results indicated that the combination treatments enhanced the suppressive effect on both EGFR bypass pathways and downstream tyrosine kinases that play key roles in the pro-oncogenic signaling of NSCLC, thereby potentiating the inhibitory effects of EGFR-TKIs on innate resistant NSCLC cells.

### BA synergizes with gefitinib to inhibit the proliferation of NSCLC cells in vivo

**Fig. 6:**
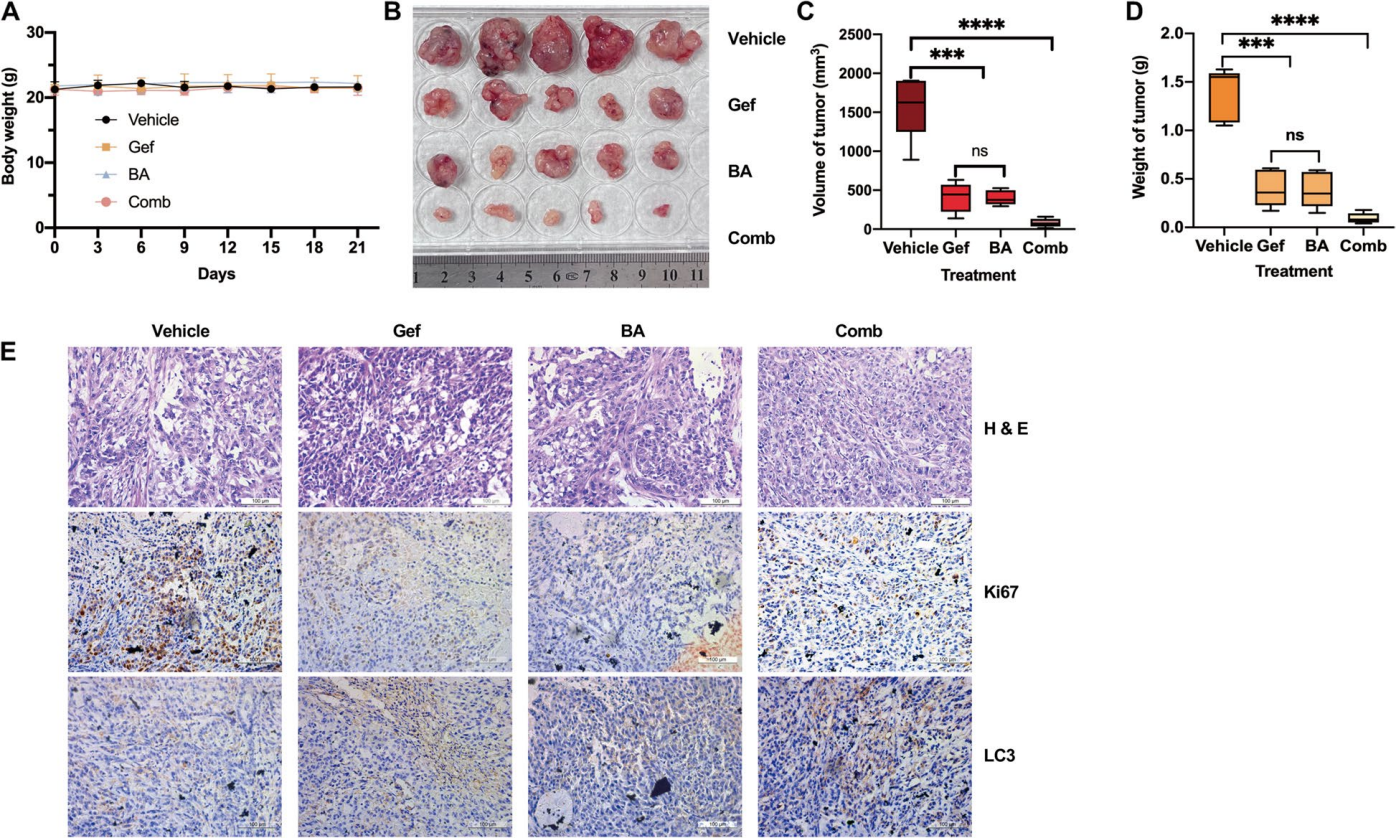
Combination treatment suppressed A549 xenograft growth in immunodeficient mice. **A** The body weight was quantified in each group. **B-D** Combination treatment represses tumor growth, as estimated by the tumor size (**B**), tumor volume (**C**), and tumor weight (**D**). **E** H&E staining of tumors and immunohistochemical staining of Ki67 and LC3 (scale bar, 100 *µ*m) in the tumors

To assess whether BA potentiated the in vivo antitumor activity of gefitinib in primary resistant NSCLC cell tumors, A549 cells were inoculated into BALB/c nude mice by subcutaneous injection to establish NSCLC cell xenograft mouse models. Fourteen days after injection, the tumor-bearing mice were randomly divided into four treatment groups (*n* = 5/group): the control group, BA treatment (30 mg/kg) group, gefitinib (30 mg/kg) treatment group and combination of two drugs group. BA was dissolved in DMSO and diluted in water with 0.5% carboxymethylcellulose (Meilunbio) and Tween-80 (Sigma), and gefitinib was dissolved in water. The weight of the mice was measured every three days. After treatment, the mice were euthanized, and the tumors were harvested. BA alone or gefitinib alone inhibited tumor growth to a greater extent than did treatment with vehicle. Compared with treatment with either drug alone, combination treatment with the two drugs significantly reduced tumor growth, both in terms of tumor volume and tumor weight (Fig. 6b-d).

Moreover, the combined strategy inhibited proliferation and induced autophagic death in lung cancer tumors. IHC revealed fewer Ki67-positive cells in tumors treated with BA or gefitinib than in control tumors. Correspondingly, Ki67 expression also sharply decreased when both agents were co-administered (Fig. 6e), and the protein level of LC3-II in the combination group was clearly greater than that in other groups, which was in accordance with the results of in vitro experiment. Moreover, there were no obvious changes in the body weights of the mice after treatment with BA or gefitinib alone or in combination (Fig. 6a), suggesting the tolerance and efficacy of the combination treatment. Collectively, consistent with the results of in vitro studies, the results confirmed that the combination of an EGFR inhibitor induced autophagy and acted synergistically to suppress the growth of primary resistant NSCLC tumors in vivo.

## Discussion

NSCLC is the leading cause of cancer-related death and accounts for 85% of malignant lung tumors [1]. Although EGFR-TKIs are clinically effective in NSCLC patients with EGFR oncogene mutations, wt-EGFR tumors are still a major clinical challenge for NSCLC therapy. The presence and emergence of drug resistance have highlighted the continued need for combination therapies, and effective agents are still needed to inhibit NSCLC progression. In this study, we confirmed that BA, a potential EGFR inhibitor, markedly inhibited wild-type EGFR-related NSCLC carcinogenesis. We designed a combinatorial strategy and showed that the combination of BA and EGFR-TKIs (gefitinib and osimertinib) exerted synergistic antitumor effects on intrinsically resistant NSCLC cells both in vitro and in vivo. This process induces EGFR-triggered autophagic cell death, which also causes cell cycle arrest, and the potent reversal of EGFR-TKI primary resistance is dependent on the regulation of EGFR signaling.

The use of BA and its derivatives, a class of high-profile bioactive agents, has attracted increasing interest owing to their multiple targets and broad-spectrum anticancer activities. Current studies have shown that BA mediates the selective killing of tumor cells by inhibiting proliferation, migration and invasion and has no cytotoxic effect on normal cells. Due to these attractive properties, the National Cancer Institute selected it as a candidate natural product-based agent for cancer treatment, and native Americans use it as a folk remedy [19]. BA has many pharmacological effects, including tumor suppression and anti-inflammatory effects. It has been shown to inhibit Sp1 and induce autophagy and apoptosis in lung cancer cells [20, 53–55]. In our study, we demonstrated for the first time that BA mediates its anticancer effect via direct binding with EGFR and regulating cellular pathways in NSCLCs expressing wt-EGFR genes. However, a similar effect was not found in H1975, PC9 or H827 cell lines, indicating the highly potent selectivity of BA against wt-EGFR. Our original computational simulation demonstrated that BA fits into the ATP-binding pocket of wt-EGFR and interrupts the binding between EGFR and ATP with a binding energy of -8.8 kcal/mol. We confirmed the inhibitory effect of BA on EGFR in A549 and H1299 cells, and the data suggested that BA treatment decreased the phosphorylation of EGFR and inhibited the EGFR-PI3K-AKT and MAPK signaling pathways, which are sensitive to EGFR-TKI resistance. These findings indicate that BA, a potential EGFR inhibitor, strengthens the ability of gefitinib and osimertinib to inhibit the proliferation of NSCLC cells and enhances EGFR-mediated autophagy in intrinsically resistant lung cancer cell lines.

The clinical benefit of EGFR-TKIs sparks the discovery of more therapeutic strategies that can enhance the sensitivity and appropriate use of anti-EGFR therapies in NSCLC. Moreover, potential chemosensitizers are required to sensitize wt-EGFR NSCLC cells to EGFR-TKI treatment. Targeting the EGFR pathway with small molecules to inhibit intracellular tyrosine kinase activity has been regarded as a promising therapeutic strategy. Unfortunately, the therapeutic benefit of these treatment strategies is still limited by the innate resistance of wt-EGFR tumors. EGFR and all its downstream signaling pathways have been demonstrated to regulate autophagy [56], so inducing autophagy-related cell death to overcome primary resistance to anti-EGFR treatments is a reasonable strategy for cancer drug discovery. In our study, the efficacy of EGFR-TKIs was markedly enhanced by combination with BA due to the induction of autophagy-related cell death. Thus, understanding the functional association of autophagy with EGFR-TKI resistance may provide a promising therapeutic strategy for conferring sensitivity to TKIs. It should be noted that both TKIs and BA in the co-treatment strategy have good safety, which does not mean that the combination treatment is still the same. Both safety and efficacy need to be given enough attention. The development of delivery system will provide more options for optimizing tumor treatment strategy.

Autophagy, a cellular ‘self-eating’ degradation process, is critical for regulating cell survival and maintaining cellular homeostasis. Autophagic cell death has been observed in several tumors as a cell death mechanism accompanied by extensive cytoplasmic vacuolization and correlated with increased autophagic flux [57]. A functional study revealed associations between autophagy status and drug response, suggesting that cancer samples with high autophagy scores are more sensitive to drugs [58]. Moreover, autophagy has emerged as a potential mechanism involved in acquired resistance to anti-EGFR treatments [59]. Emerging evidence indicates that EGFR is one of the determinants of whether autophagy results in cell death or survival [39]. The PI3K-AKT-mTOR signaling pathway is a well-established negative regulator of autophagy, and the RAS-RAF-MEK-ERK cascade positively regulates the activity of many proteins involved in apoptosis and autophagy [47]. Previous studies have shown that both EGFR-TKIs and BA promote autophagy in many tumor types. In this study, compared with single drug-treated cells, TKI-resistant cells treated with a combination of BA and TKIs exhibited more significant increases in autophagy and autophagy flux without increasing the level of cleaved PARP. Inconsistent with previous findings in lung cancer [53], BA did not activate apoptosis or reduce the expression level of Sp1 in our research, suggesting that autophagy could play a certain role in the anticancer effect of BA. Cotreatment with the autophagy inhibitors CQ and 3-MA reversed autophagy and increased the two-drug combination-induced increase in cleaved PARP and apoptosis. The EGFR-mediated PI3K-AKT-mTOR signaling pathway plays a crucial role in cell functions, such as proliferation, cell cycle progression, cell death and autophagy. However, current knowledge on the role of EGFR-mediated activation of AKT-mTOR signaling pathway in autophagy induction is limited. Our data clearly indicated that the activation of mTOR can depress the increase of LC3B-II and reverse the decrease of p62 and Beclin1 induced by BA plus gefitinib or osimertinib, suggesting that the autophagy induced by the combination of these two drugs depends on the EGFR-PI3K-AKT-mTOR signaling pathway. Our findings reveal the relationship between EGFR tyrosine kinase activity and the induction of autophagy-mediated cancer cell death in NSCLC cells treated with combined strategies.

Cell death is a complex process of physiological quality control, and autophagy often accompanies other cell death modalities. It is necessary to determine the contribution of autophagy to the execution of cell death [43]. In this study, the combination of BA and TKIs increased both autophagy and autophagic flux. Although co-treatment with 3-MA slightly increased apoptosis, demonstrating that autophagy has a protective effect on combination-treated NSCLC cells, the inhibition of autophagy largely reversed the cell death induced by combination treatment. The mechanisms underlying the interaction between autophagy and apoptosis have not been elucidated, and we postulated that BA could mediate the crosstalk between autophagy and apoptosis via multi-targets [60, 61]. Moreover, the activation of EGFR bypass and downstream signaling pathways are also the main causes of TKI resistance. The phosphorylation of EGFR, Her2, Met and Axl was significantly inhibited in treated cells. In addition, the activity of EGFR downstream molecules, such as ERK1/2 and AKT, was also obviously diminished by the combination treatment. These results suggest that the autophagic cell death mediated by EGFR-AKT-mTOR signaling contributed to the combination-induced cell death in wt-EGFR NSCLC cells. Our findings demonstrated that promoting autophagy-related cell death by small molecules may be exploited to design better therapeutic strategies for wt-EGFR tumors.

Autophagy has been confirmed to regulate cell cycle progression by removing key cell cycle regulators and other signaling adaptors. Increasing evidence clearly demonstrates the interaction between cell cycle regulation and autophagy [49]; however, these findings often led to divergent rather than unifying explanations due to the complexity of the autophagy signaling network. Our results indicate that the combination strategy-induced increase in the sub-G0/G1 population and decrease in cyclin D1/CDK4 can be reversed by inhibiting autophagy. Blocking the EGFR-PI3K-AKT-mTOR signaling pathway with pharmacological agents (MHY) partly, but not completely, prevented combination treatment-induced cell cycle arrest, suggesting that other potential mechanisms of action for inducing arrest might be involved, and further investigation concerning the role of this treatment in lung cancer is needed. Recently, it was shown that CDK4 potentially triggers autophagy, and in our study, whether the cell cycle regulator affects autophagy is worth further study.

We found BA to be potent against wt-EGFR via a focused drug-screening protocol and confirmed its activity in vivo. The activity depended on affecting the ATP-binding site of the EGFR kinase domain, with stronger effects on wt-EGFR. The combination therapy with TKIs showed more preferable activity than monotherapy in vitro and in vivo without toxicity. Although the potency of BA monotherapy does not seem to be completely satisfactory, there is limited evidence that BA directly affects the ATP-binding site of wt-EGFR because of the absence of a co-crystal structure. More thorough understanding of the molecular mechanisms by which BA induces autophagy-related cell death will facilitate the development of the compound as a candidate therapeutic for treating lung cancer. BA strikingly reduced the IC_50_ of EGFR-TKIs against wt-EGFR by directly targeting EGFR to trigger EGFR-mediated autophagy-related cell death and cell cycle arrest, thus revealing a novel mechanism underlying the powerful antitumor effect of combined treatment in wt-EGFR NSCLC. Moreover, the co-inhibition of EGFR with combination strategy is also highlighted as a novel approach to improve cancer prognosis. The role and regulatory mechanisms of EGFR and its natural targeting agent BA warrant additional studies to ensure its eventual translation into clinical application.

## Conclusions

In conclusion, our study demonstrated that the natural product BA, a potential EGFR inhibitor, directly targets wt-EGFR and sensitizes innate TKI-resistant cancer cells to EGFR-TKIs. BA could chemosensitize wt-EGFR lung cancer cells to gefitinib/osimertinib through direct binding to EGFR, triggering PI3K-AKT-mTOR axis-mediated autophagic cell death, consequently inducing cell cycle arrest and causing marked tumor regression in vivo. Our findings highlight the potential clinical utility of BA in combination with EGFR-TKIs in wt-EGFR lung cancer, suggesting that targeting the EGFR-mediated autophagic pathway remains a viable therapeutic strategy to overcome primary resistance to EGFR-TKIs in wt-EGFR NSCLC. Taken together, our findings provide novel insight into the role and regulatory mechanisms of the natural targeting blocker BA of EGFR and a more thorough understanding of the molecular mechanisms by which BA and its combination induce autophagy-related cell death, which will ensure its eventual translation into clinical application and may identify a new therapeutic approach for wt-EGFR NSCLC patients.

## Supplementary Information

Supplementary Material 1.

Supplementary Material 2.

## Data Availability

No datasets were generated or analysed during the current study.
