# Correction: Combination of betulinic acid and EGFR‑TKIs exerts synergistic anti‑tumor effects against wild‑type EGFR NSCLC by inducing autophagy‑related cell death via EGFR signaling

**DOI:** 10.1186/s12931-025-03223-8

**Published:** 2025-04-13

**Authors:** Han Wang, Xiaohui Du, Wenwen Liu, Congcong Zhang, Ying Li, Jingwen Hou, Yi Yu, Guiru Li, Qi Wang

**Affiliations:** 1 The Second Hospital of Dalian Medical University, Dalian, 116023 China; 2 Guangzhou women and children’s medical center, Guangzhou Medical University, Guangzhou, 510623 China

**Correction to: Respiratory Research (2024) 25:215**

10.1186/s12931-024-02844-9

In the original publication of this article [1], there was an error in Fig. 5. The western blots lines reporting the EGFR and p-Her2 bands for H1299 cell lines were duplicated. The EGFR bands was mistakenly repeated in p-Her2 bands. For completeness and transparency, the old incorrect and correct Fig. 5 are displayed in this correction article.

**Incorrect Fig. 5**:

**Fig. 5:**
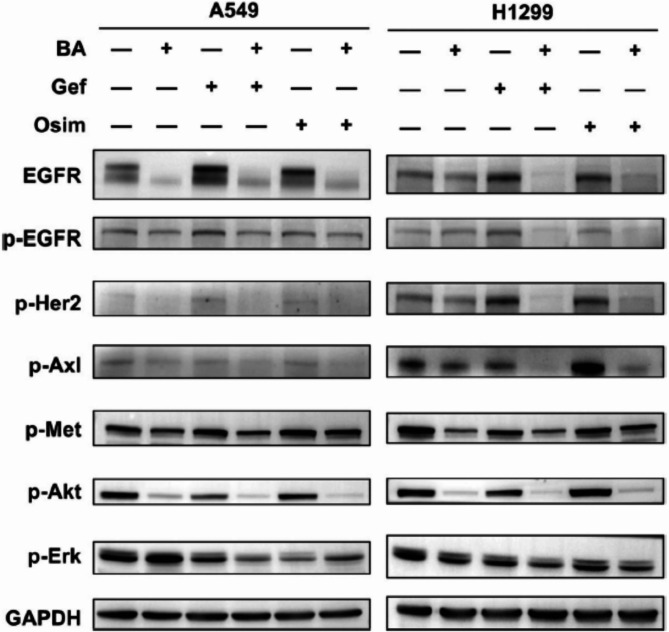
Combination treatment enhanced the suppression of EGFR and its downstream signaling and bypass pathways in wt-EGFR NSCLC cells. A549 and H1299 cells were treated with DMSO, BA, gefitinib, osimertinib or the indicated combination and then harvested for preparation of whole-cell protein lysates and subsequent western blotting to detect the indicated proteins

**Correct Fig. 5**:

**Fig. 5:**
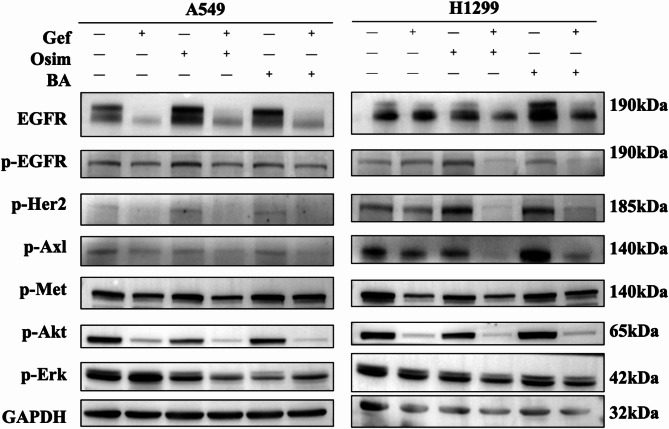
Combination treatment enhanced the suppression of EGFR and its downstream signaling and bypass pathways in wt-EGFR NSCLC cells. A549 and H1299 cells were treated with DMSO, BA, gefitinib, osimertinib or the indicated combination and then harvested for preparation of whole-cell protein lysates and subsequent western blotting to detect the indicated proteins
