# Supplementary material for: Combination of betulinic acid and EGFR-TKIs exerts synergistic anti-tumor effects against wild-type EGFR NSCLC by inducing autophagy-related cell death via EGFR signaling pathway
Source: Respir Res. 2024 May 20;25:215. doi: 10.1186/s12931-024-02844-9 (PMC11103851; doi:10.1186/s12931-024-02844-9)
Supplement: Supplementary file 1 — Supplementary Material 1. [file 12931_2024_2844_MOESM1_ESM.pptx]

## Slide 1
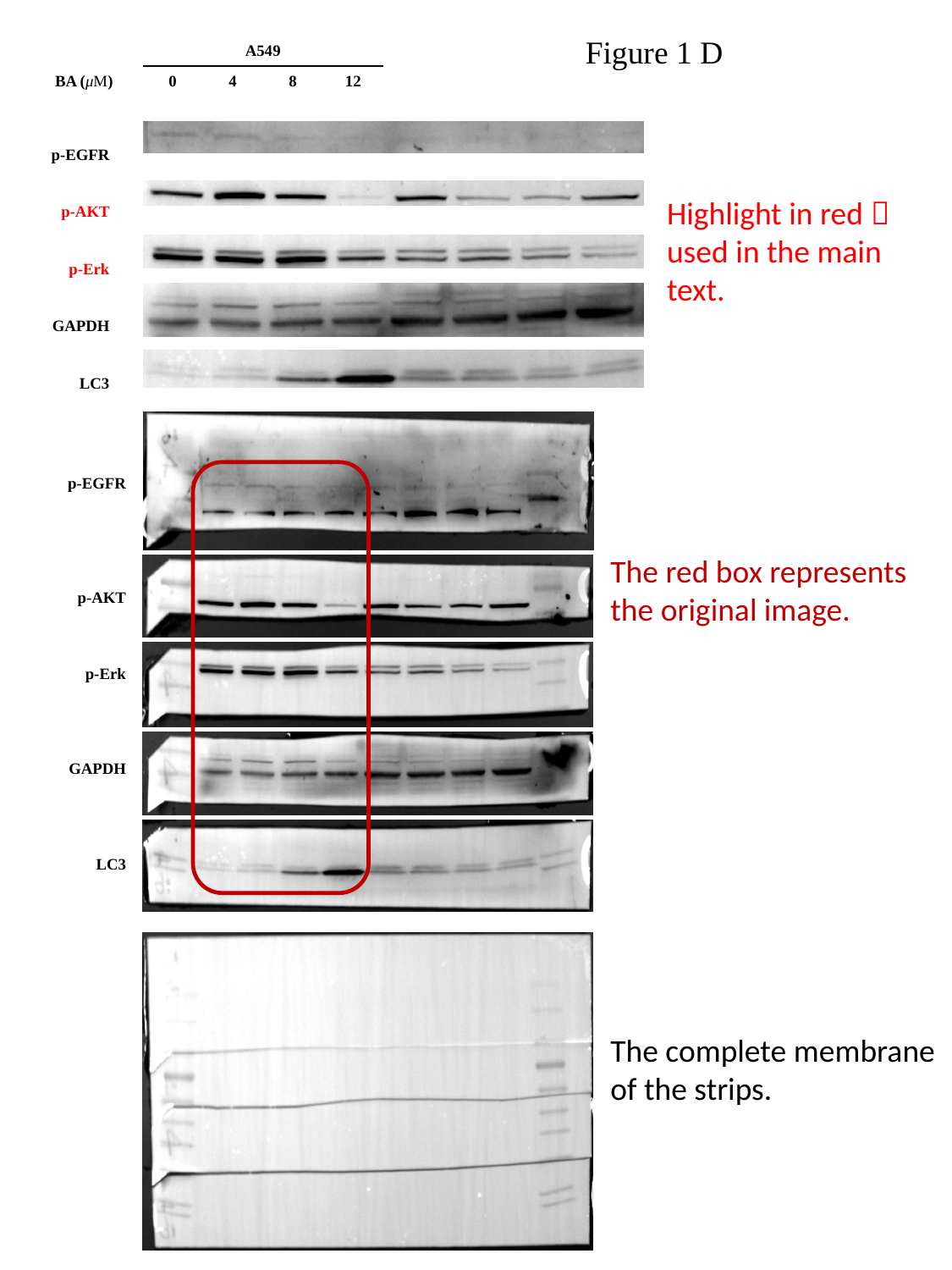

| | A549 | | | |
| --- | --- | --- | --- | --- |
| BA (μM) | 0 | 4 | 8 | 12 |
Figure 1 D
p-EGFR
p-AKT
p-Erk
GAPDH
LC3
Highlight in red：used in the main text.
p-EGFR
p-AKT
p-Erk
GAPDH
LC3
The red box represents the original image.
The complete membrane of the strips.

## Slide 2
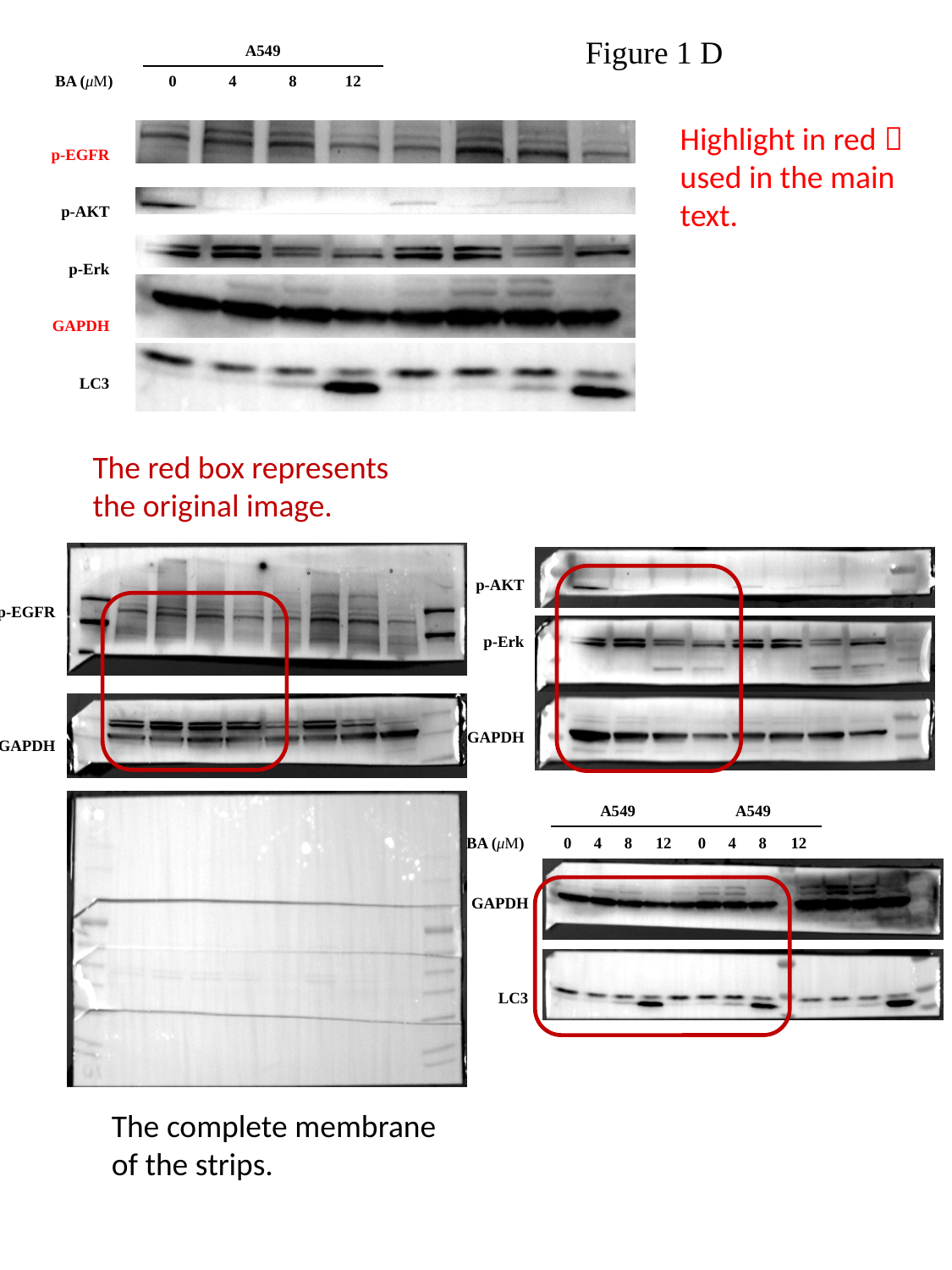

| | A549 | | | |
| --- | --- | --- | --- | --- |
| BA (μM) | 0 | 4 | 8 | 12 |
Figure 1 D
p-EGFR
p-AKT
p-Erk
GAPDH
LC3
Highlight in red：used in the main text.
The red box represents the original image.
p-AKT
p-Erk
GAPDH
 p-EGFR
GAPDH
| | A549 | | | | A549 | | | |
| --- | --- | --- | --- | --- | --- | --- | --- | --- |
| BA (μM) | 0 | 4 | 8 | 12 | 0 | 4 | 8 | 12 |
GAPDH
LC3
The complete membrane of the strips.

## Slide 3
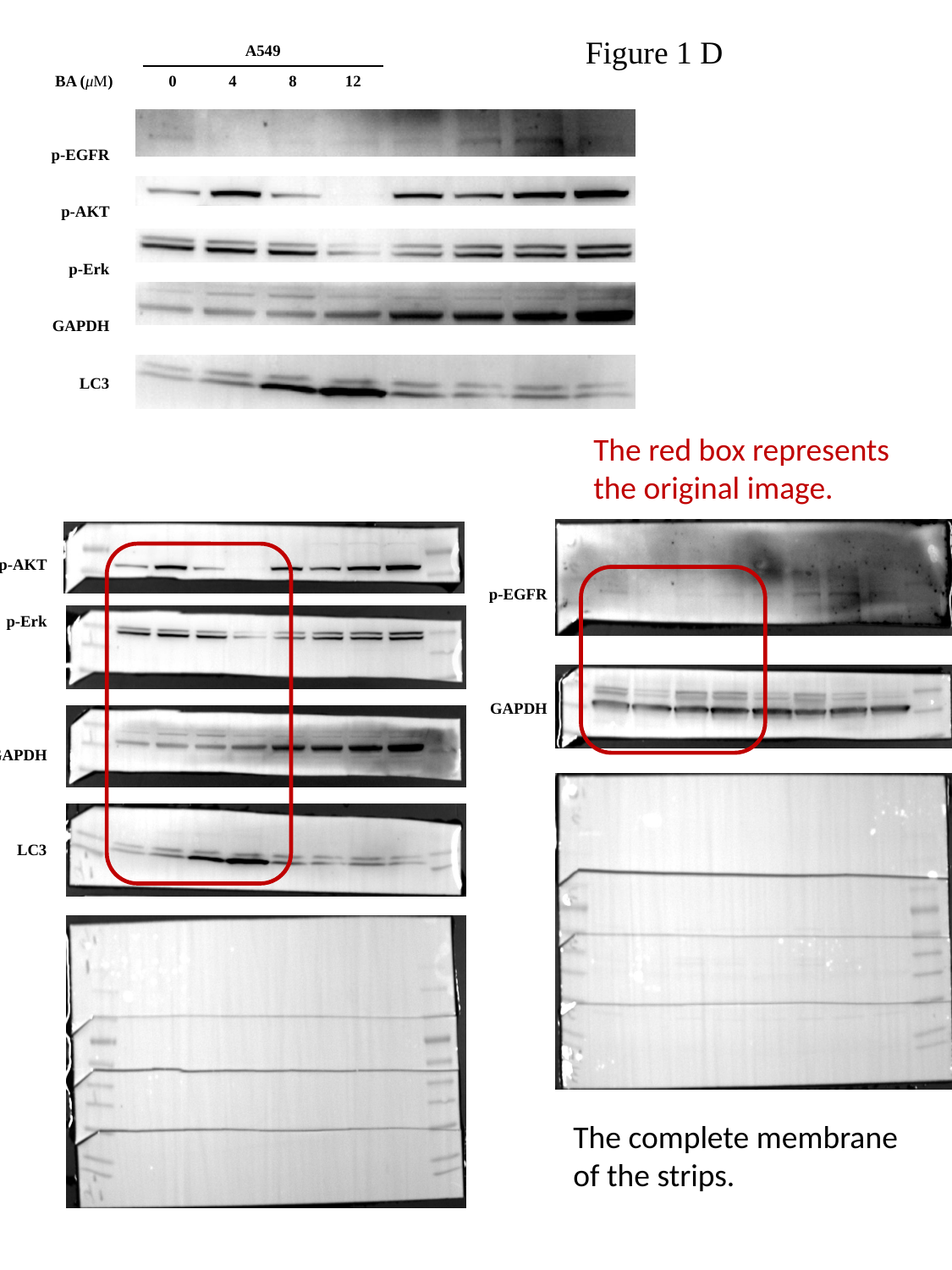

| | A549 | | | |
| --- | --- | --- | --- | --- |
| BA (μM) | 0 | 4 | 8 | 12 |
Figure 1 D
p-EGFR
p-AKT
p-Erk
GAPDH
LC3
The red box represents the original image.
p-AKT
p-Erk
GAPDH
LC3
p-EGFR
GAPDH
The complete membrane of the strips.

## Slide 4
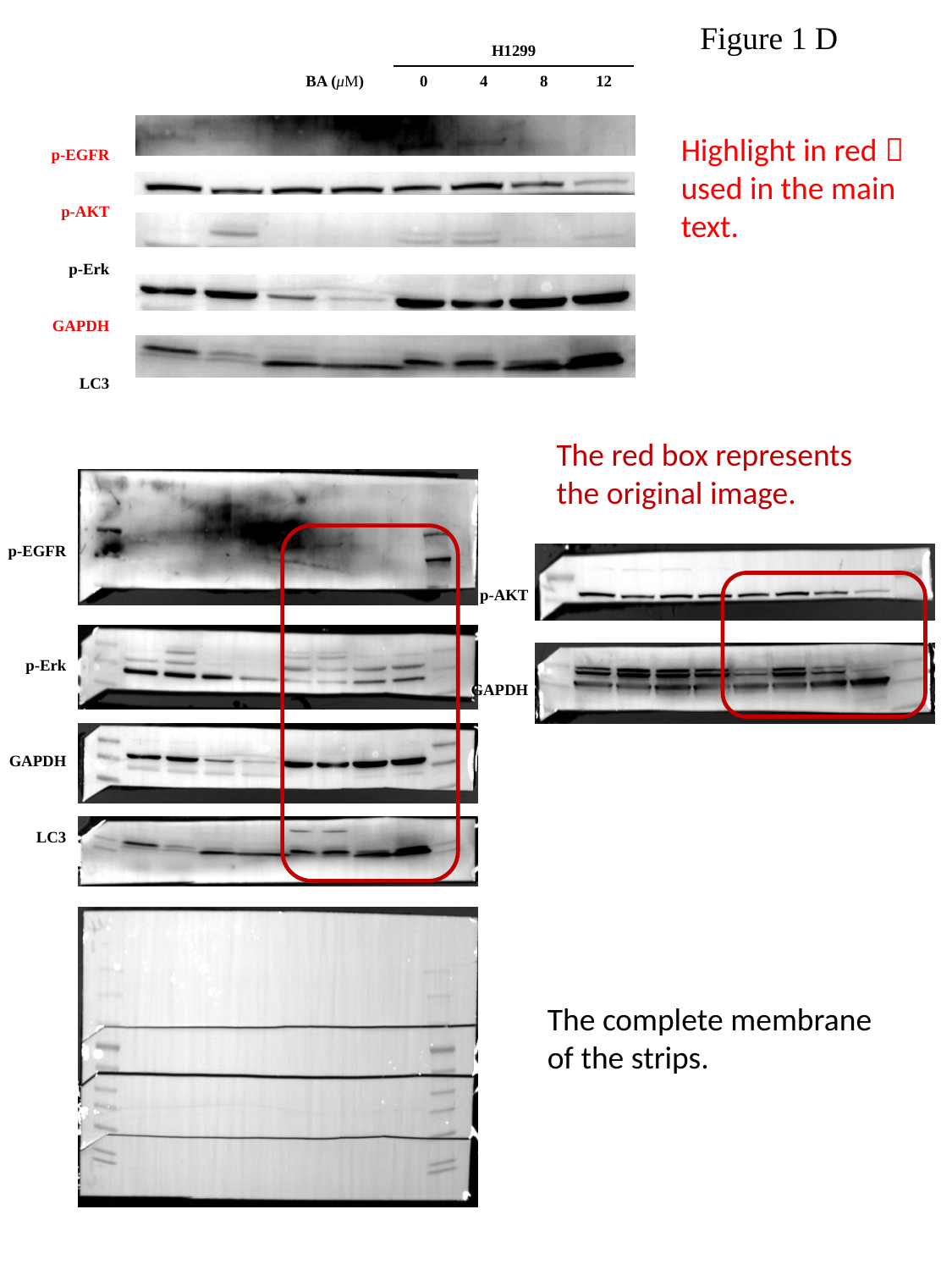

Figure 1 D
| | H1299 | | | |
| --- | --- | --- | --- | --- |
| BA (μM) | 0 | 4 | 8 | 12 |
p-EGFR
p-AKT
p-Erk
GAPDH
LC3
Highlight in red：used in the main text.
The red box represents the original image.
p-EGFR
p-Erk
GAPDH
LC3
p-AKT
GAPDH
The complete membrane of the strips.

## Slide 5
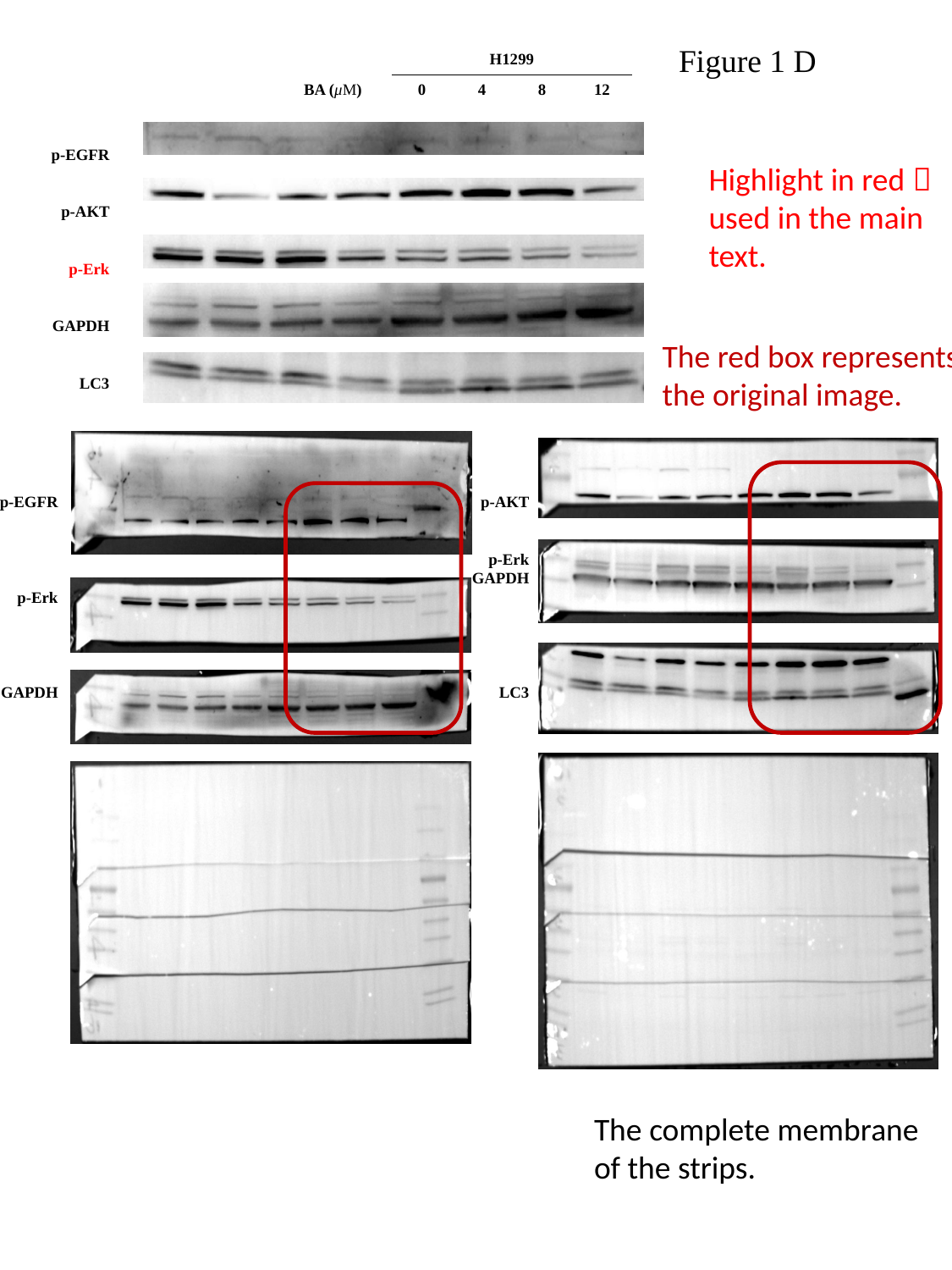

| | H1299 | | | |
| --- | --- | --- | --- | --- |
| BA (μM) | 0 | 4 | 8 | 12 |
Figure 1 D
p-EGFR
p-AKT
p-Erk
GAPDH
LC3
Highlight in red：used in the main text.
The red box represents the original image.
p-EGFR
p-Erk
GAPDH
p-AKT
p-Erk
GAPDH
LC3
The complete membrane of the strips.

## Slide 6
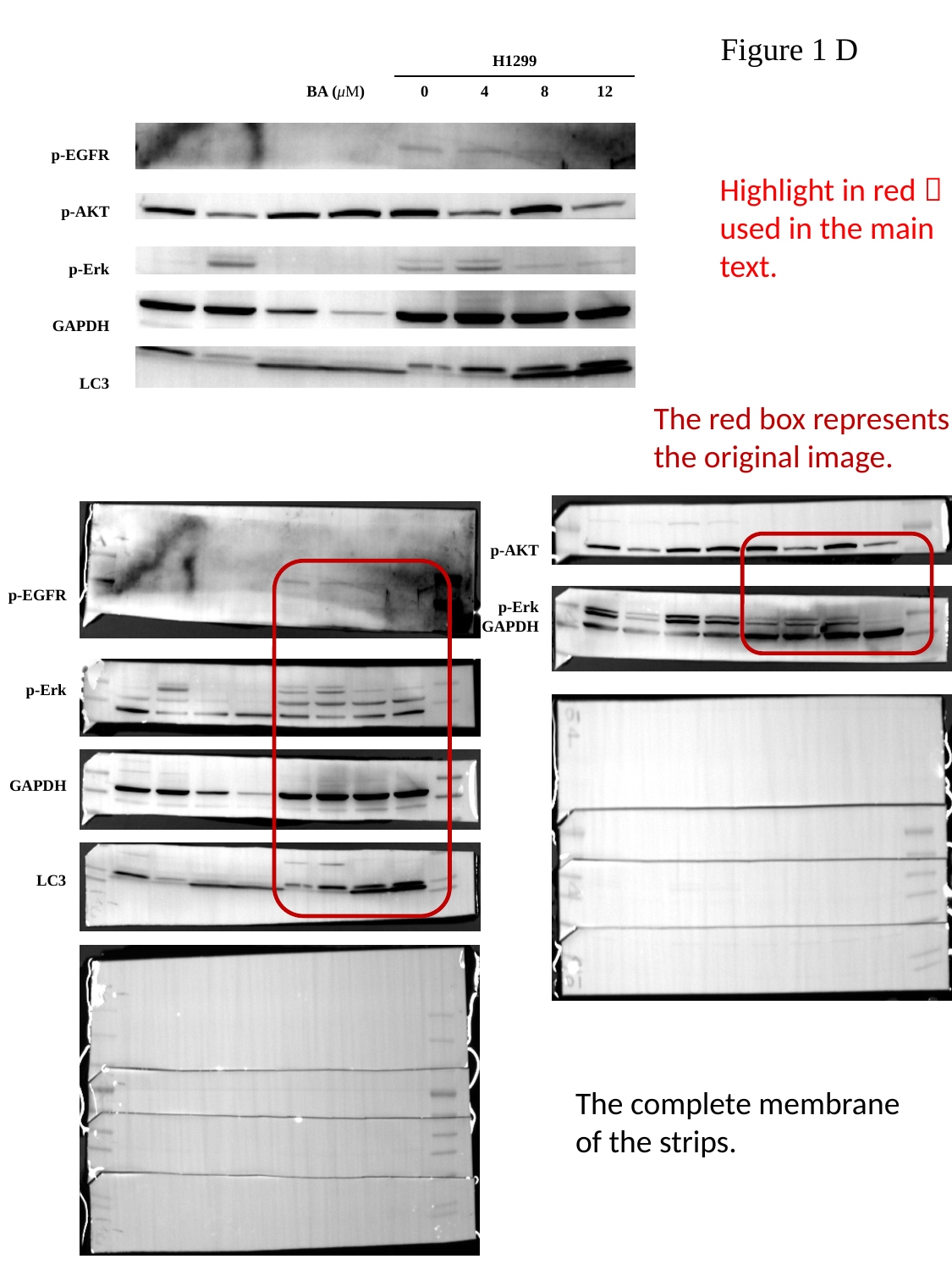

Figure 1 D
| | H1299 | | | |
| --- | --- | --- | --- | --- |
| BA (μM) | 0 | 4 | 8 | 12 |
p-EGFR
p-AKT
p-Erk
GAPDH
LC3
Highlight in red：used in the main text.
The red box represents the original image.
p-AKT
p-Erk
GAPDH
p-EGFR
p-Erk
GAPDH
LC3
The complete membrane of the strips.

## Slide 7
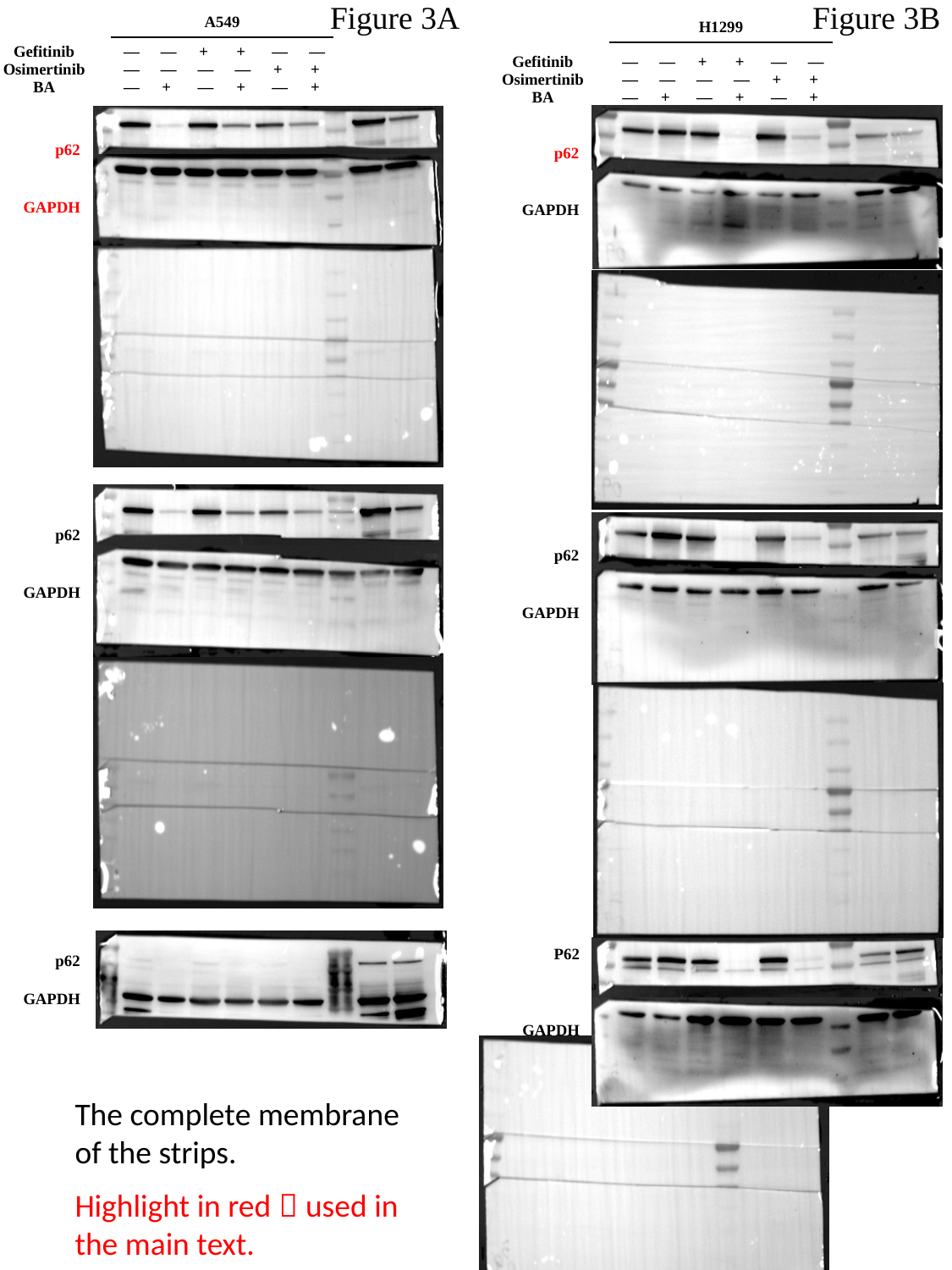

| | H1299 | | | | | |
| --- | --- | --- | --- | --- | --- | --- |
| Gefitinib Osimertinib BA | ——— | —— + | + —— | + — + | — + — | — + + |
Figure 3A
Figure 3B
| | A549 | | | | | |
| --- | --- | --- | --- | --- | --- | --- |
| Gefitinib Osimertinib BA | ——— | —— + | + —— | + — + | — + — | — + + |
p62
GAPDH
p62
GAPDH
p62
GAPDH
p62
GAPDH
p62
GAPDH
P62
GAPDH
The complete membrane of the strips.
Highlight in red：used in the main text.

## Slide 8
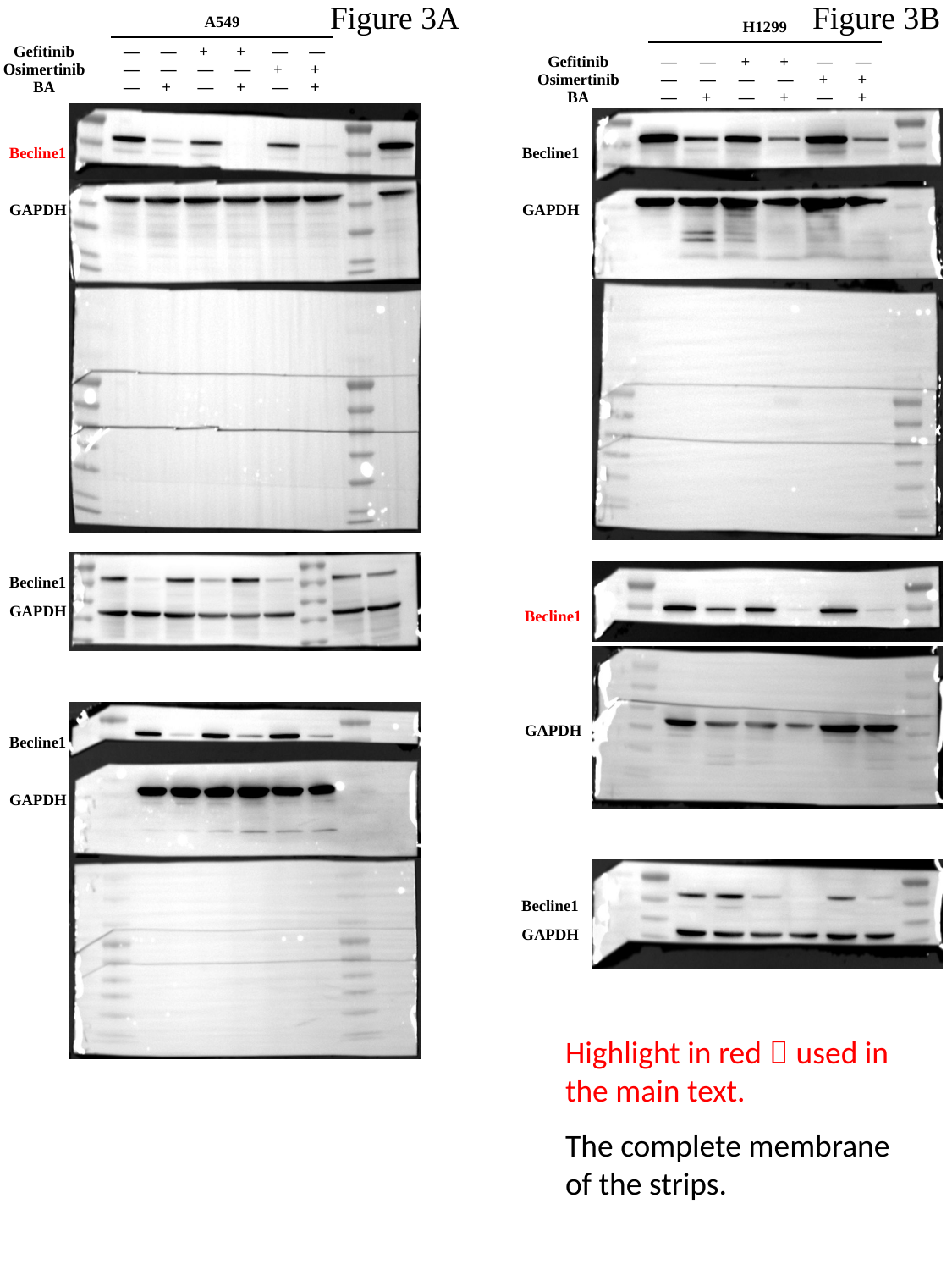

| | H1299 | | | | | |
| --- | --- | --- | --- | --- | --- | --- |
| Gefitinib Osimertinib BA | ——— | —— + | + —— | + — + | — + — | — + + |
Figure 3A
Figure 3B
| | A549 | | | | | |
| --- | --- | --- | --- | --- | --- | --- |
| Gefitinib Osimertinib BA | ——— | —— + | + —— | + — + | — + — | — + + |
Becline1
GAPDH
Becline1
GAPDH
Becline1
GAPDH
Becline1
GAPDH
Becline1
GAPDH
Becline1
GAPDH
Highlight in red：used in the main text.
The complete membrane of the strips.

## Slide 9
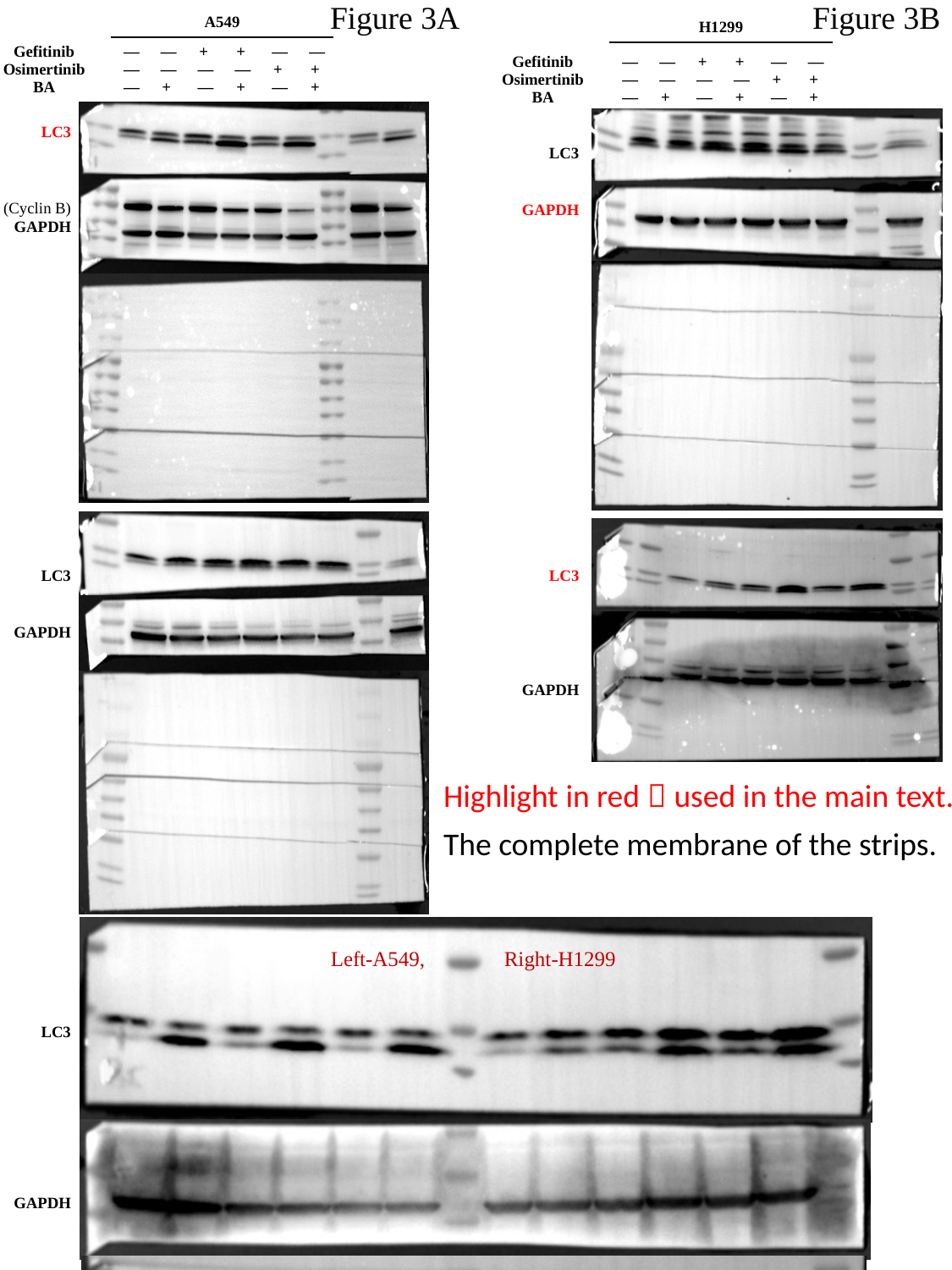

| | H1299 | | | | | |
| --- | --- | --- | --- | --- | --- | --- |
| Gefitinib Osimertinib BA | ——— | —— + | + —— | + — + | — + — | — + + |
Figure 3A
Figure 3B
| | A549 | | | | | |
| --- | --- | --- | --- | --- | --- | --- |
| Gefitinib Osimertinib BA | ——— | —— + | + —— | + — + | — + — | — + + |
LC3
(Cyclin B)
GAPDH
LC3
GAPDH
LC3
GAPDH
LC3
GAPDH
Highlight in red：used in the main text.
The complete membrane of the strips.
Left-A549, Right-H1299
LC3
GAPDH

## Slide 10
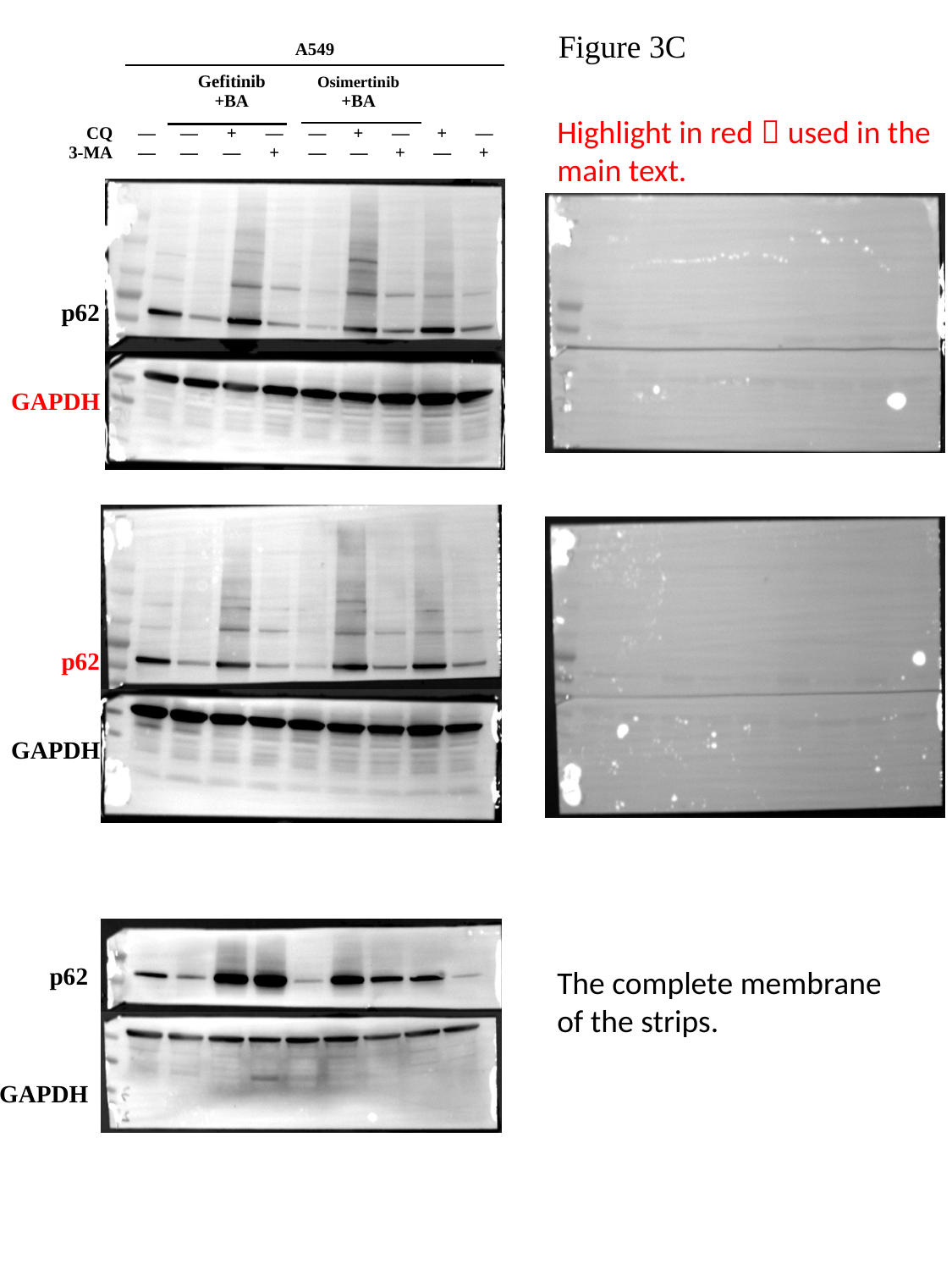

Figure 3C
| | A549 | | | | | | | | |
| --- | --- | --- | --- | --- | --- | --- | --- | --- | --- |
| | | Gefitinib +BA | | | Osimertinib +BA | | | | |
| CQ 3-MA | —— | —— | + — | — + | —— | + — | — + | + — | — + |
Highlight in red：used in the main text.
p62
GAPDH
p62
GAPDH
p62
GAPDH
The complete membrane of the strips.

## Slide 11
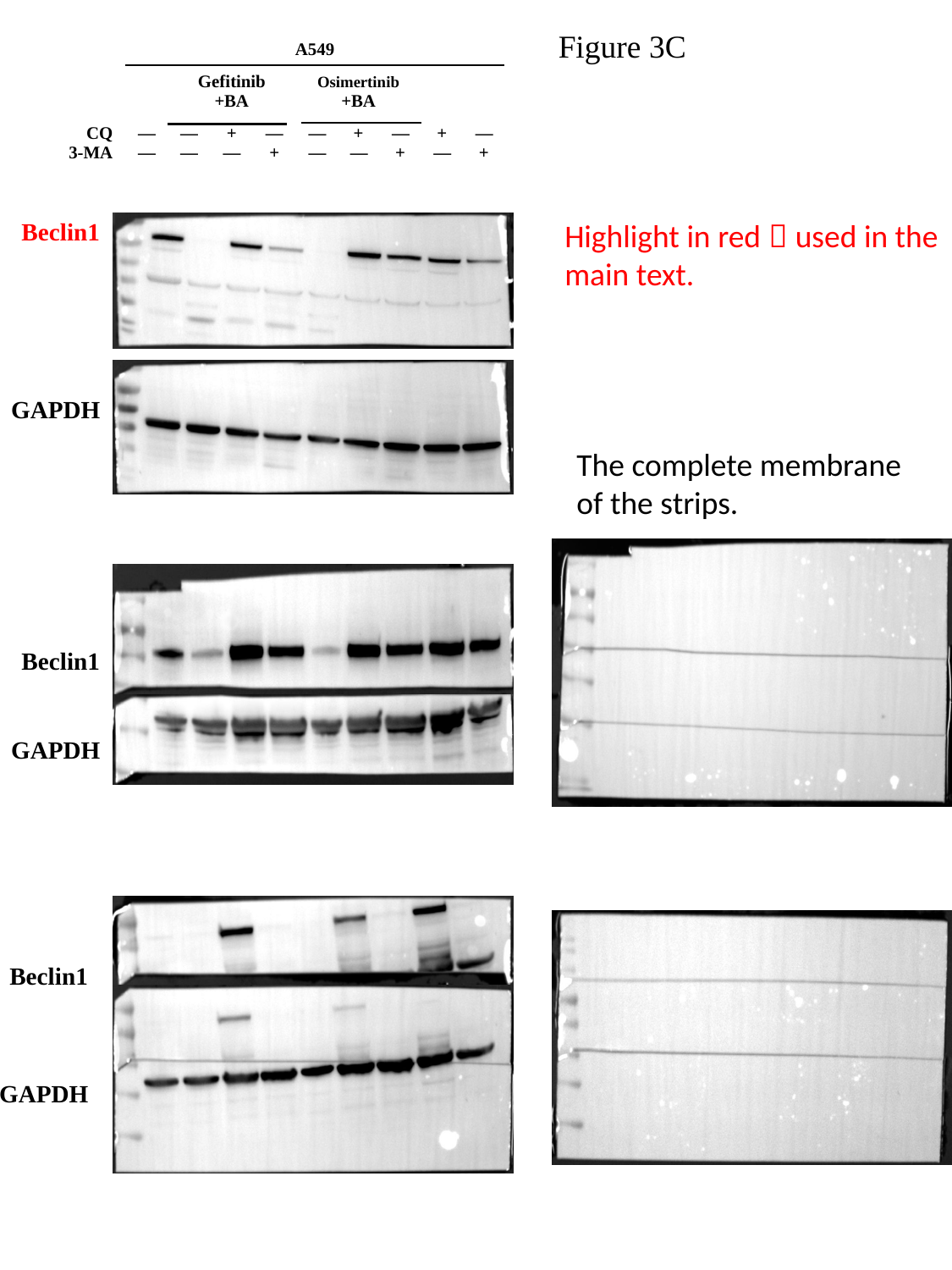

Figure 3C
| | A549 | | | | | | | | |
| --- | --- | --- | --- | --- | --- | --- | --- | --- | --- |
| | | Gefitinib +BA | | | Osimertinib +BA | | | | |
| CQ 3-MA | —— | —— | + — | — + | —— | + — | — + | + — | — + |
Beclin1
GAPDH
Highlight in red：used in the main text.
The complete membrane of the strips.
Beclin1
GAPDH
Beclin1
GAPDH

## Slide 12
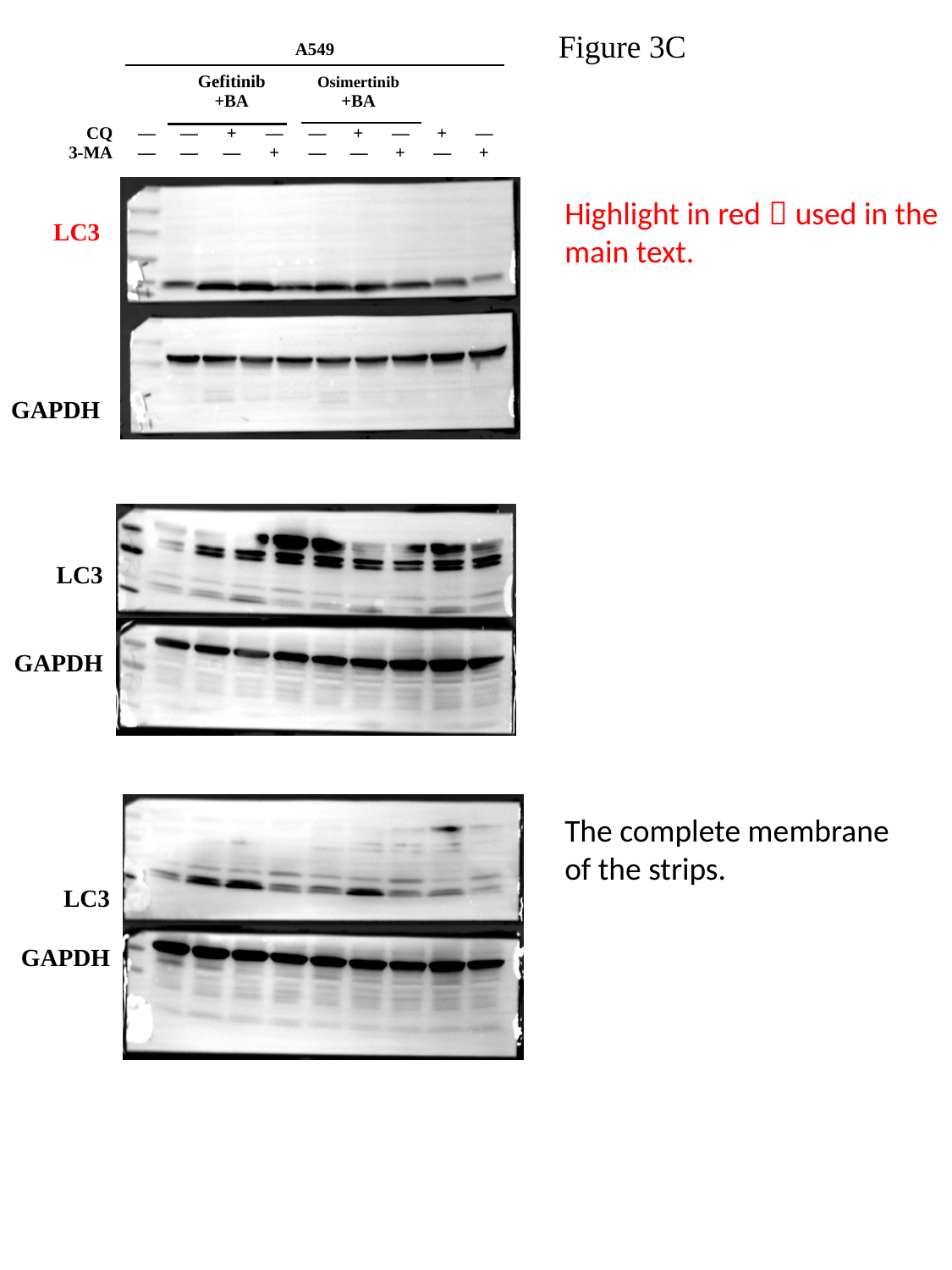

Figure 3C
| | A549 | | | | | | | | |
| --- | --- | --- | --- | --- | --- | --- | --- | --- | --- |
| | | Gefitinib +BA | | | Osimertinib +BA | | | | |
| CQ 3-MA | —— | —— | + — | — + | —— | + — | — + | + — | — + |
Highlight in red：used in the main text.
LC3
GAPDH
LC3
GAPDH
The complete membrane of the strips.
LC3
GAPDH

## Slide 13
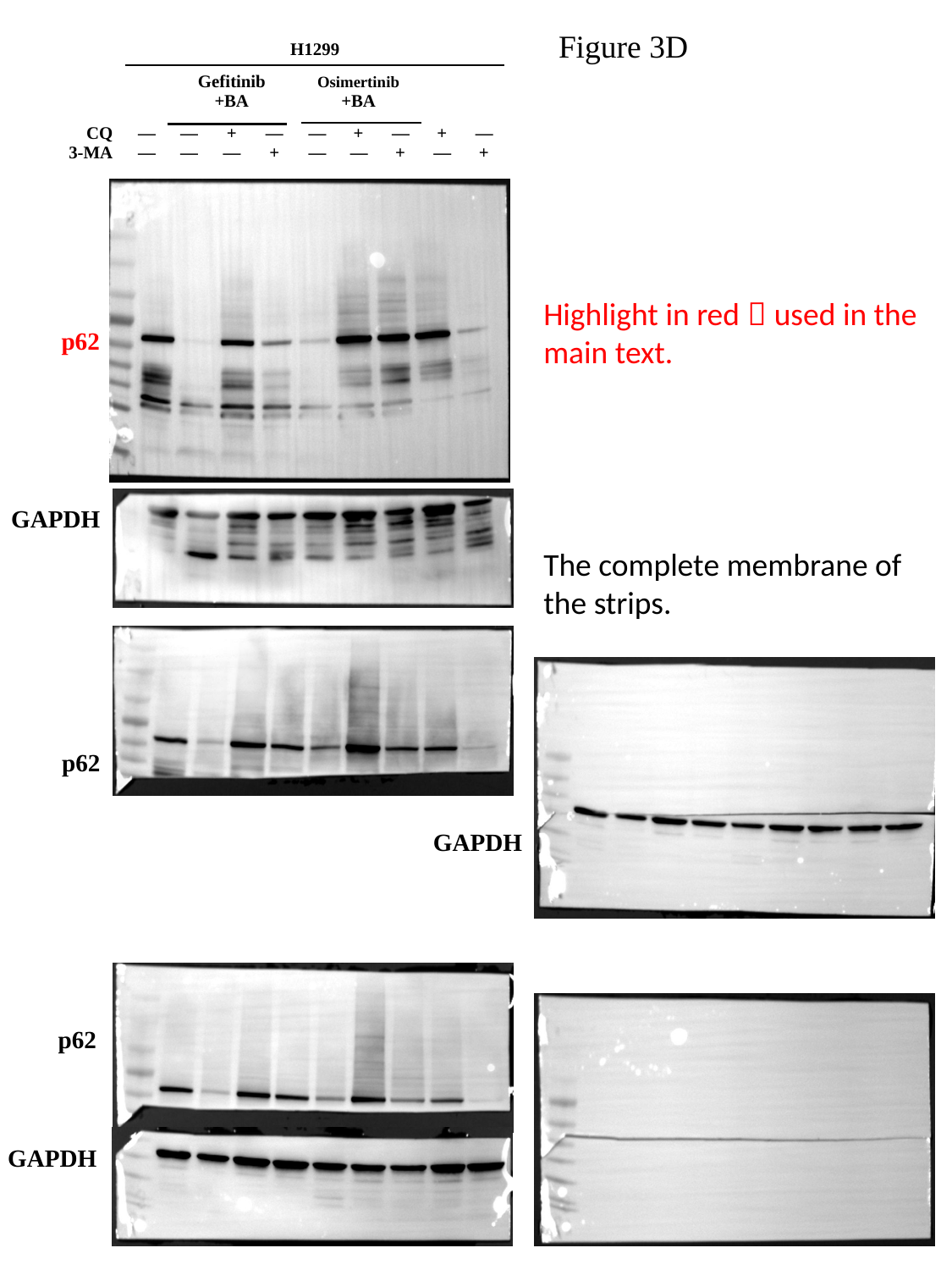

Figure 3D
| | H1299 | | | | | | | | |
| --- | --- | --- | --- | --- | --- | --- | --- | --- | --- |
| | | Gefitinib +BA | | | Osimertinib +BA | | | | |
| CQ 3-MA | —— | —— | + — | — + | —— | + — | — + | + — | — + |
Highlight in red：used in the main text.
p62
GAPDH
The complete membrane of the strips.
p62
GAPDH
p62
GAPDH

## Slide 14
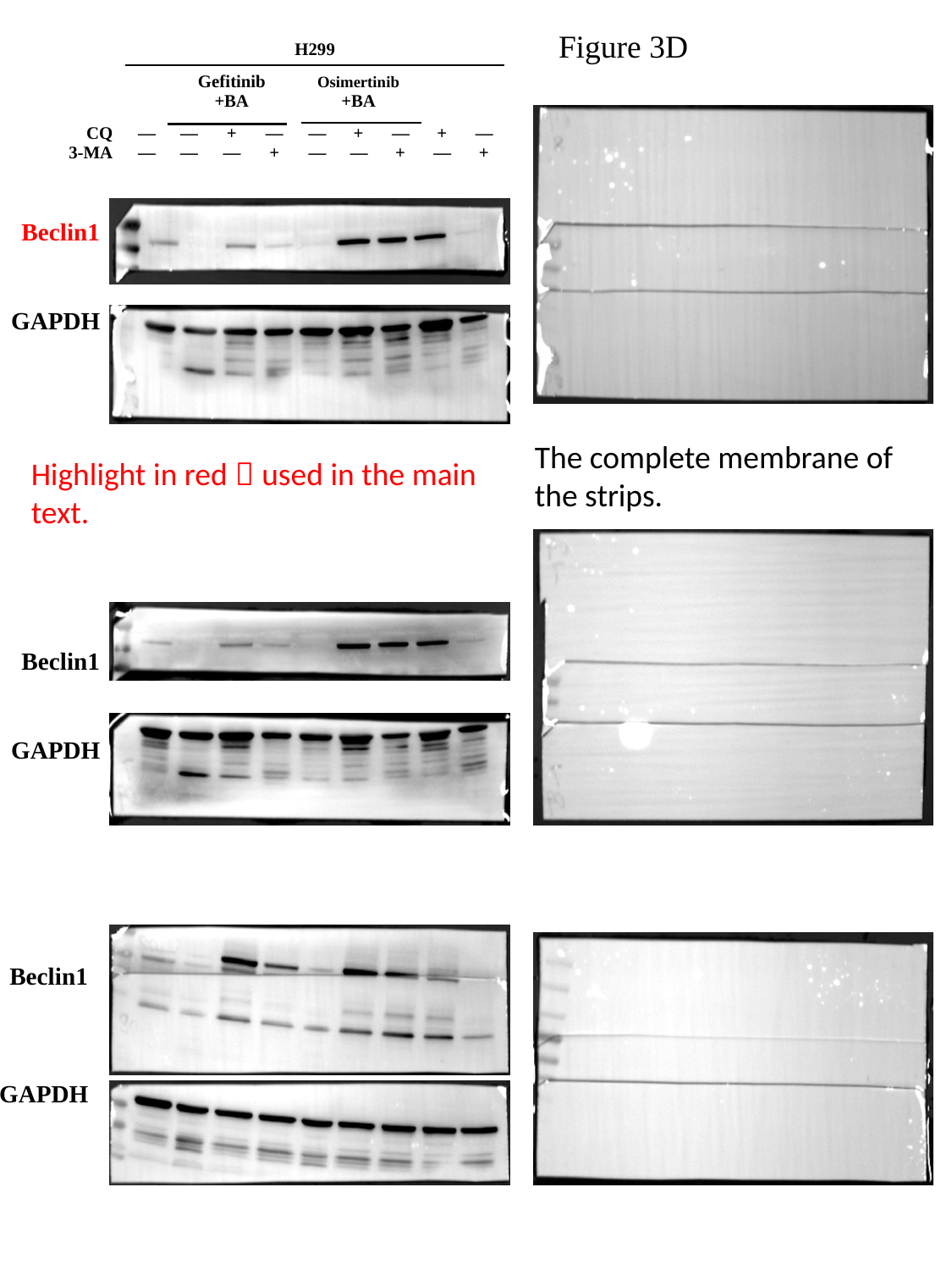

Figure 3D
| | H299 | | | | | | | | |
| --- | --- | --- | --- | --- | --- | --- | --- | --- | --- |
| | | Gefitinib +BA | | | Osimertinib +BA | | | | |
| CQ 3-MA | —— | —— | + — | — + | —— | + — | — + | + — | — + |
Beclin1
GAPDH
The complete membrane of the strips.
Highlight in red：used in the main text.
Beclin1
GAPDH
Beclin1
GAPDH

## Slide 15
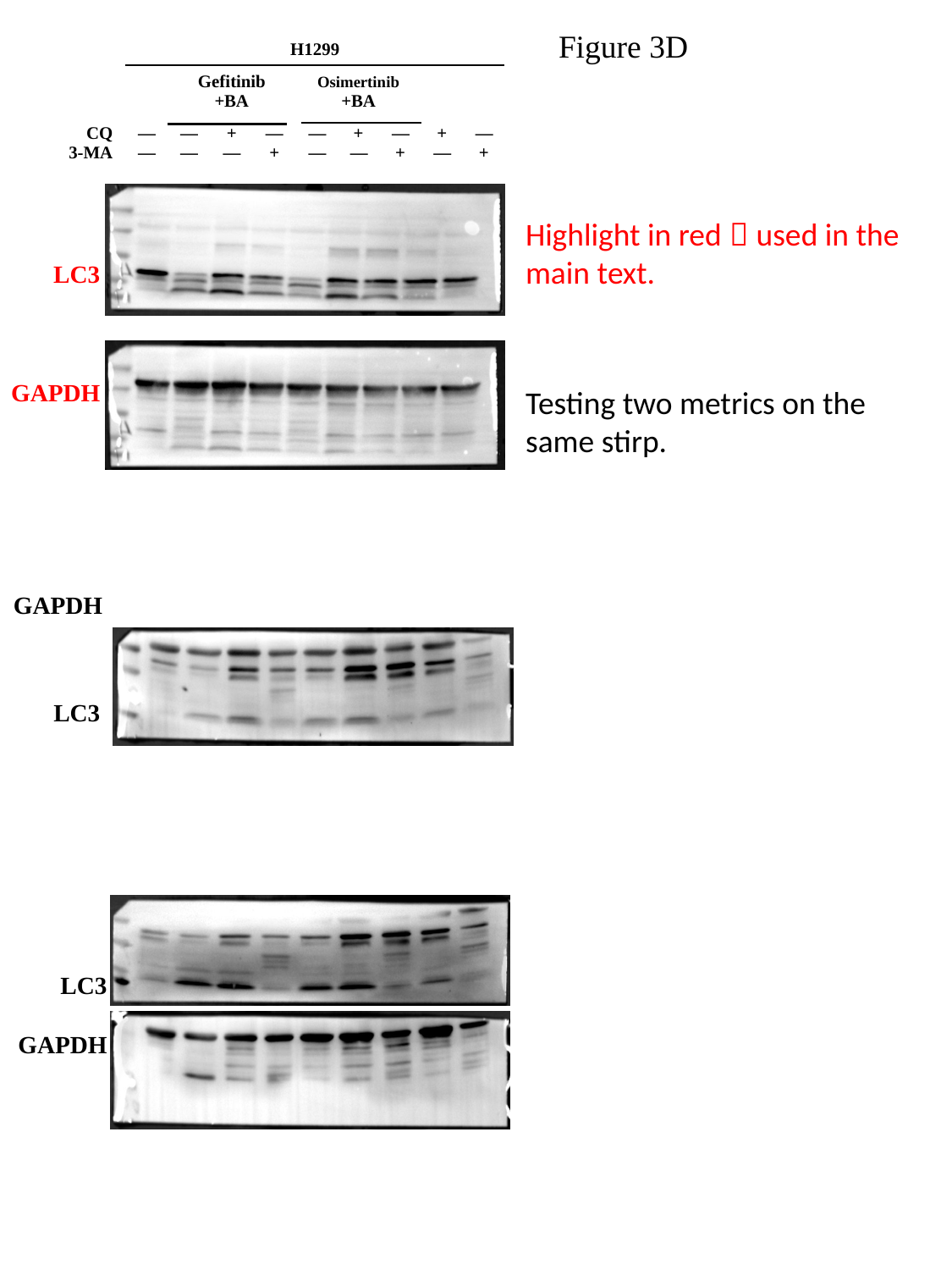

Figure 3D
| | H1299 | | | | | | | | |
| --- | --- | --- | --- | --- | --- | --- | --- | --- | --- |
| | | Gefitinib +BA | | | Osimertinib +BA | | | | |
| CQ 3-MA | —— | —— | + — | — + | —— | + — | — + | + — | — + |
Highlight in red：used in the main text.
LC3
GAPDH
Testing two metrics on the same stirp.
GAPDH
LC3
LC3
GAPDH

## Slide 16
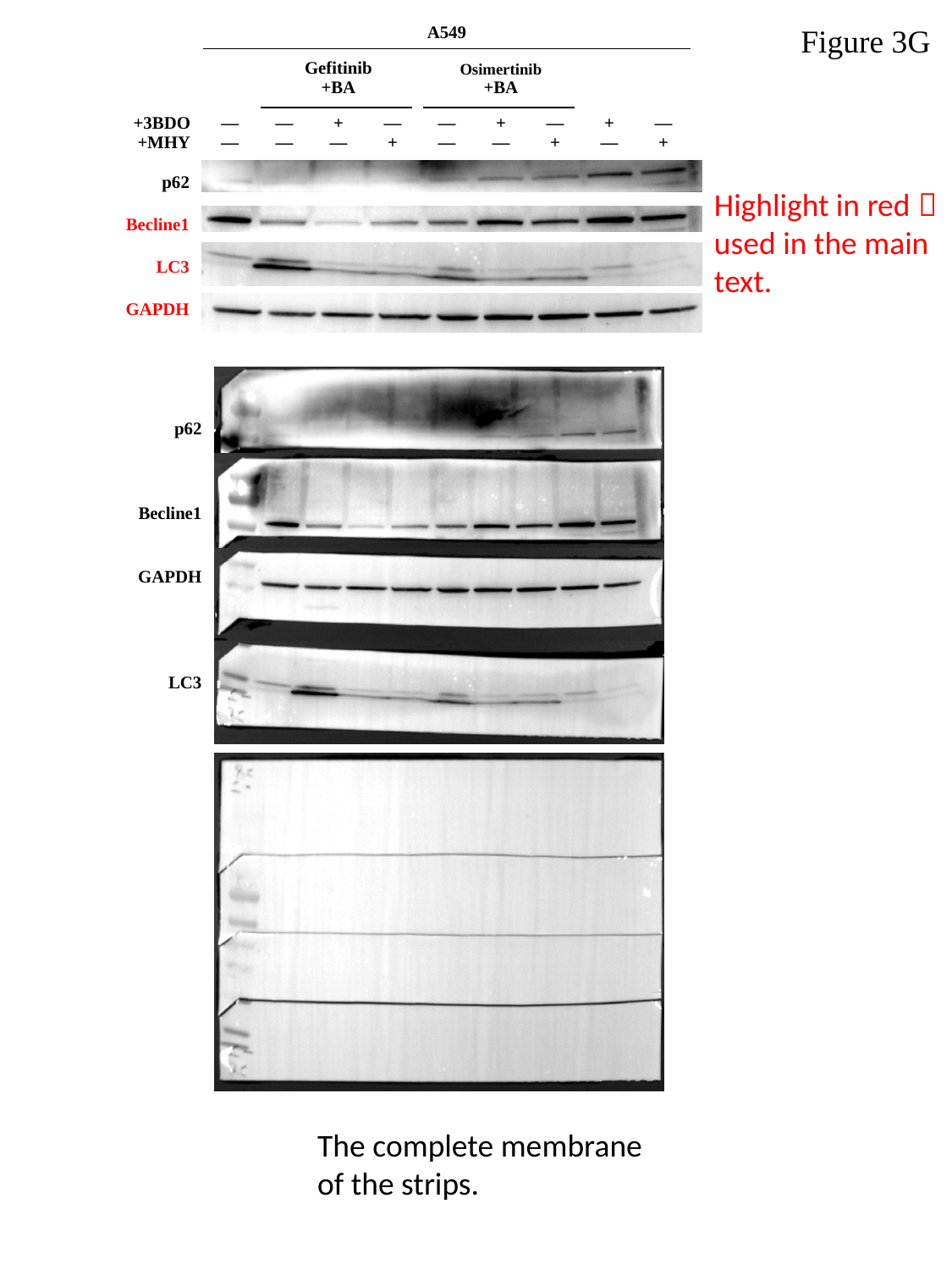

| | A549 | | | | | | | | |
| --- | --- | --- | --- | --- | --- | --- | --- | --- | --- |
| | | Gefitinib +BA | | | Osimertinib +BA | | | | |
| +3BDO +MHY | —— | —— | + — | — + | —— | + — | — + | + — | — + |
Figure 3G
p62
Becline1
LC3
GAPDH
Highlight in red：used in the main text.
p62
Becline1
GAPDH
LC3
The complete membrane of the strips.

## Slide 17
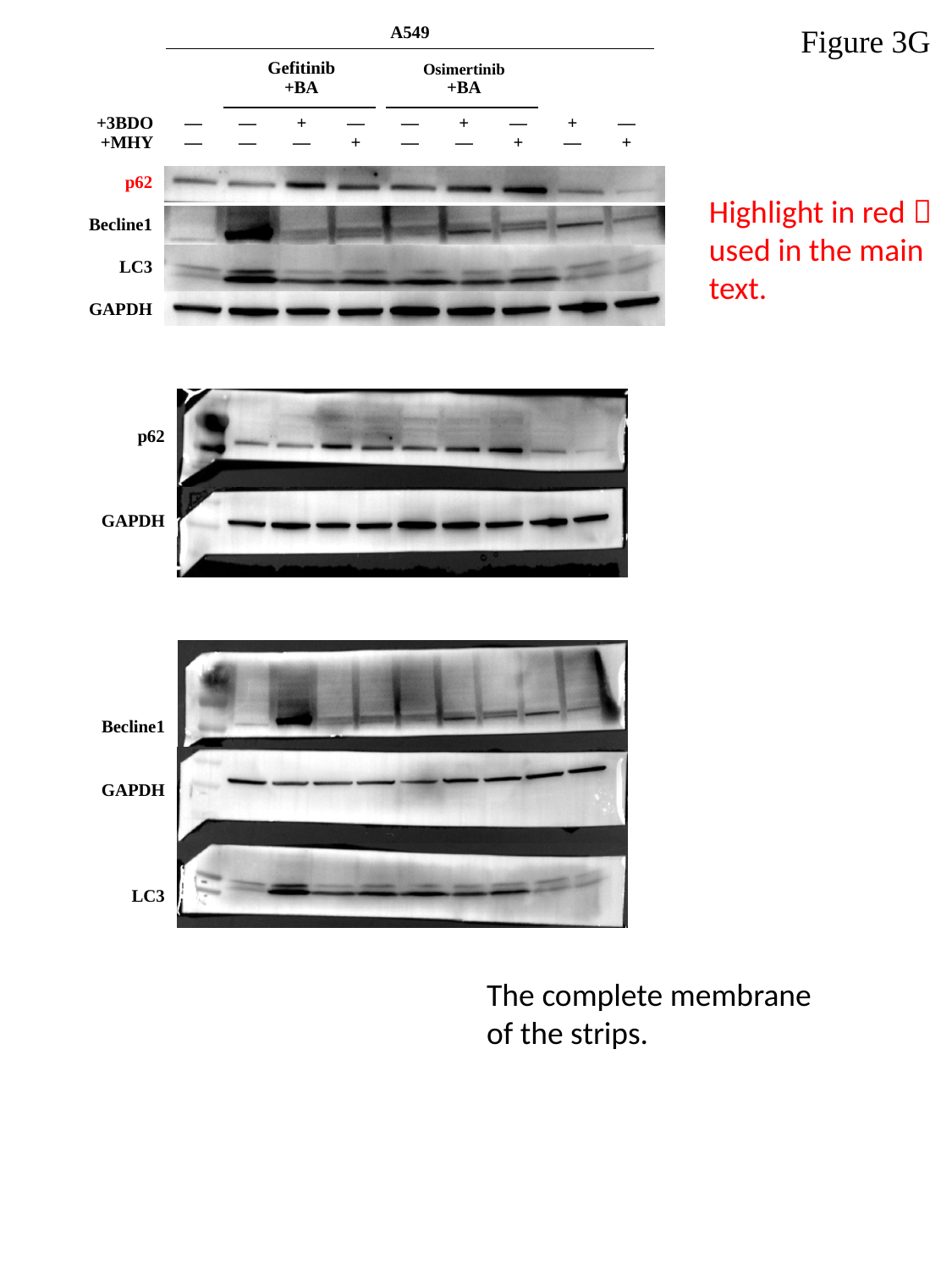

| | A549 | | | | | | | | |
| --- | --- | --- | --- | --- | --- | --- | --- | --- | --- |
| | | Gefitinib +BA | | | Osimertinib +BA | | | | |
| +3BDO +MHY | —— | —— | + — | — + | —— | + — | — + | + — | — + |
Figure 3G
p62
Becline1
LC3
GAPDH
Highlight in red：used in the main text.
p62
GAPDH
Becline1
GAPDH
LC3
The complete membrane of the strips.

## Slide 18
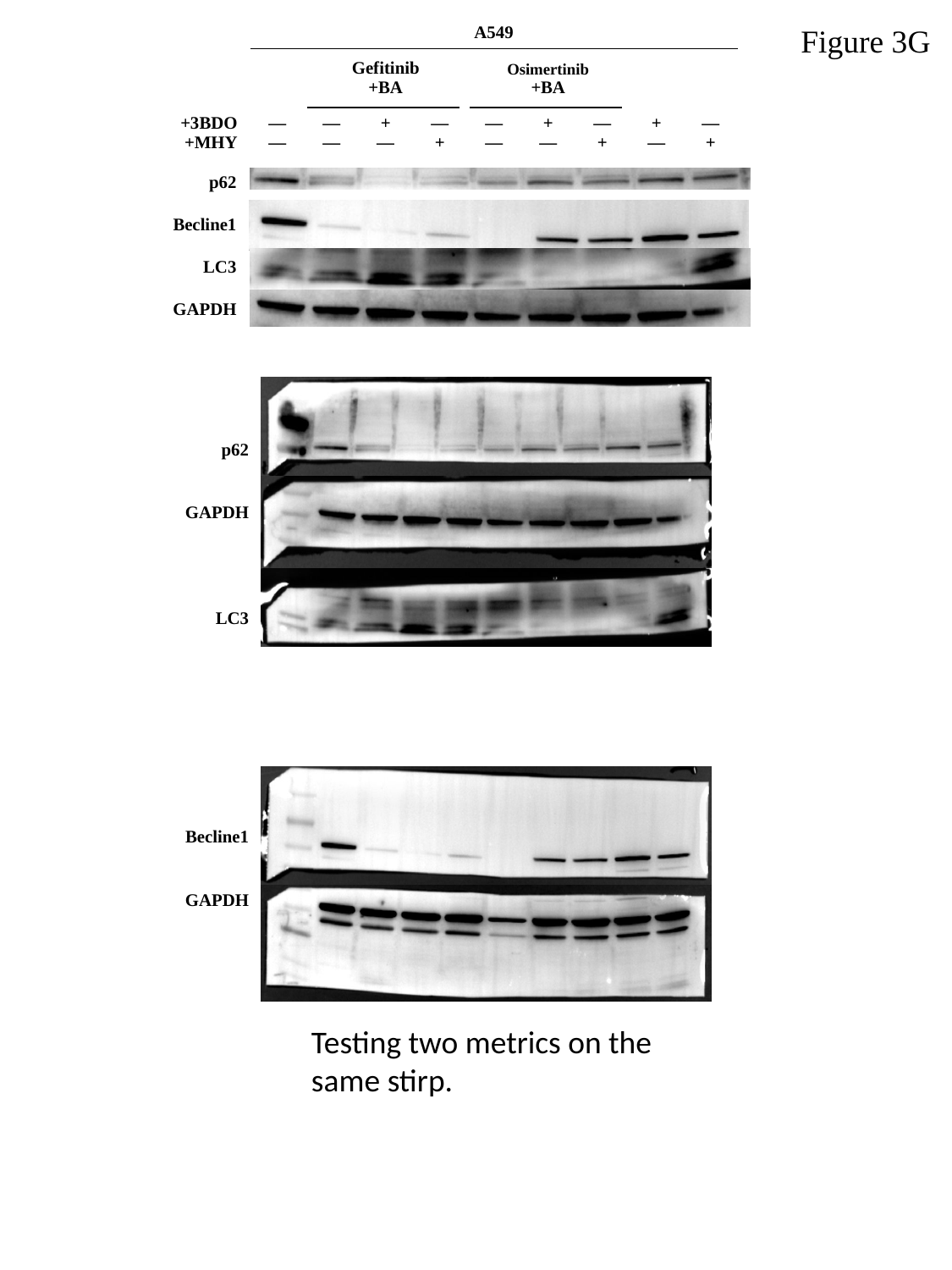

| | A549 | | | | | | | | |
| --- | --- | --- | --- | --- | --- | --- | --- | --- | --- |
| | | Gefitinib +BA | | | Osimertinib +BA | | | | |
| +3BDO +MHY | —— | —— | + — | — + | —— | + — | — + | + — | — + |
Figure 3G
p62
Becline1
LC3
GAPDH
p62
GAPDH
LC3
Becline1
GAPDH
Testing two metrics on the same stirp.

## Slide 19
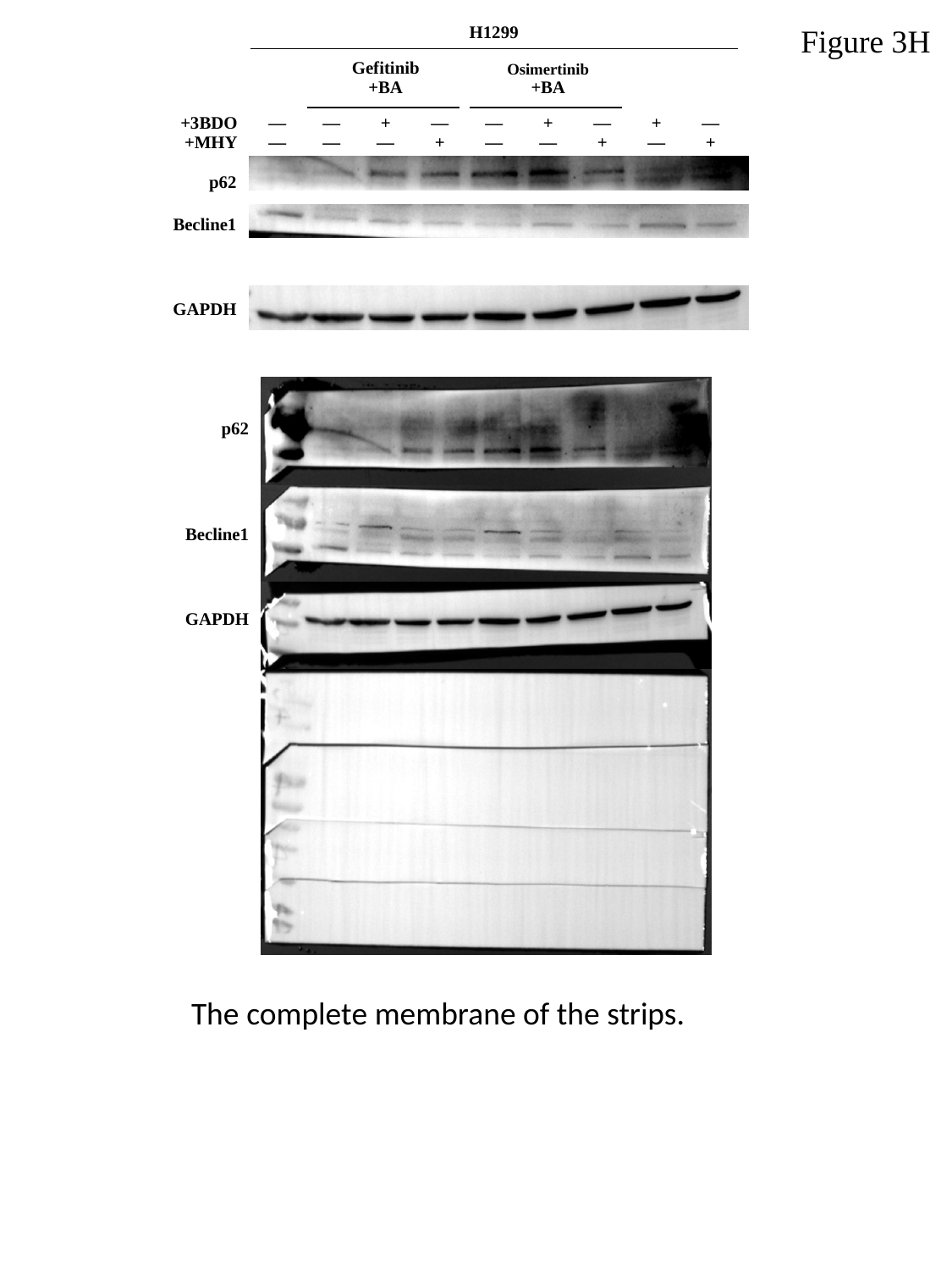

| | H1299 | | | | | | | | |
| --- | --- | --- | --- | --- | --- | --- | --- | --- | --- |
| | | Gefitinib +BA | | | Osimertinib +BA | | | | |
| +3BDO +MHY | —— | —— | + — | — + | —— | + — | — + | + — | — + |
Figure 3H
p62
Becline1
GAPDH
p62
Becline1
GAPDH
The complete membrane of the strips.

## Slide 20
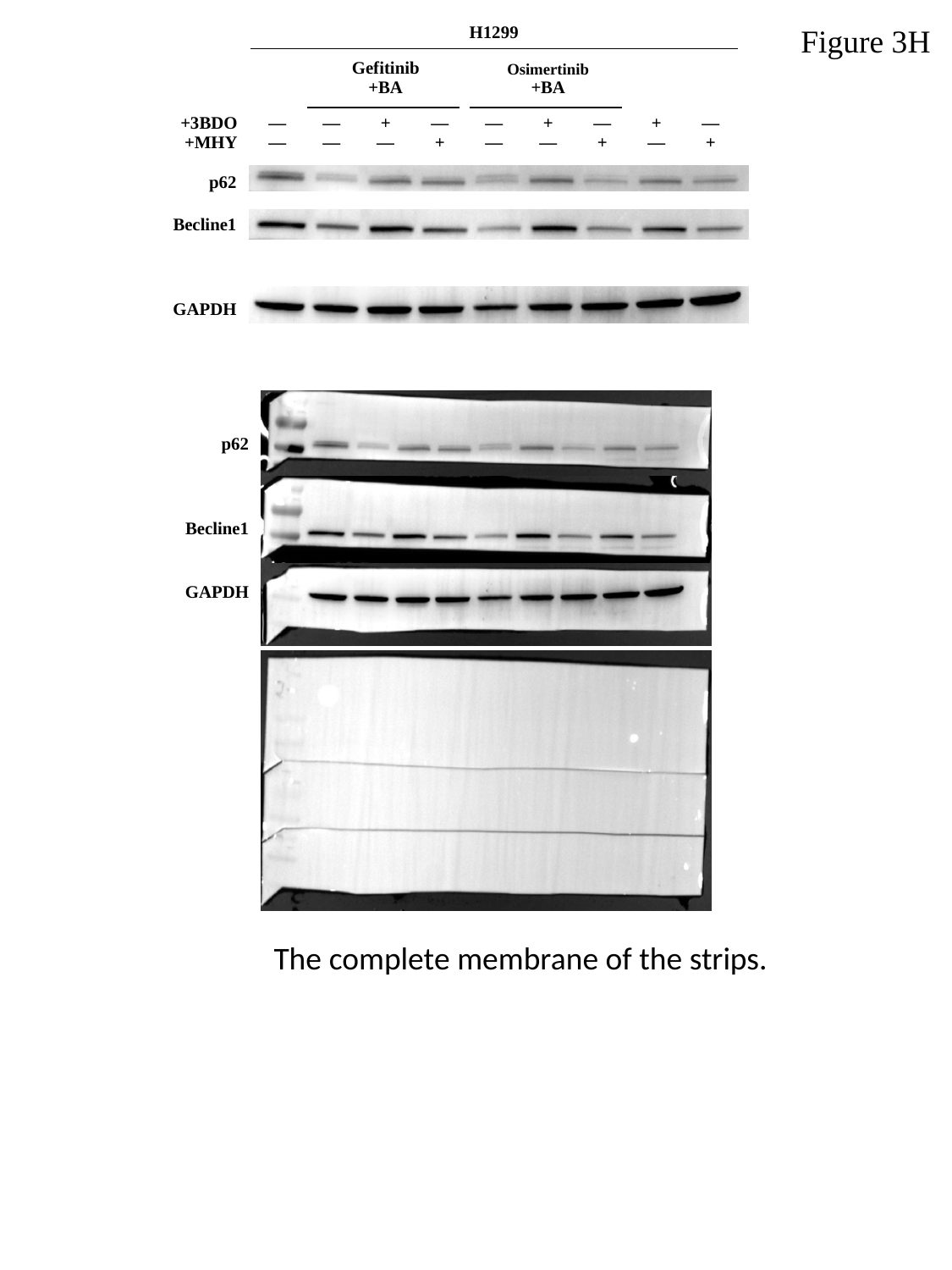

| | H1299 | | | | | | | | |
| --- | --- | --- | --- | --- | --- | --- | --- | --- | --- |
| | | Gefitinib +BA | | | Osimertinib +BA | | | | |
| +3BDO +MHY | —— | —— | + — | — + | —— | + — | — + | + — | — + |
Figure 3H
p62
Becline1
GAPDH
p62
Becline1
GAPDH
The complete membrane of the strips.

## Slide 21
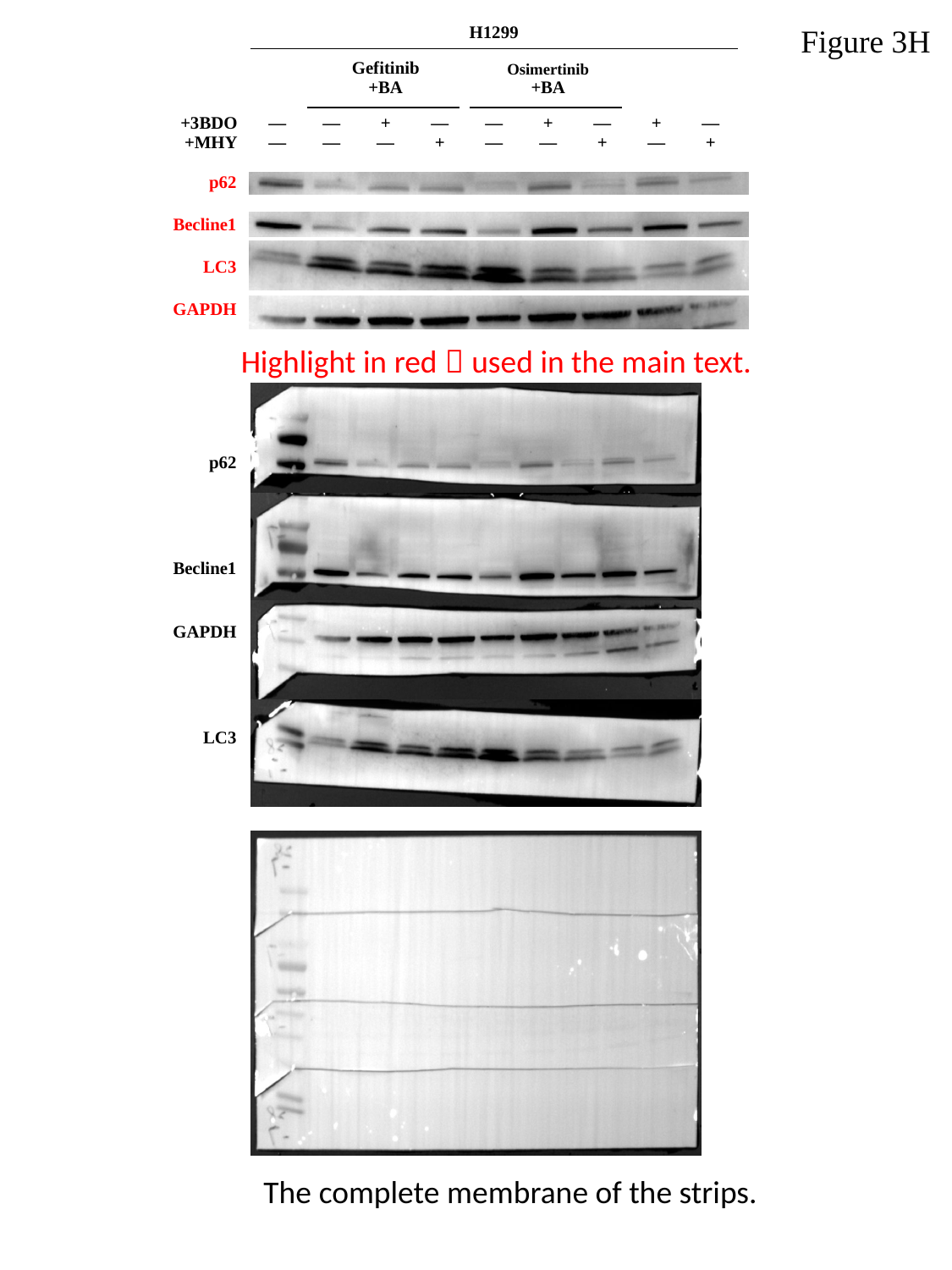

| | H1299 | | | | | | | | |
| --- | --- | --- | --- | --- | --- | --- | --- | --- | --- |
| | | Gefitinib +BA | | | Osimertinib +BA | | | | |
| +3BDO +MHY | —— | —— | + — | — + | —— | + — | — + | + — | — + |
Figure 3H
p62
Becline1
LC3
GAPDH
Highlight in red：used in the main text.
p62
Becline1
GAPDH
LC3
The complete membrane of the strips.

## Slide 22
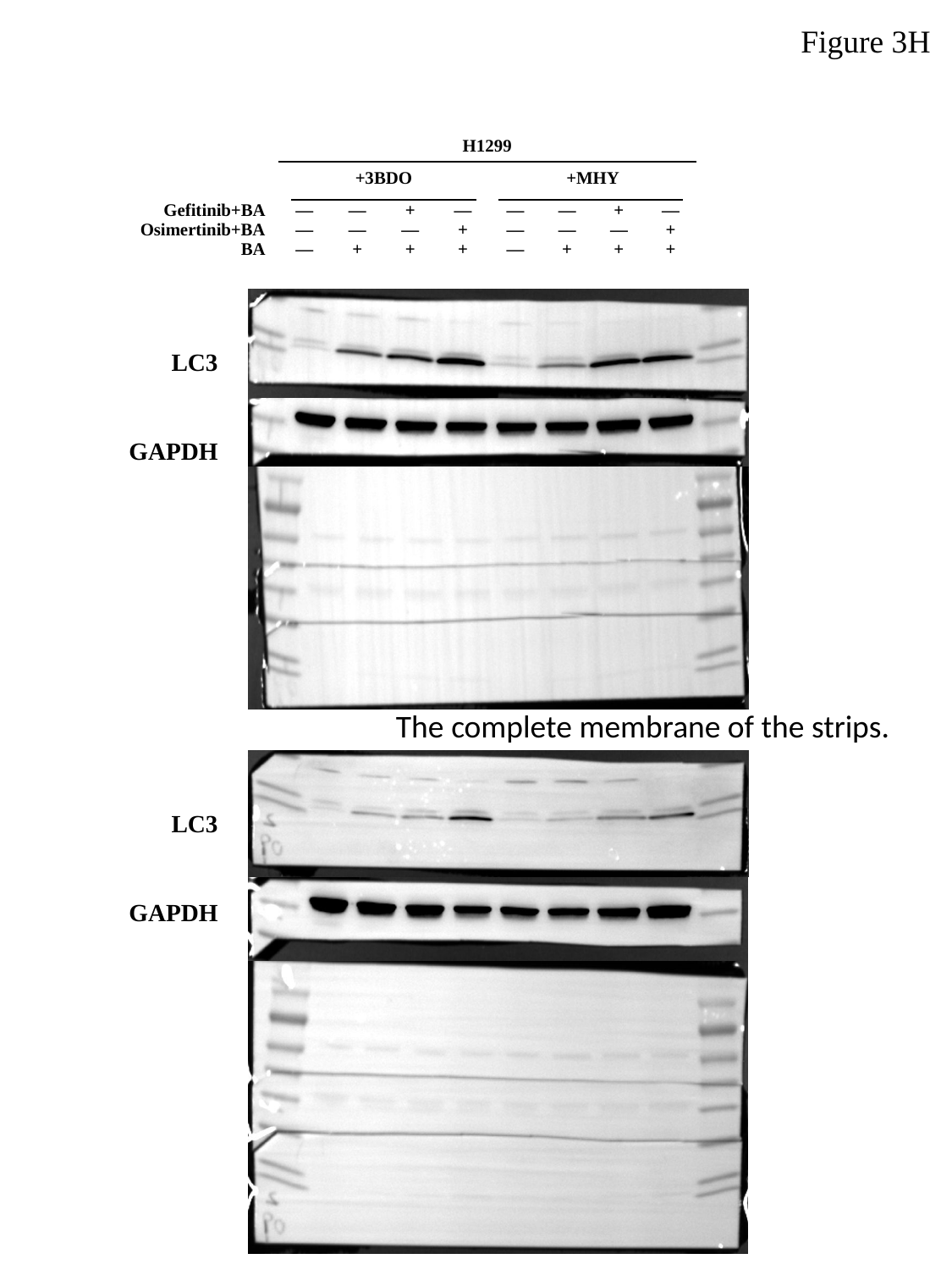

Figure 3H
| | H1299 | | | | | | | |
| --- | --- | --- | --- | --- | --- | --- | --- | --- |
| | +3BDO | | | | +MHY | | | |
| Gefitinib+BA Osimertinib+BA BA | ——— | —— + | + —+ | — + + | — — — | —— + | + —+ | — + + |
LC3
GAPDH
The complete membrane of the strips.
LC3
GAPDH

## Slide 23
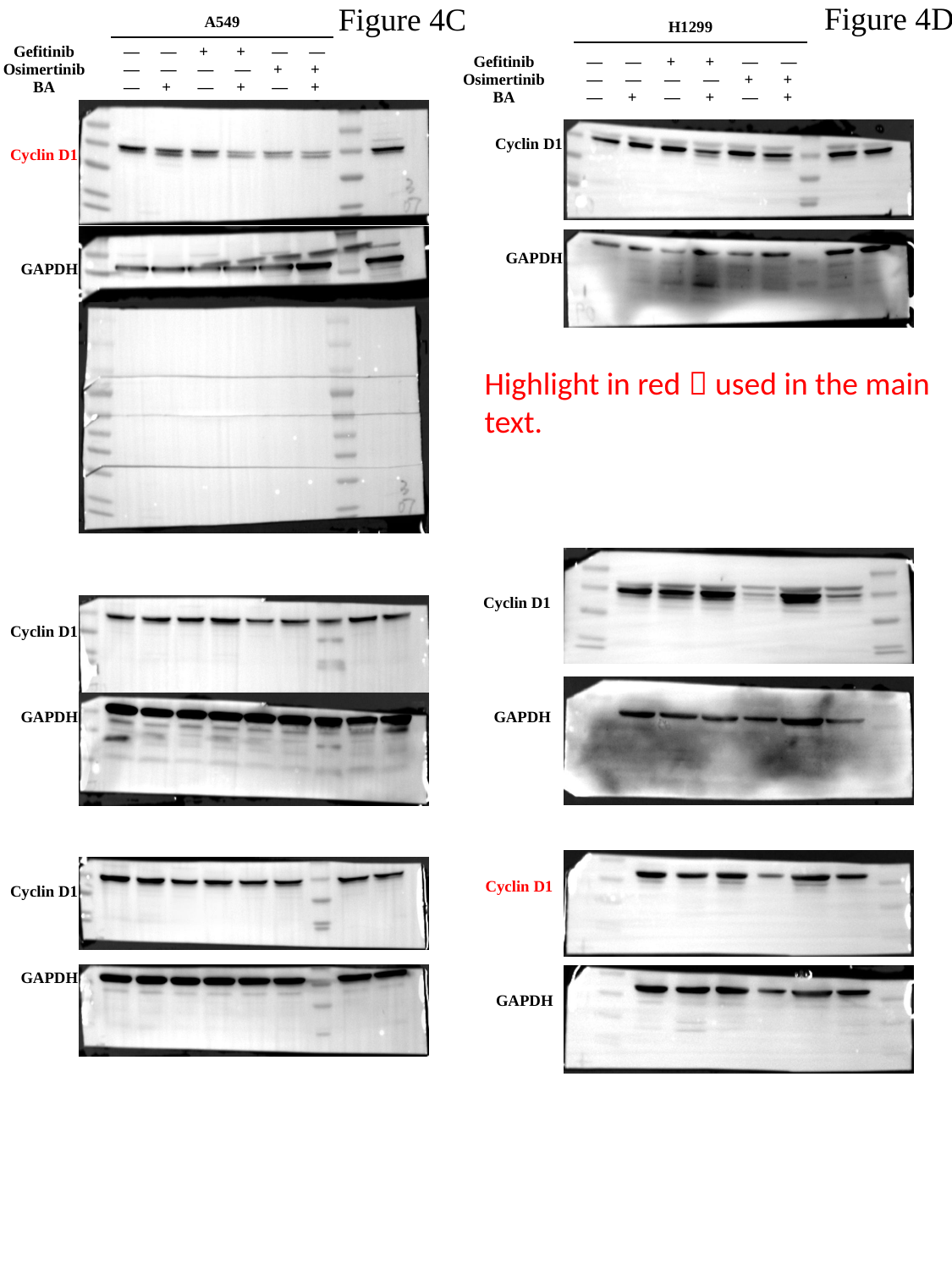

| | H1299 | | | | | |
| --- | --- | --- | --- | --- | --- | --- |
| Gefitinib Osimertinib BA | ——— | —— + | + —— | + — + | — + — | — + + |
Figure 4D
Figure 4C
| | A549 | | | | | |
| --- | --- | --- | --- | --- | --- | --- |
| Gefitinib Osimertinib BA | ——— | —— + | + —— | + — + | — + — | — + + |
Cyclin D1
GAPDH
Cyclin D1
GAPDH
Highlight in red：used in the main text.
Cyclin D1
GAPDH
Cyclin D1
GAPDH
Cyclin D1
GAPDH
Cyclin D1
GAPDH

## Slide 24
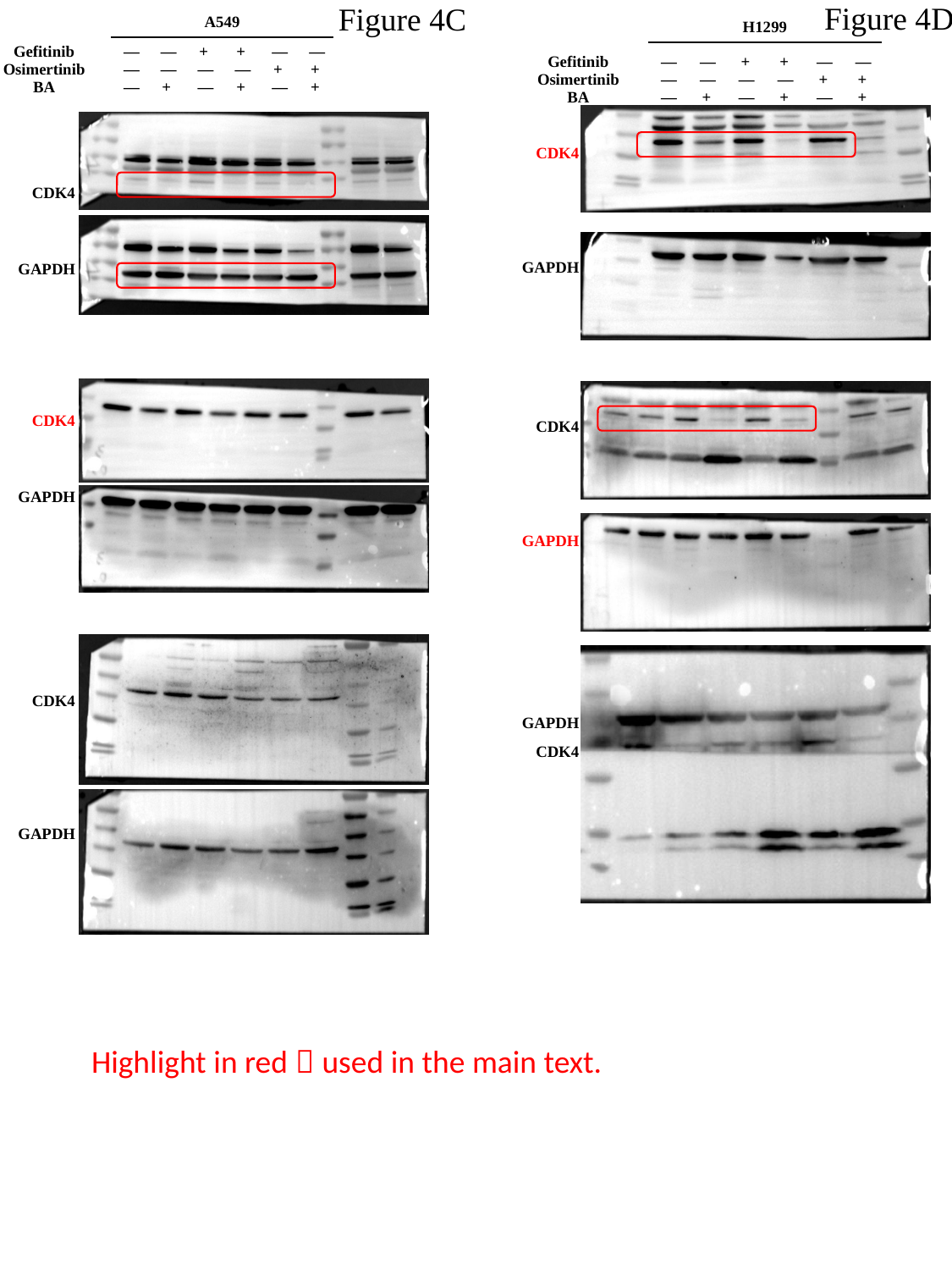

| | H1299 | | | | | |
| --- | --- | --- | --- | --- | --- | --- |
| Gefitinib Osimertinib BA | ——— | —— + | + —— | + — + | — + — | — + + |
Figure 4D
Figure 4C
| | A549 | | | | | |
| --- | --- | --- | --- | --- | --- | --- |
| Gefitinib Osimertinib BA | ——— | —— + | + —— | + — + | — + — | — + + |
CDK4
GAPDH
CDK4
GAPDH
CDK4
GAPDH
CDK4
GAPDH
CDK4
GAPDH
GAPDH
CDK4
Highlight in red：used in the main text.

## Slide 25
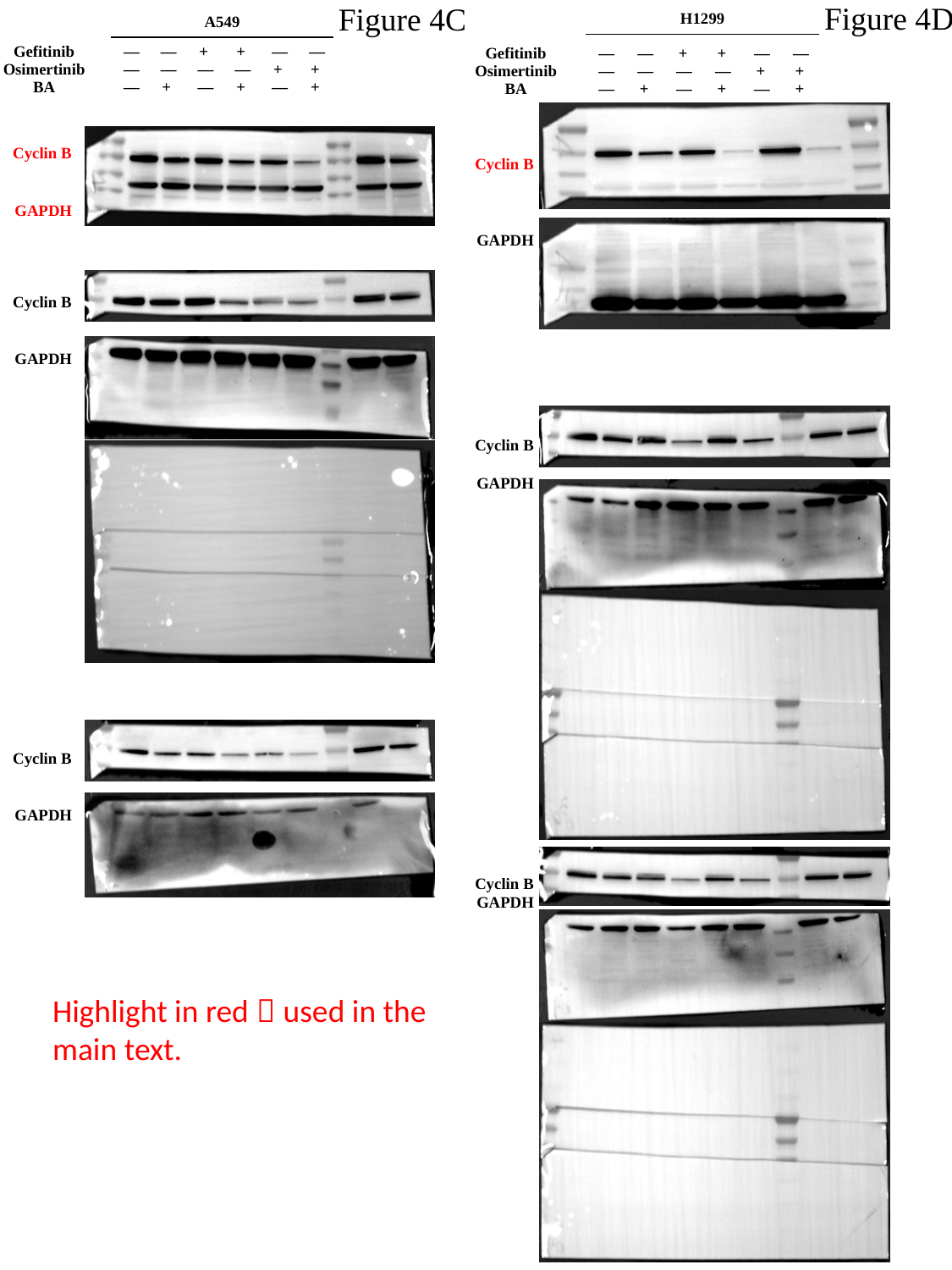

| | H1299 | | | | | |
| --- | --- | --- | --- | --- | --- | --- |
| Gefitinib Osimertinib BA | ——— | —— + | + —— | + — + | — + — | — + + |
Figure 4D
Figure 4C
| | A549 | | | | | |
| --- | --- | --- | --- | --- | --- | --- |
| Gefitinib Osimertinib BA | ——— | —— + | + —— | + — + | — + — | — + + |
Cyclin B
GAPDH
Cyclin B
GAPDH
Cyclin B
GAPDH
Cyclin B
GAPDH
Cyclin B
GAPDH
Cyclin B
GAPDH
Highlight in red：used in the main text.

## Slide 26
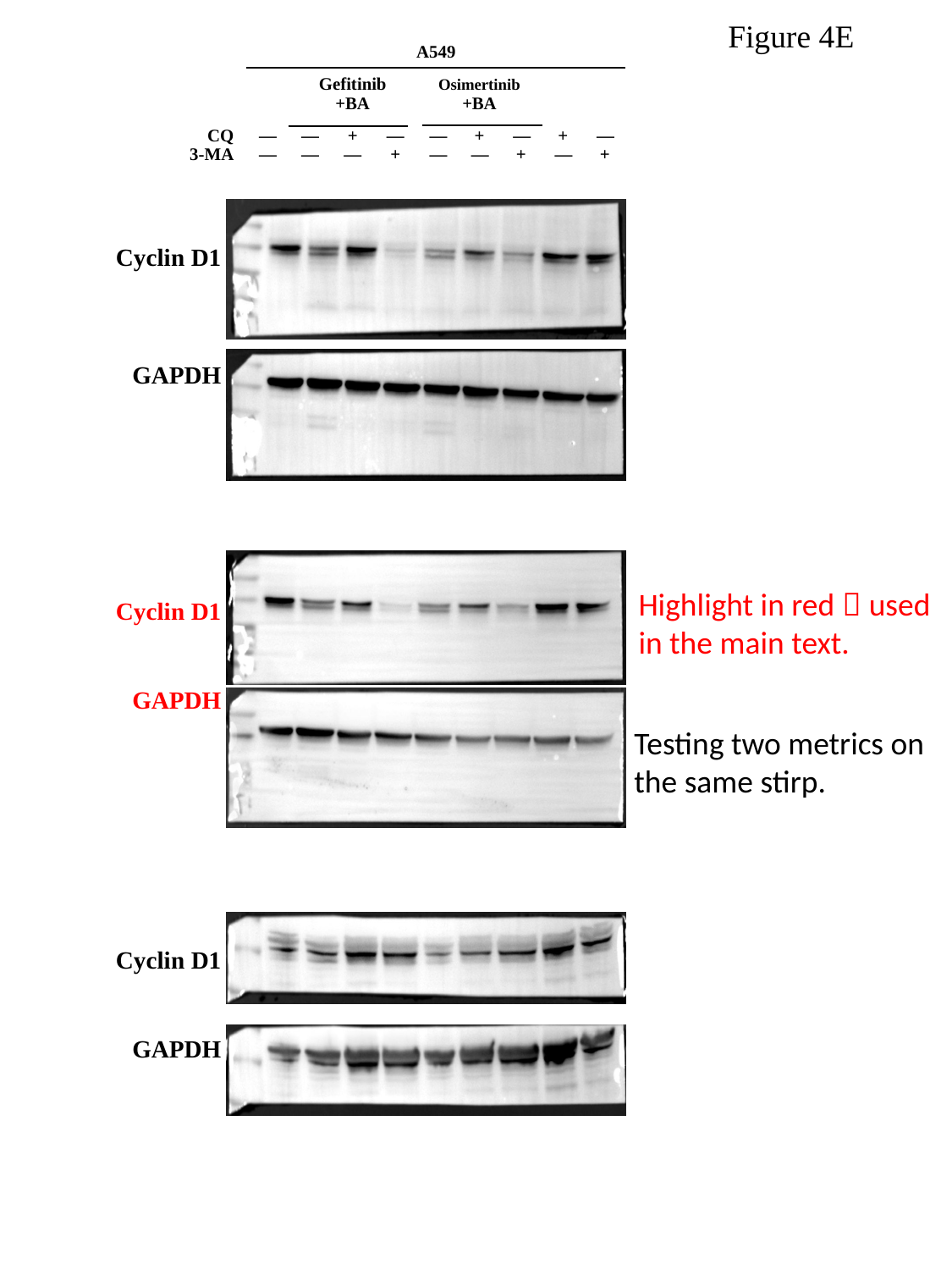

Figure 4E
| | A549 | | | | | | | | |
| --- | --- | --- | --- | --- | --- | --- | --- | --- | --- |
| | | Gefitinib +BA | | | Osimertinib +BA | | | | |
| CQ 3-MA | —— | —— | + — | — + | —— | + — | — + | + — | — + |
Cyclin D1
GAPDH
Highlight in red：used in the main text.
Cyclin D1
GAPDH
Testing two metrics on the same stirp.
Cyclin D1
GAPDH

## Slide 27
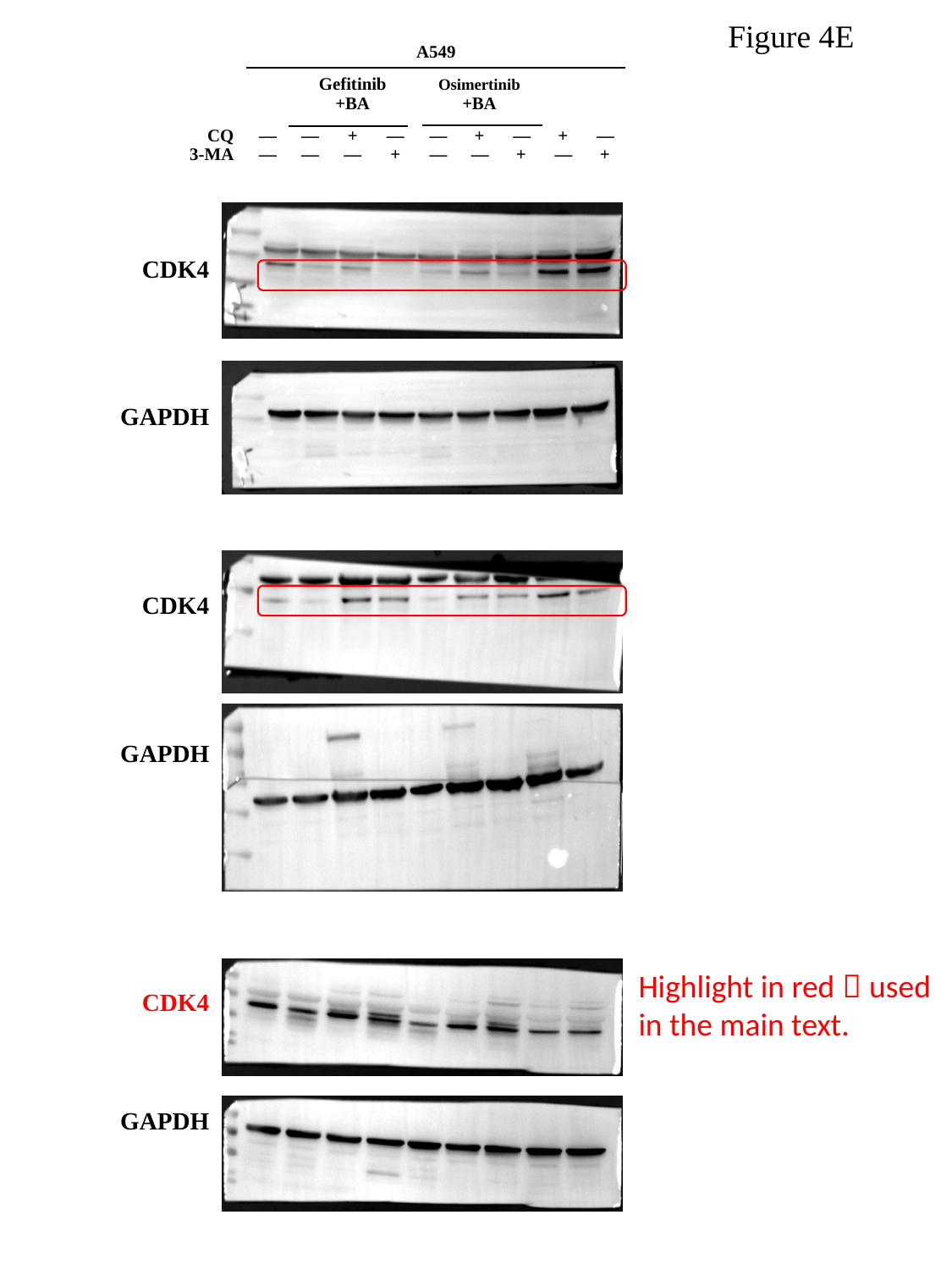

Figure 4E
| | A549 | | | | | | | | |
| --- | --- | --- | --- | --- | --- | --- | --- | --- | --- |
| | | Gefitinib +BA | | | Osimertinib +BA | | | | |
| CQ 3-MA | —— | —— | + — | — + | —— | + — | — + | + — | — + |
CDK4
GAPDH
CDK4
GAPDH
Highlight in red：used in the main text.
CDK4
GAPDH

## Slide 28
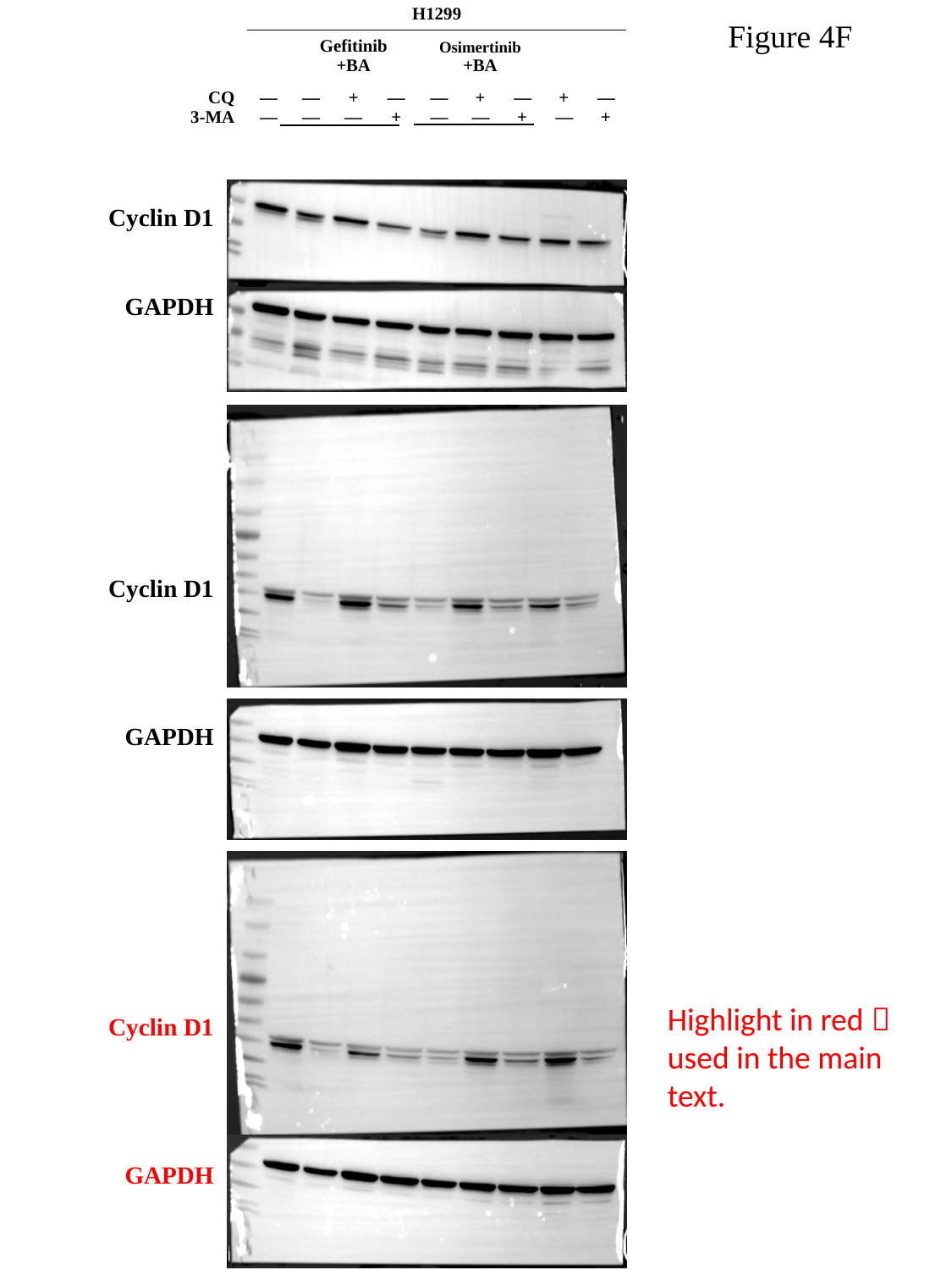

| | H1299 | | | | | | | | |
| --- | --- | --- | --- | --- | --- | --- | --- | --- | --- |
| | | Gefitinib +BA | | | Osimertinib +BA | | | | |
| CQ 3-MA | —— | —— | + — | — + | —— | + — | — + | + — | — + |
Figure 4F
Cyclin D1
GAPDH
Cyclin D1
GAPDH
Highlight in red：used in the main text.
Cyclin D1
GAPDH

## Slide 29
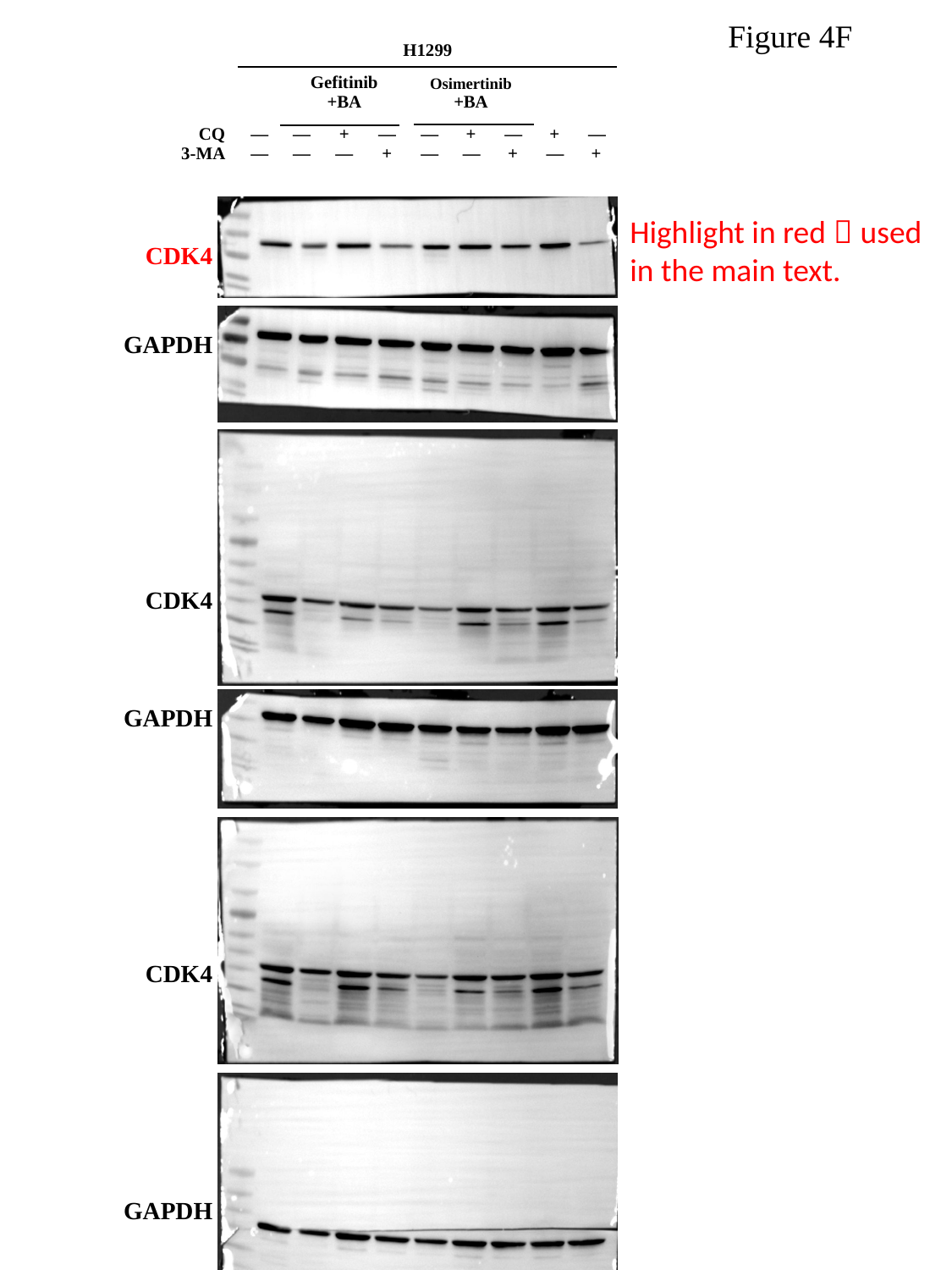

Figure 4F
| | H1299 | | | | | | | | |
| --- | --- | --- | --- | --- | --- | --- | --- | --- | --- |
| | | Gefitinib +BA | | | Osimertinib +BA | | | | |
| CQ 3-MA | —— | —— | + — | — + | —— | + — | — + | + — | — + |
Highlight in red：used in the main text.
CDK4
GAPDH
CDK4
GAPDH
CDK4
GAPDH

## Slide 30
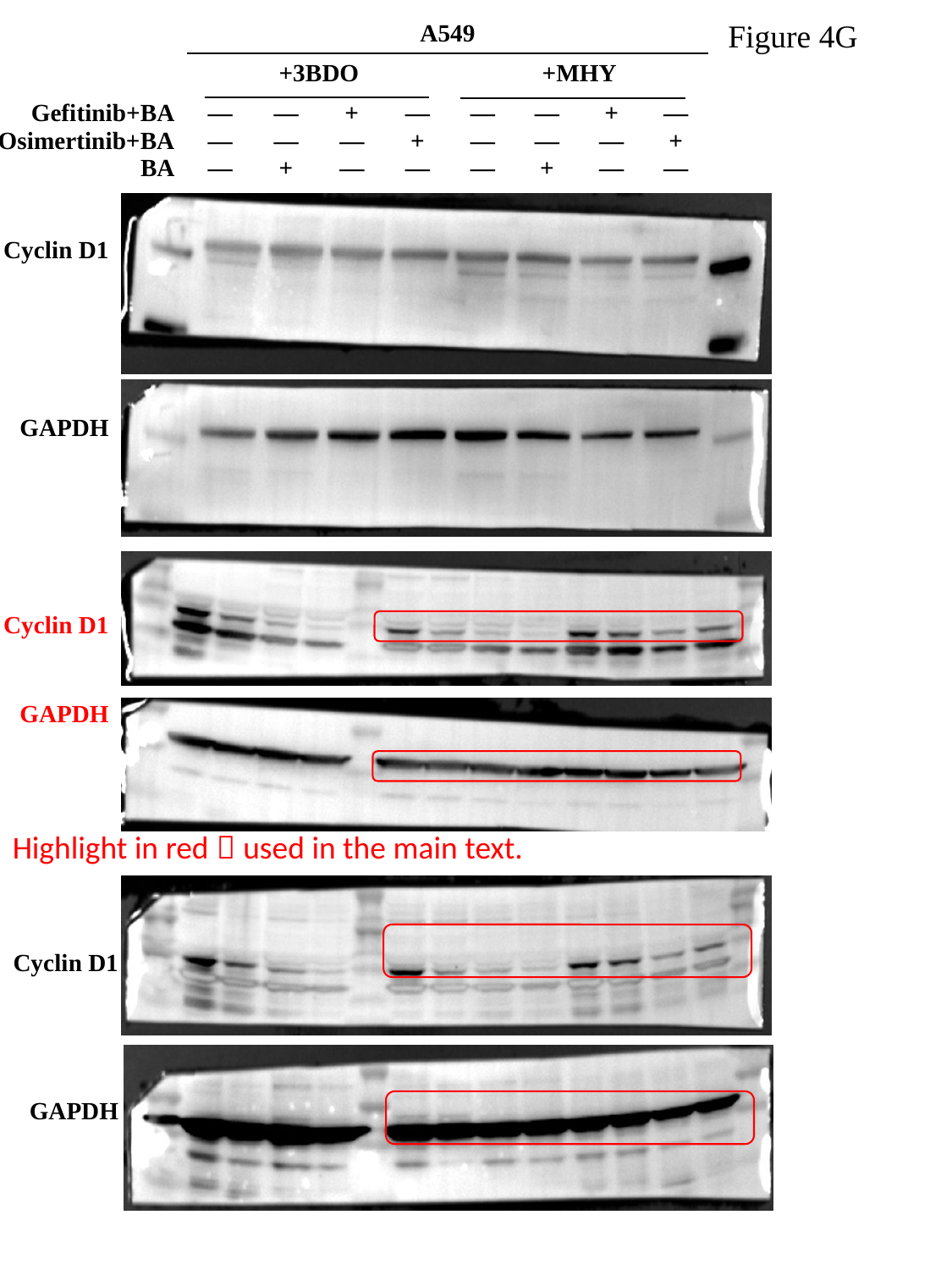

Figure 4G
| | A549 | | | | | | | |
| --- | --- | --- | --- | --- | --- | --- | --- | --- |
| | +3BDO | | | | +MHY | | | |
| Gefitinib+BA Osimertinib+BA BA | ——— | —— + | + —— | — + — | — — — | —— + | + —— | — + — |
Cyclin D1
GAPDH
Cyclin D1
GAPDH
Highlight in red：used in the main text.
Cyclin D1
GAPDH

## Slide 31
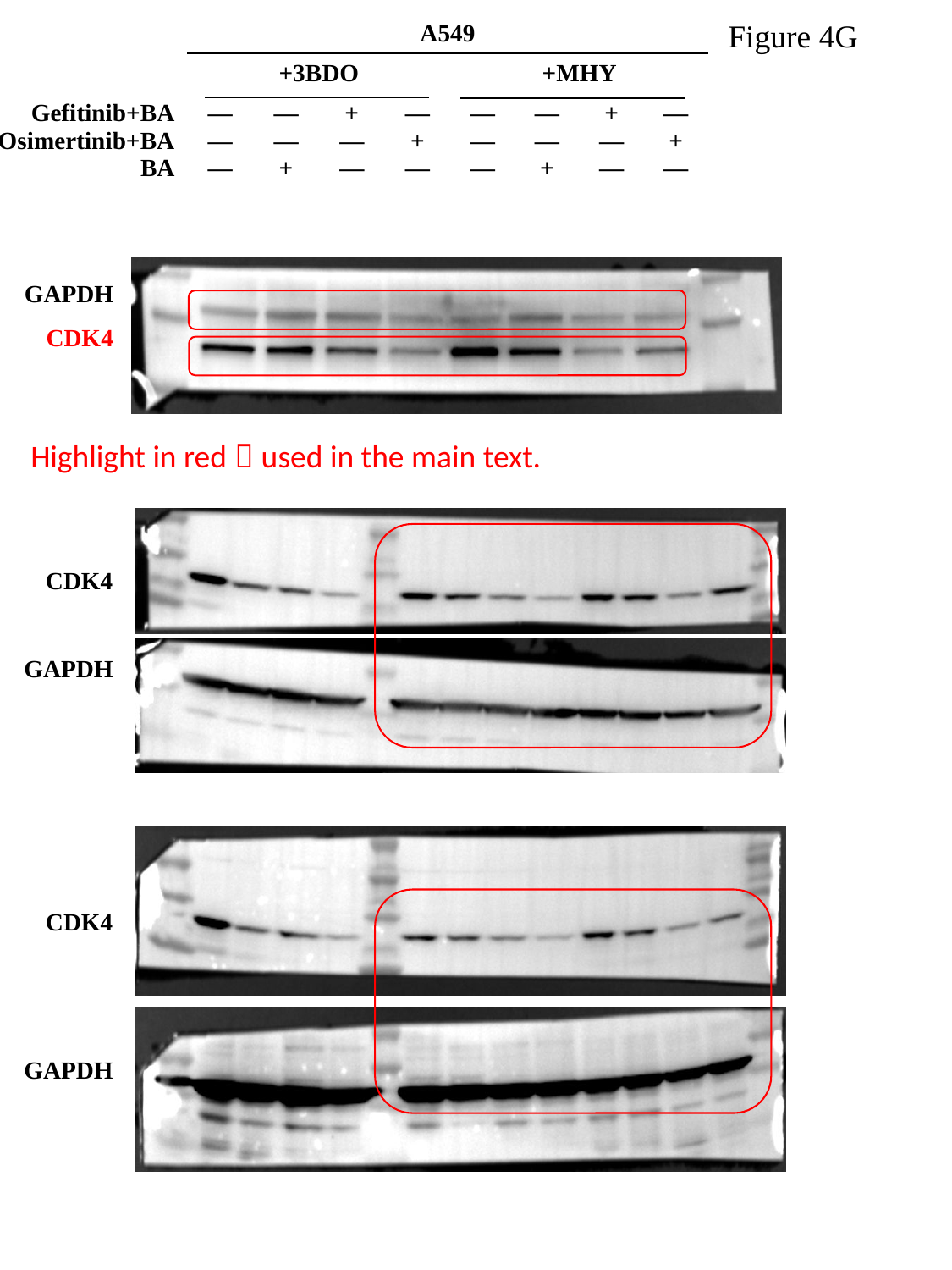

Figure 4G
| | A549 | | | | | | | |
| --- | --- | --- | --- | --- | --- | --- | --- | --- |
| | +3BDO | | | | +MHY | | | |
| Gefitinib+BA Osimertinib+BA BA | ——— | —— + | + —— | — + — | — — — | —— + | + —— | — + — |
GAPDH
CDK4
Highlight in red：used in the main text.
CDK4
GAPDH
CDK4
GAPDH

## Slide 32
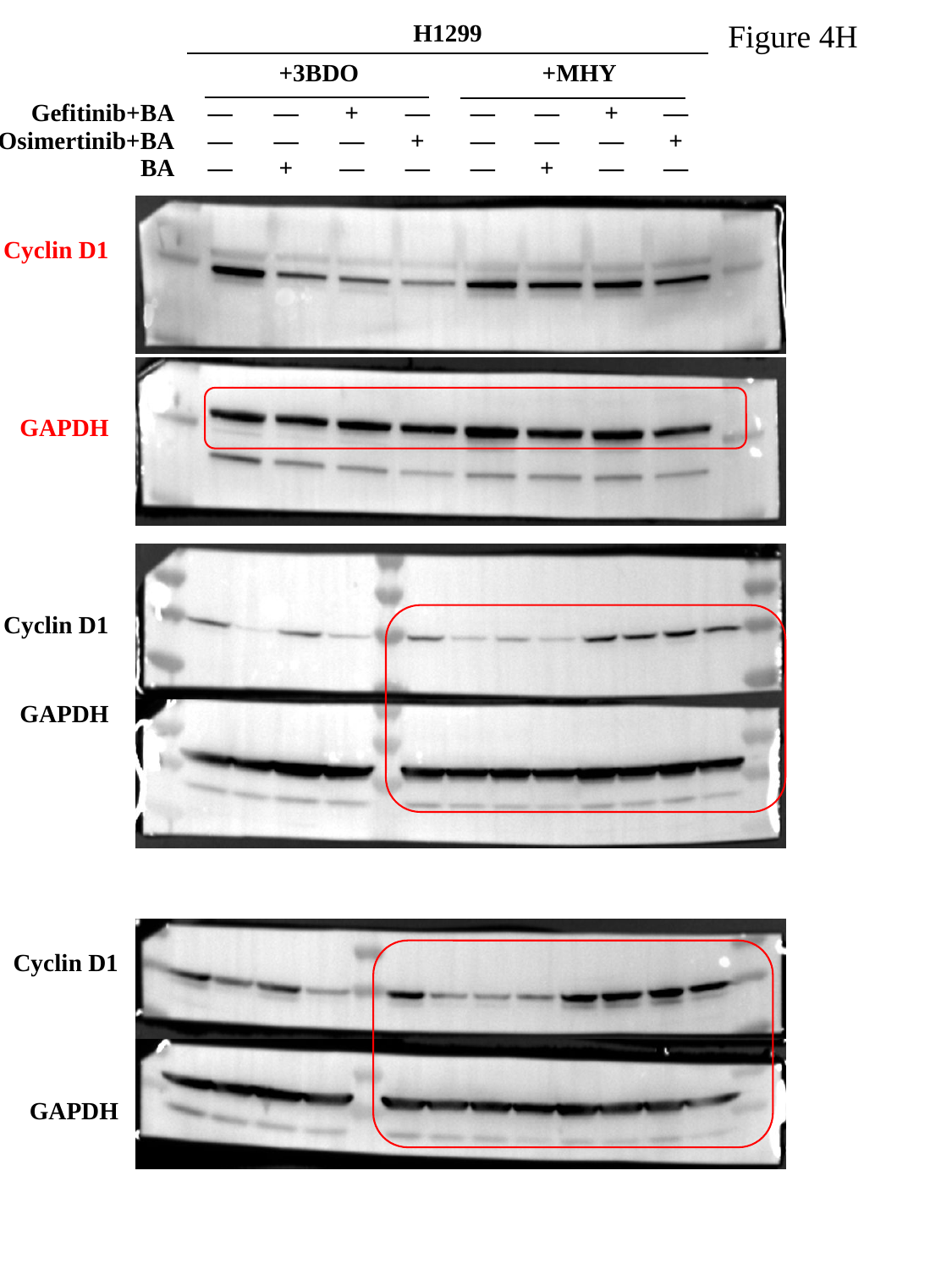

Figure 4H
| | H1299 | | | | | | | |
| --- | --- | --- | --- | --- | --- | --- | --- | --- |
| | +3BDO | | | | +MHY | | | |
| Gefitinib+BA Osimertinib+BA BA | ——— | —— + | + —— | — + — | — — — | —— + | + —— | — + — |
Cyclin D1
GAPDH
Cyclin D1
GAPDH
Cyclin D1
GAPDH

## Slide 33
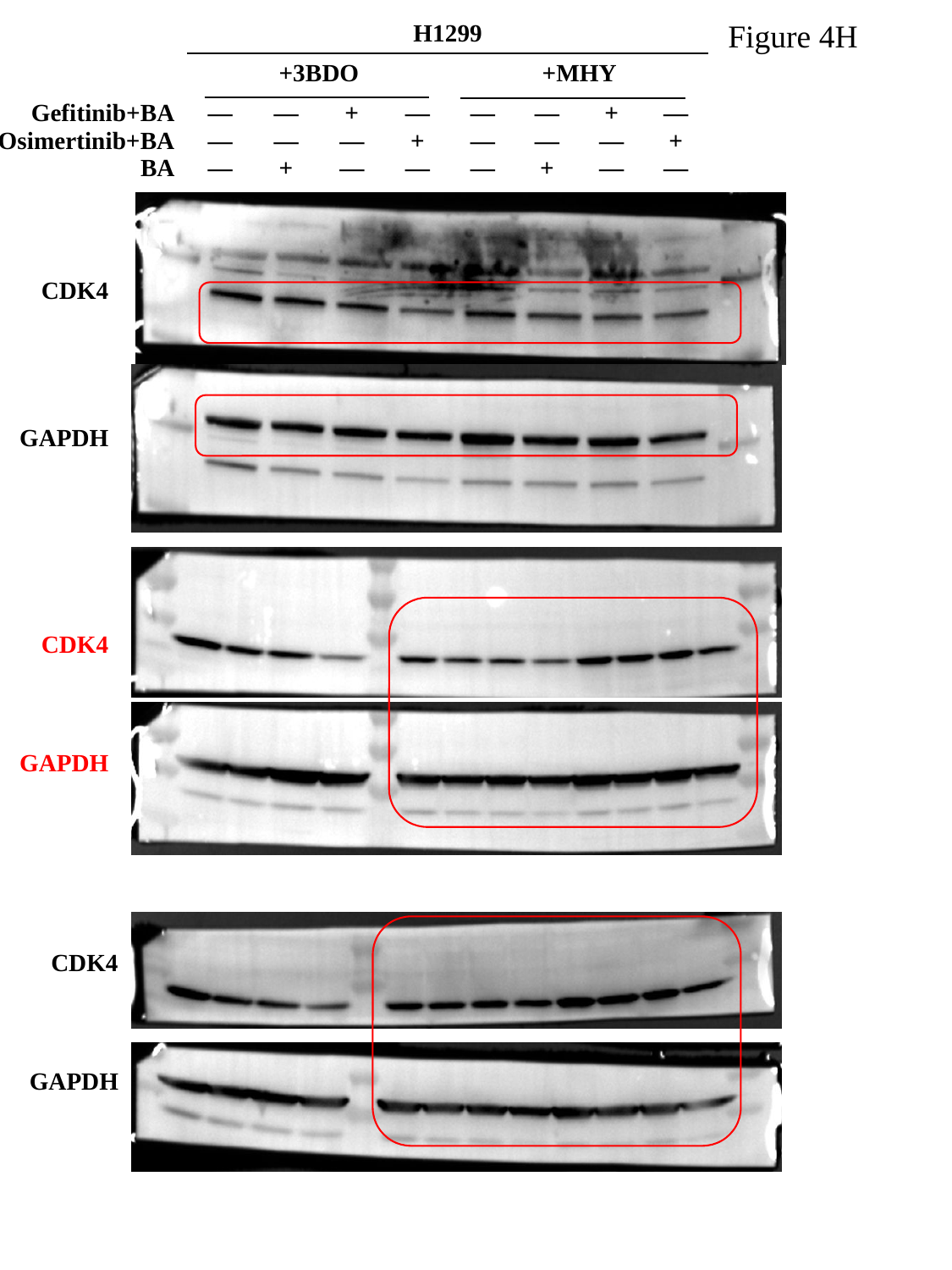

Figure 4H
| | H1299 | | | | | | | |
| --- | --- | --- | --- | --- | --- | --- | --- | --- |
| | +3BDO | | | | +MHY | | | |
| Gefitinib+BA Osimertinib+BA BA | ——— | —— + | + —— | — + — | — — — | —— + | + —— | — + — |
CDK4
GAPDH
CDK4
GAPDH
CDK4
GAPDH

## Slide 34
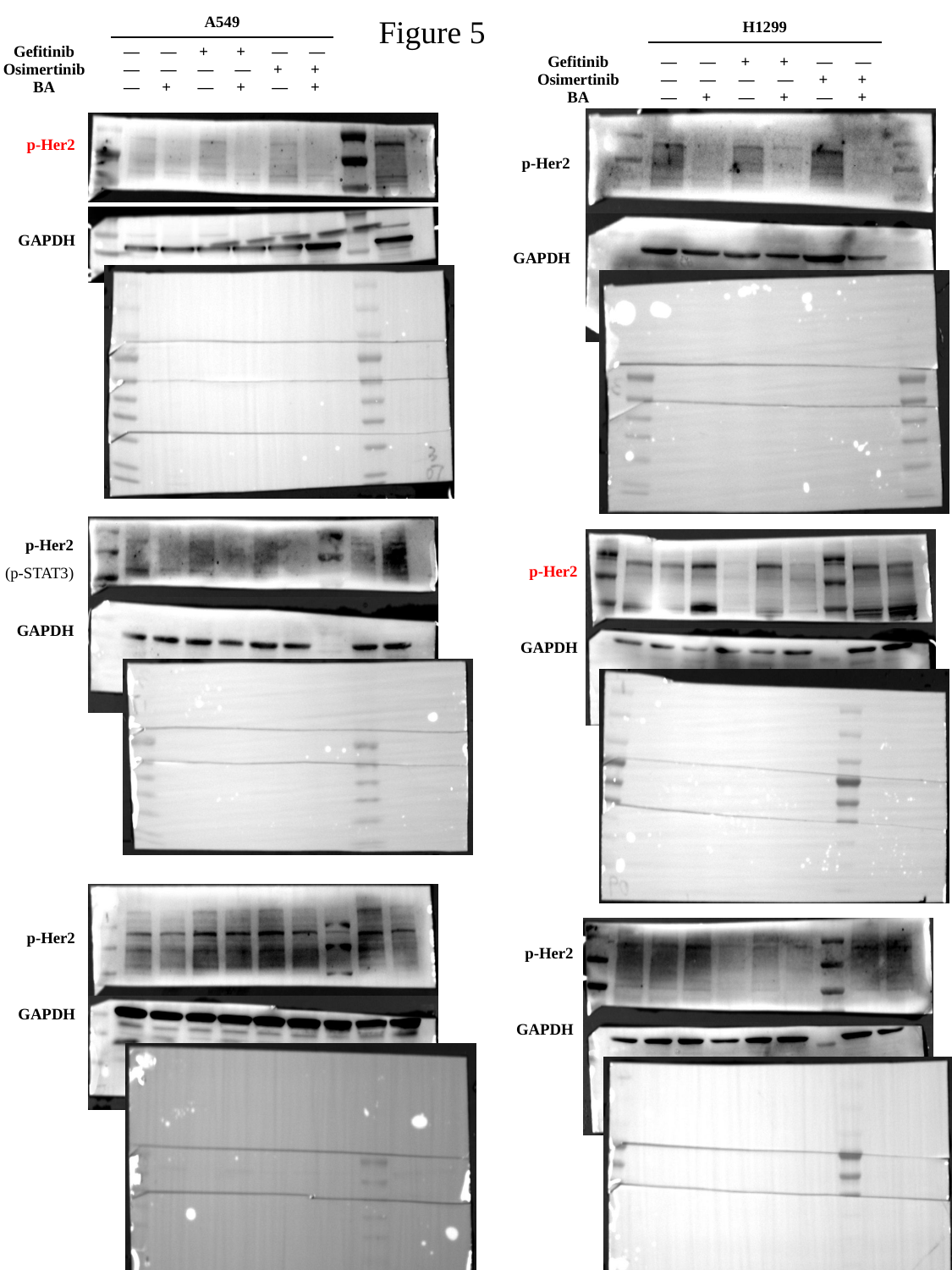

| | H1299 | | | | | |
| --- | --- | --- | --- | --- | --- | --- |
| Gefitinib Osimertinib BA | ——— | —— + | + —— | + — + | — + — | — + + |
| | A549 | | | | | |
| --- | --- | --- | --- | --- | --- | --- |
| Gefitinib Osimertinib BA | ——— | —— + | + —— | + — + | — + — | — + + |
Figure 5
p-Her2
GAPDH
p-Her2
GAPDH
p-Her2
GAPDH
p-Her2
(p-STAT3)
GAPDH
p-Her2
GAPDH
p-Her2
GAPDH

## Slide 35
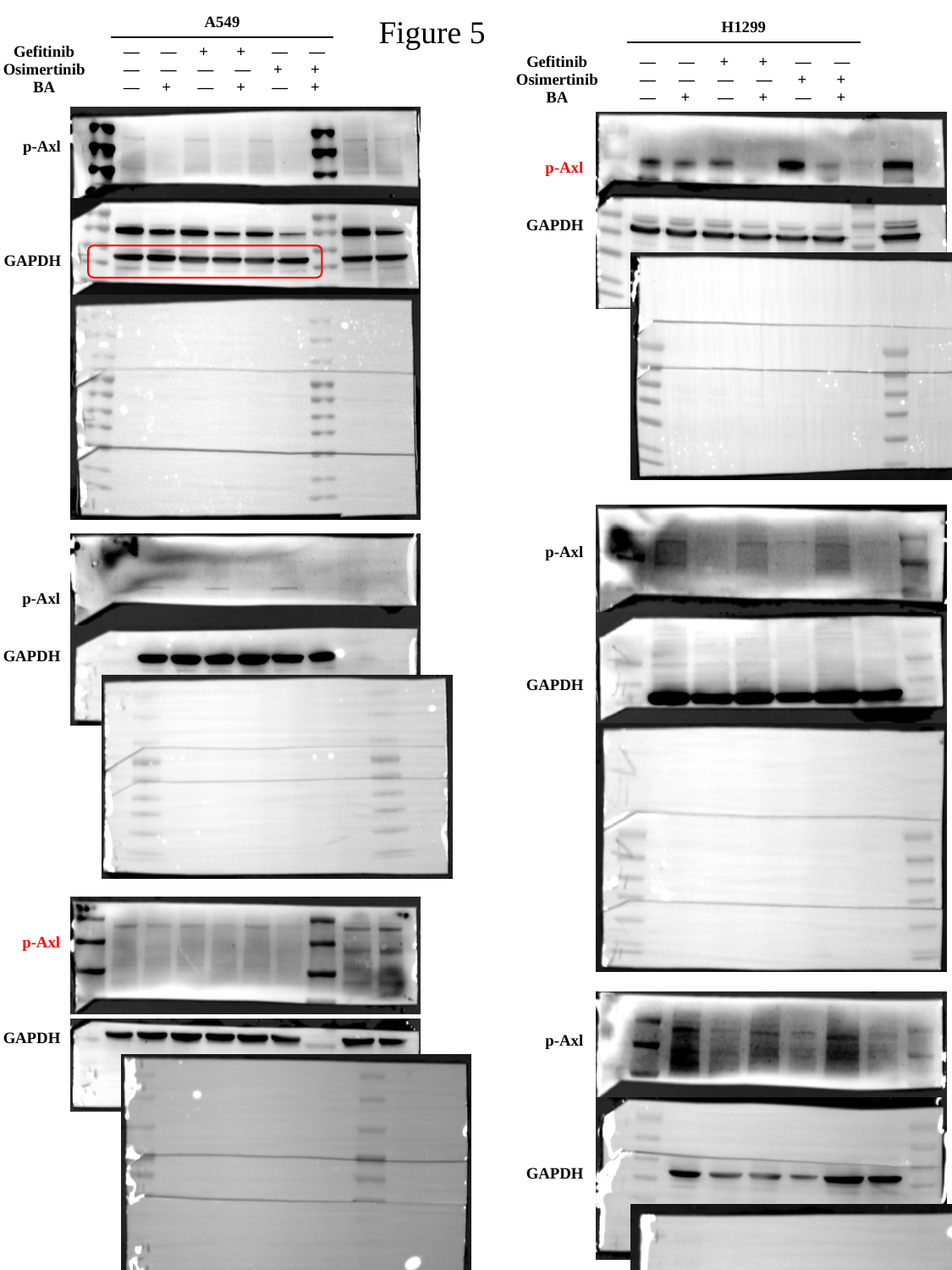

| | H1299 | | | | | |
| --- | --- | --- | --- | --- | --- | --- |
| Gefitinib Osimertinib BA | ——— | —— + | + —— | + — + | — + — | — + + |
| | A549 | | | | | |
| --- | --- | --- | --- | --- | --- | --- |
| Gefitinib Osimertinib BA | ——— | —— + | + —— | + — + | — + — | — + + |
Figure 5
p-Axl
GAPDH
p-Axl
GAPDH
p-Axl
GAPDH
p-Axl
GAPDH
p-Axl
GAPDH
p-Axl
GAPDH

## Slide 36
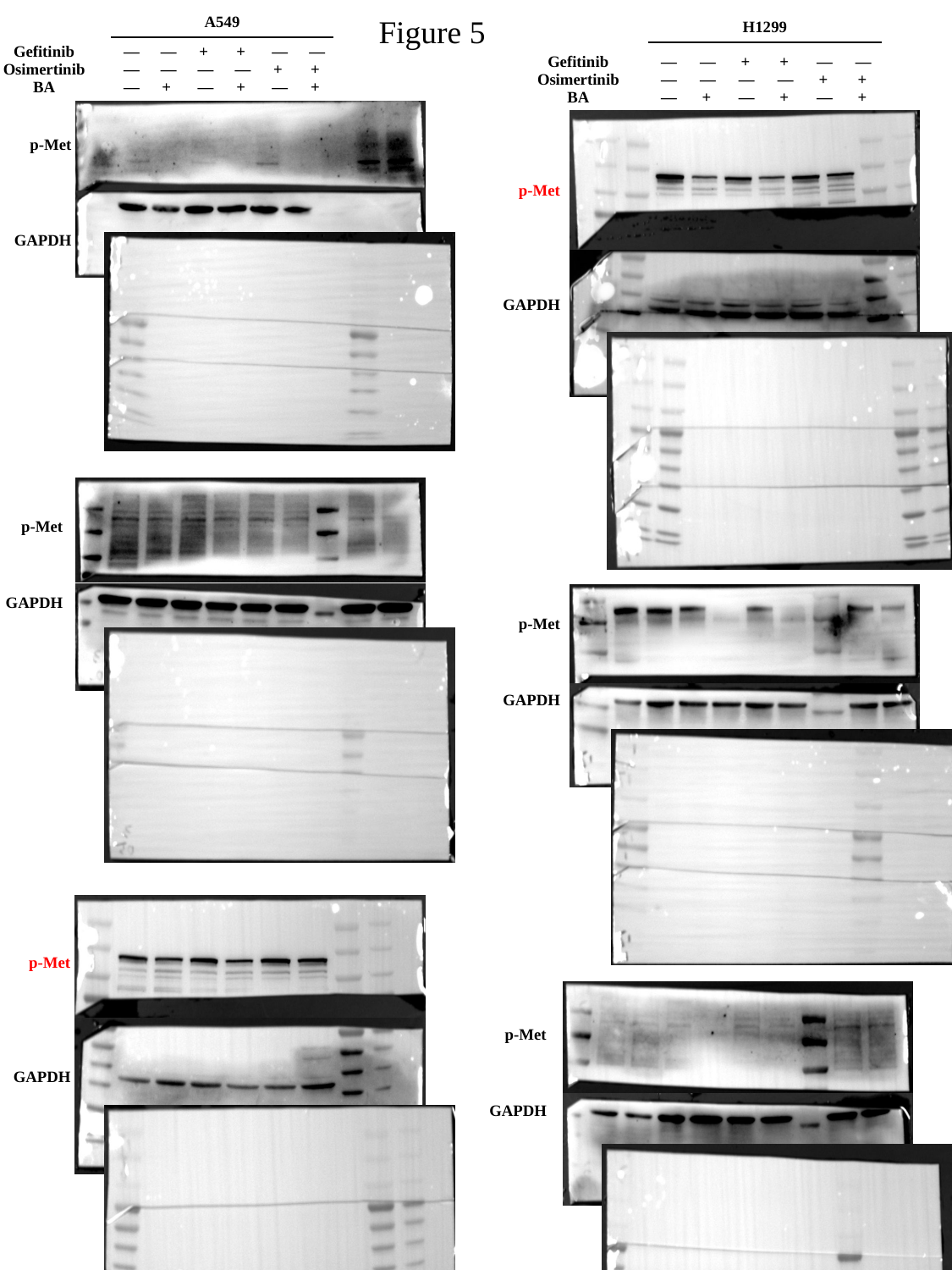

| | H1299 | | | | | |
| --- | --- | --- | --- | --- | --- | --- |
| Gefitinib Osimertinib BA | ——— | —— + | + —— | + — + | — + — | — + + |
| | A549 | | | | | |
| --- | --- | --- | --- | --- | --- | --- |
| Gefitinib Osimertinib BA | ——— | —— + | + —— | + — + | — + — | — + + |
Figure 5
p-Met
GAPDH
p-Met
GAPDH
p-Met
GAPDH
p-Met
GAPDH
p-Met
GAPDH
p-Met
GAPDH

## Slide 37
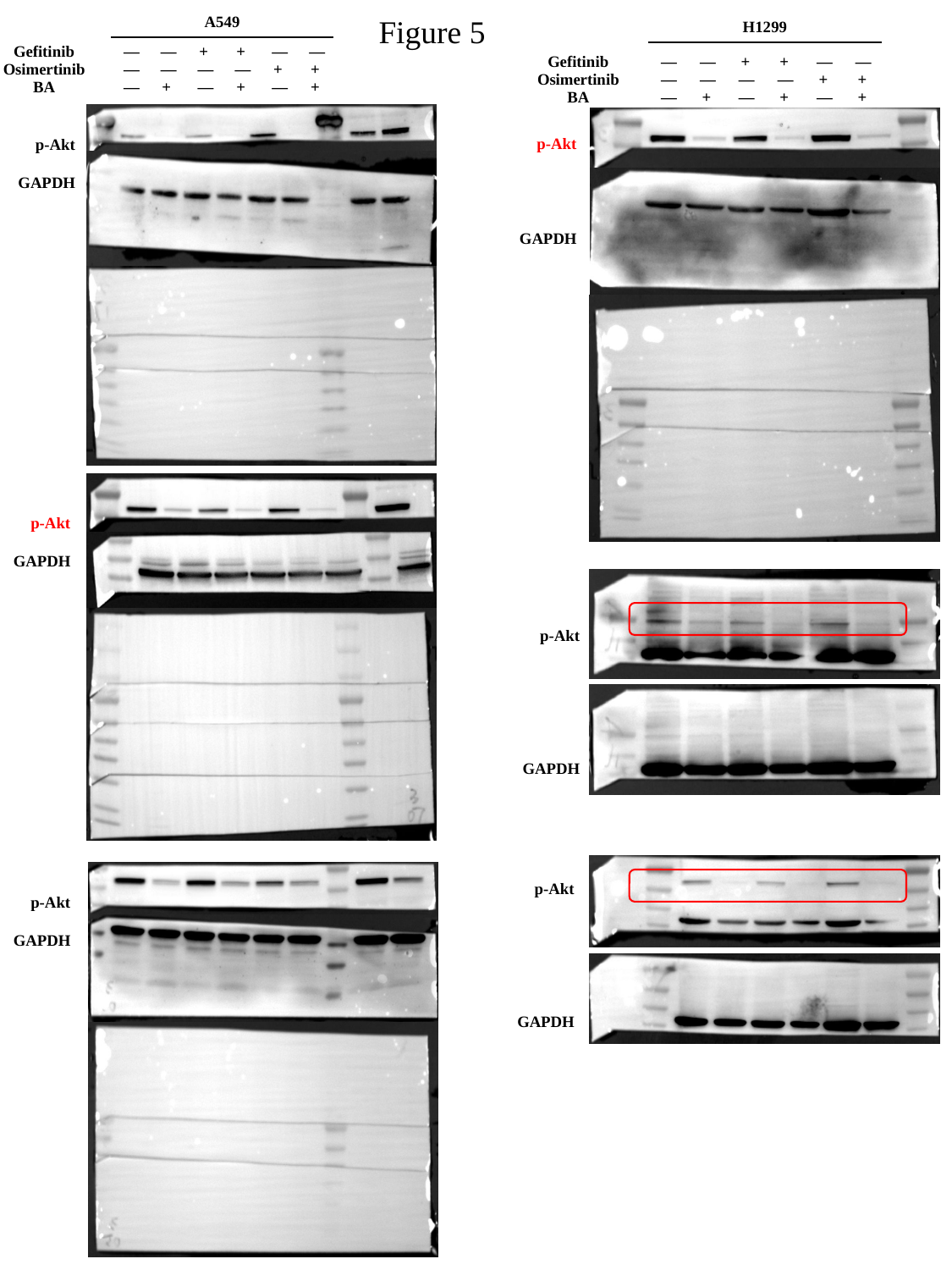

| | H1299 | | | | | |
| --- | --- | --- | --- | --- | --- | --- |
| Gefitinib Osimertinib BA | ——— | —— + | + —— | + — + | — + — | — + + |
| | A549 | | | | | |
| --- | --- | --- | --- | --- | --- | --- |
| Gefitinib Osimertinib BA | ——— | —— + | + —— | + — + | — + — | — + + |
Figure 5
p-Akt
GAPDH
p-Akt
GAPDH
p-Akt
GAPDH
p-Akt
GAPDH
p-Akt
GAPDH
p-Akt
GAPDH

## Slide 38
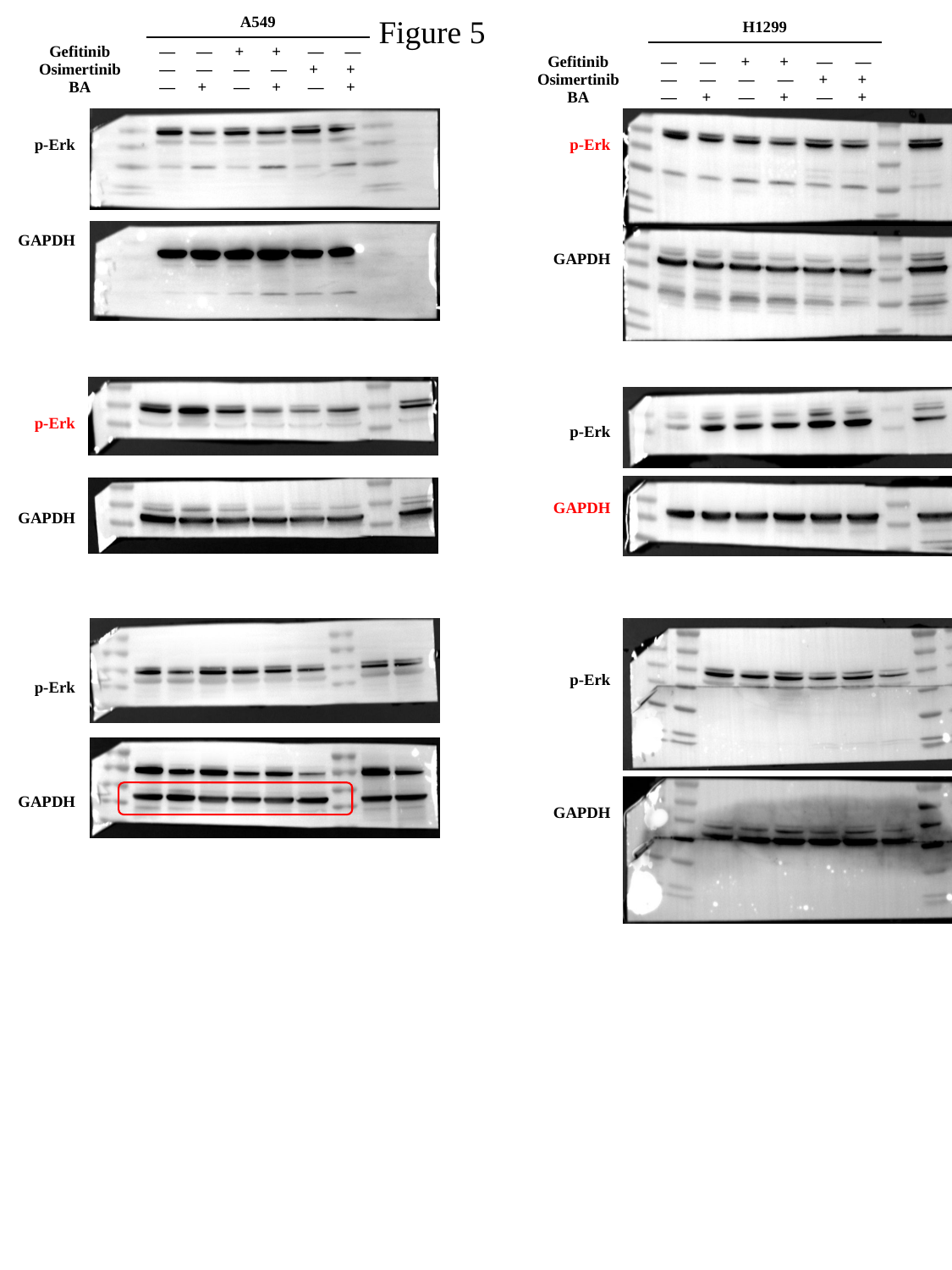

| | H1299 | | | | | |
| --- | --- | --- | --- | --- | --- | --- |
| Gefitinib Osimertinib BA | ——— | —— + | + —— | + — + | — + — | — + + |
| | A549 | | | | | |
| --- | --- | --- | --- | --- | --- | --- |
| Gefitinib Osimertinib BA | ——— | —— + | + —— | + — + | — + — | — + + |
Figure 5
p-Erk
GAPDH
p-Erk
GAPDH
p-Erk
GAPDH
p-Erk
GAPDH
p-Erk
GAPDH
p-Erk
GAPDH

## Slide 39
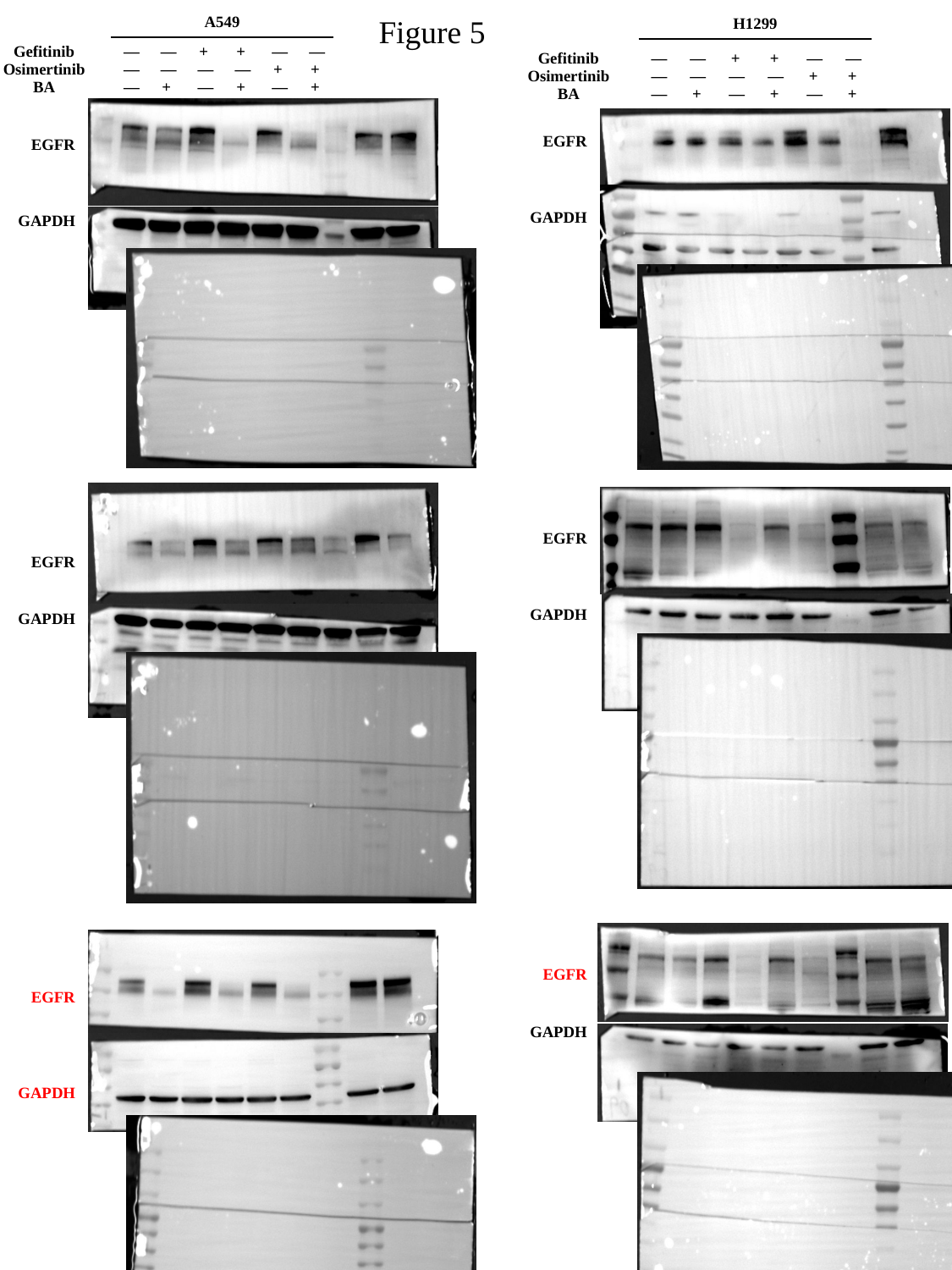

| | A549 | | | | | |
| --- | --- | --- | --- | --- | --- | --- |
| Gefitinib Osimertinib BA | ——— | —— + | + —— | + — + | — + — | — + + |
| | H1299 | | | | | |
| --- | --- | --- | --- | --- | --- | --- |
| Gefitinib Osimertinib BA | ——— | —— + | + —— | + — + | — + — | — + + |
Figure 5
EGFR
GAPDH
EGFR
GAPDH
EGFR
GAPDH
EGFR
GAPDH
EGFR
GAPDH
EGFR
GAPDH

## Slide 40
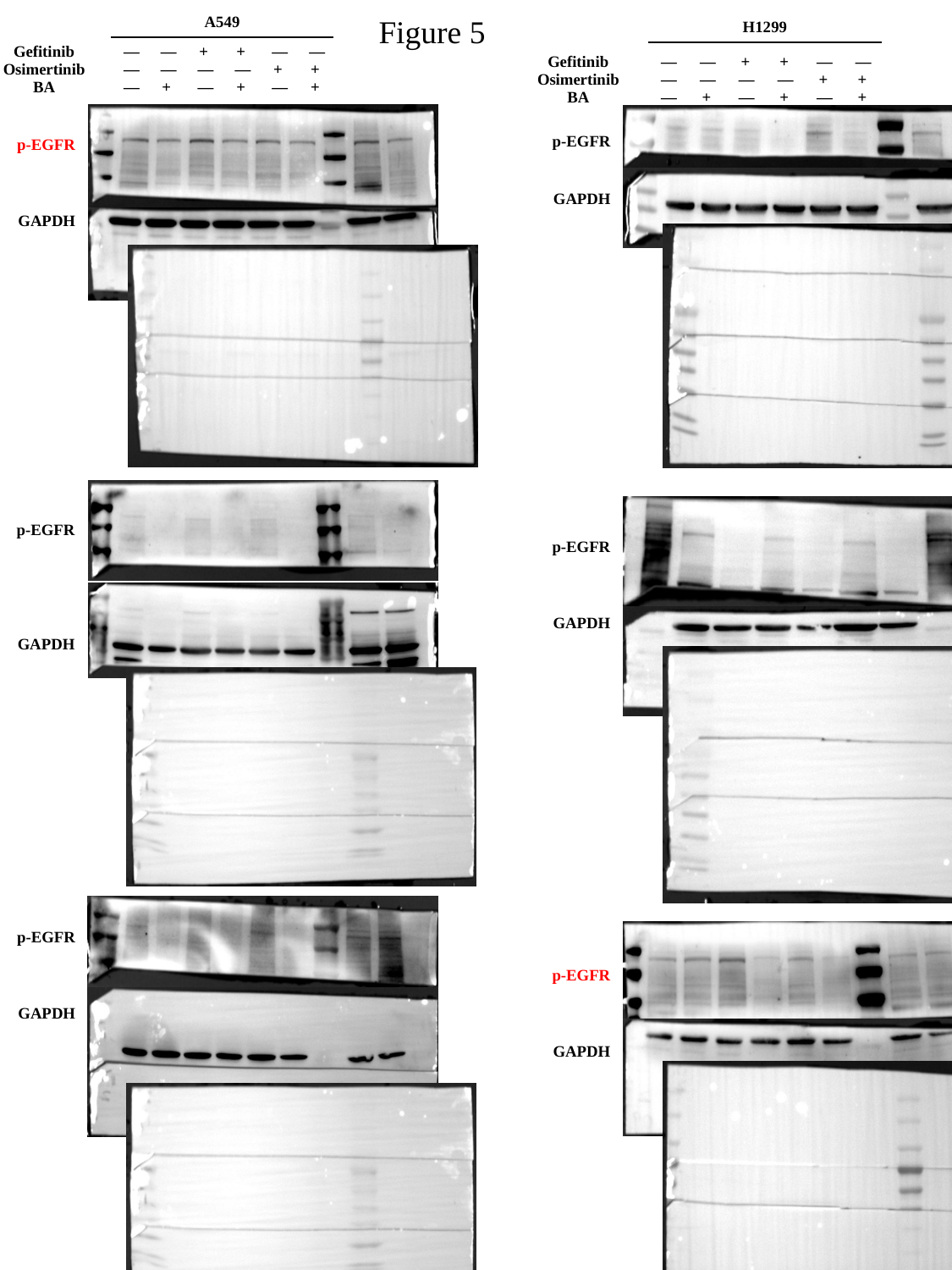

| | H1299 | | | | | |
| --- | --- | --- | --- | --- | --- | --- |
| Gefitinib Osimertinib BA | ——— | —— + | + —— | + — + | — + — | — + + |
| | A549 | | | | | |
| --- | --- | --- | --- | --- | --- | --- |
| Gefitinib Osimertinib BA | ——— | —— + | + —— | + — + | — + — | — + + |
Figure 5
p-EGFR
GAPDH
p-EGFR
GAPDH
p-EGFR
GAPDH
p-EGFR
GAPDH
p-EGFR
GAPDH
p-EGFR
GAPDH

## Slide 41
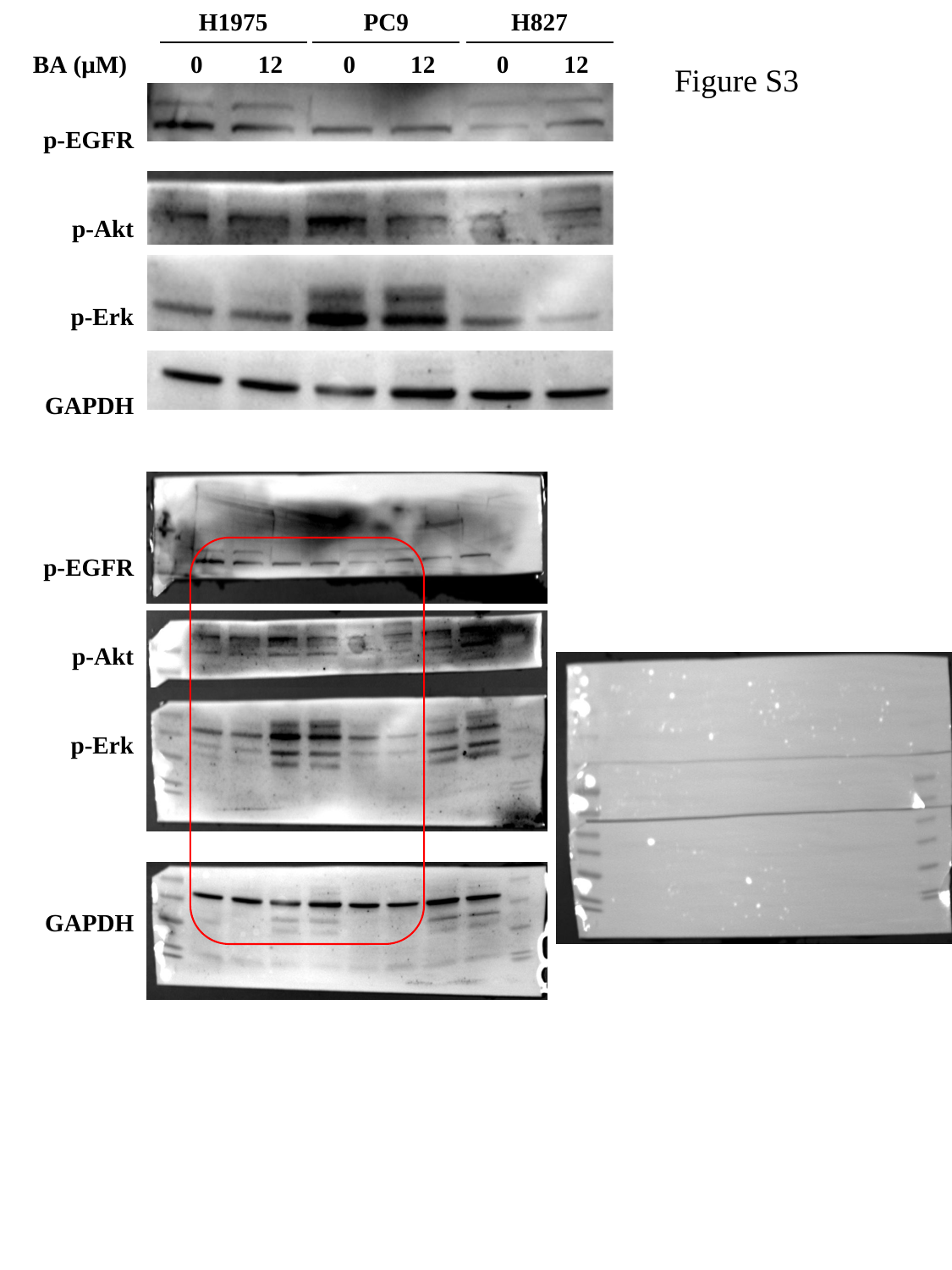

| | H1975 | |
| --- | --- | --- |
| BA (μM) | 0 | 12 |
| PC9 | |
| --- | --- |
| 0 | 12 |
| H827 | |
| --- | --- |
| 0 | 12 |
Figure S3
p-EGFR
p-Akt
p-Erk
GAPDH
p-EGFR
p-Akt
p-Erk
GAPDH

## Slide 42
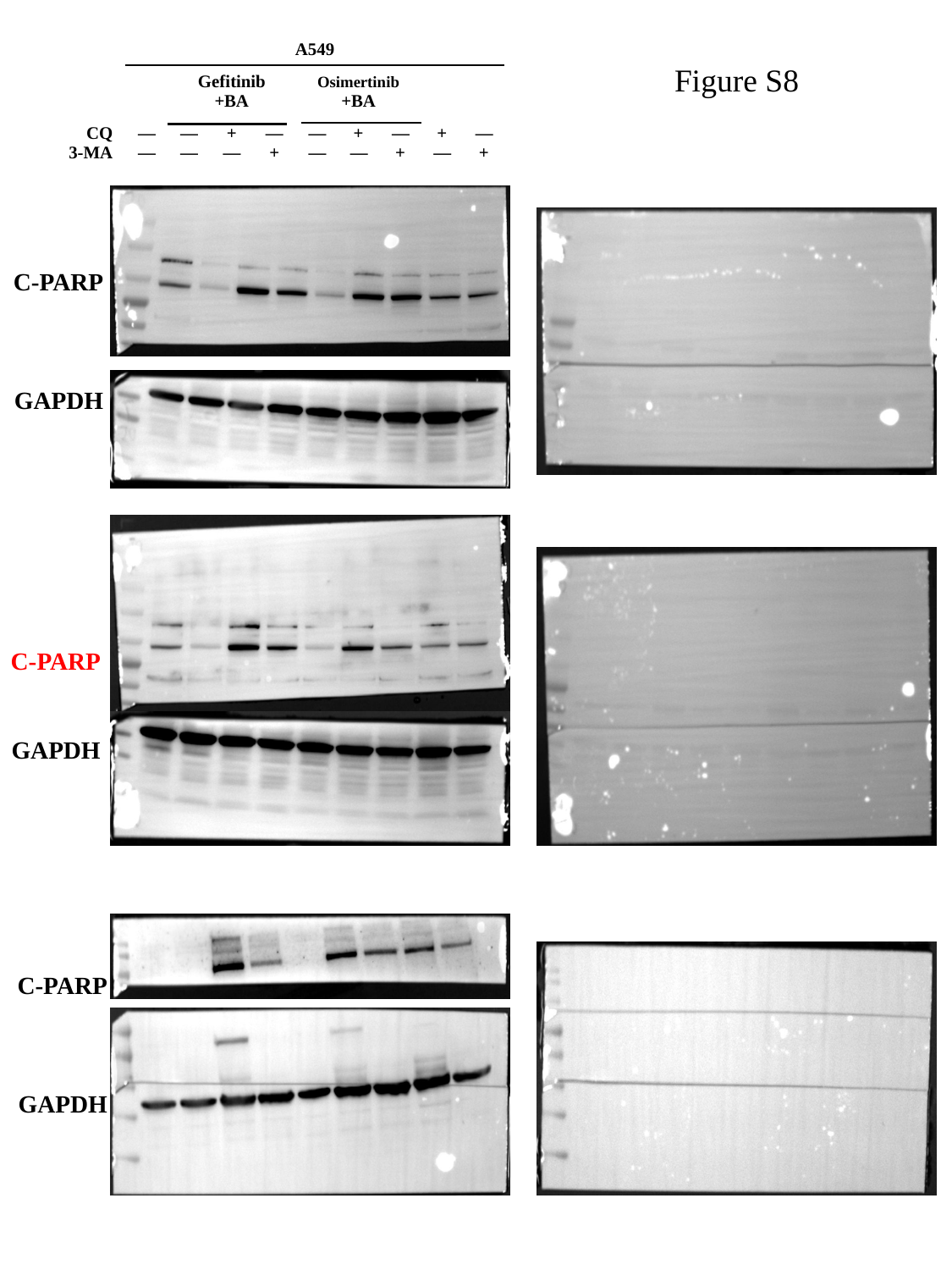

| | A549 | | | | | | | | |
| --- | --- | --- | --- | --- | --- | --- | --- | --- | --- |
| | | Gefitinib +BA | | | Osimertinib +BA | | | | |
| CQ 3-MA | —— | —— | + — | — + | —— | + — | — + | + — | — + |
Figure S8
C-PARP
GAPDH
C-PARP
GAPDH
C-PARP
GAPDH

## Slide 43
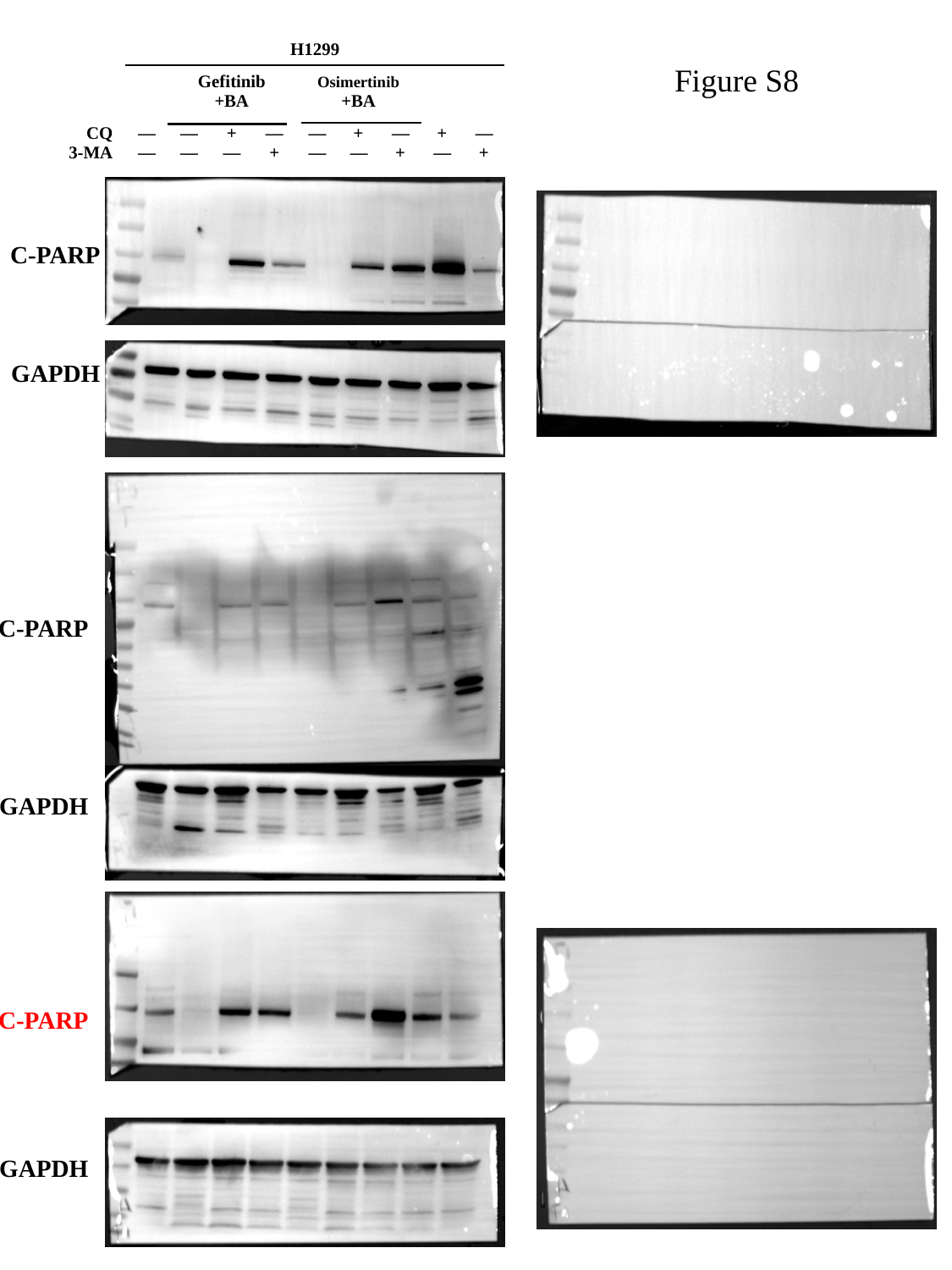

| | H1299 | | | | | | | | |
| --- | --- | --- | --- | --- | --- | --- | --- | --- | --- |
| | | Gefitinib +BA | | | Osimertinib +BA | | | | |
| CQ 3-MA | —— | —— | + — | — + | —— | + — | — + | + — | — + |
Figure S8
C-PARP
GAPDH
C-PARP
GAPDH
C-PARP
GAPDH
