# Supplementary material for: Combination of betulinic acid and EGFR-TKIs exerts synergistic anti-tumor effects against wild-type EGFR NSCLC by inducing autophagy-related cell death via EGFR signaling pathway
Source: Respir Res. 2024 May 20;25:215. doi: 10.1186/s12931-024-02844-9 (PMC11103851; doi:10.1186/s12931-024-02844-9)
Supplement: Supplementary file 2 — Supplementary Material 2. [file 12931_2024_2844_MOESM2_ESM.docx]

**Supplementary Material**


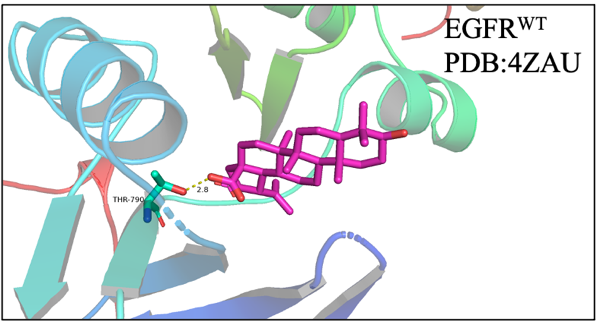


**Figure S1** The binding mode of BA docked into EGFR^WT^

^
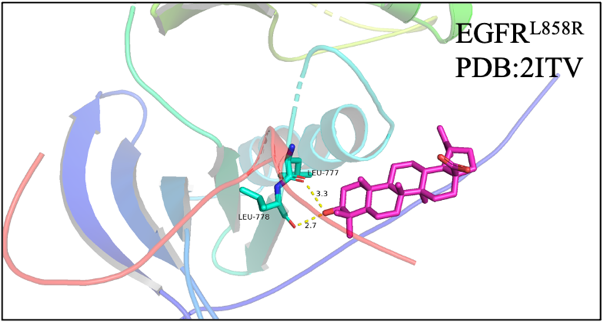
^

**Figure S2** The binding mode of BA docked into EGFRL^858R^

**
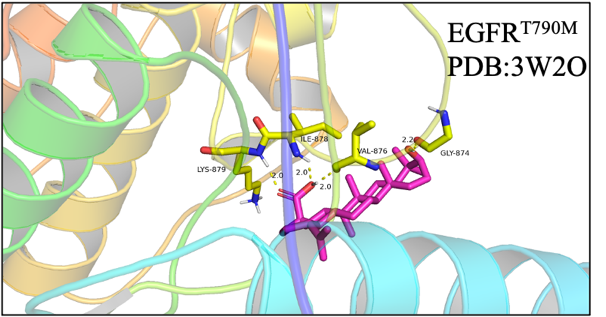
**

**Figure S3** The binding mode of BA docked into EGFR^T790M^

**
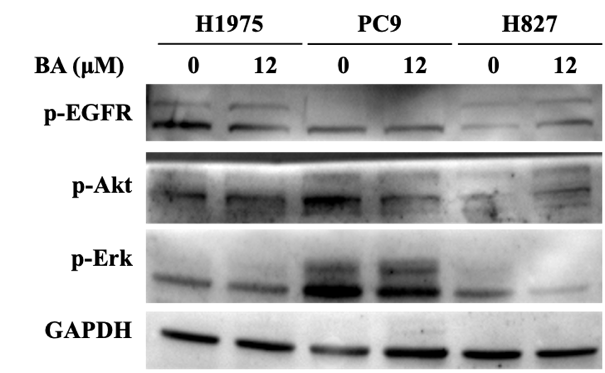
**

**Figure S4** The protein expression levels of p-EGFR and its downstream signaling with BA treatment were evaluated by western blotting.

**
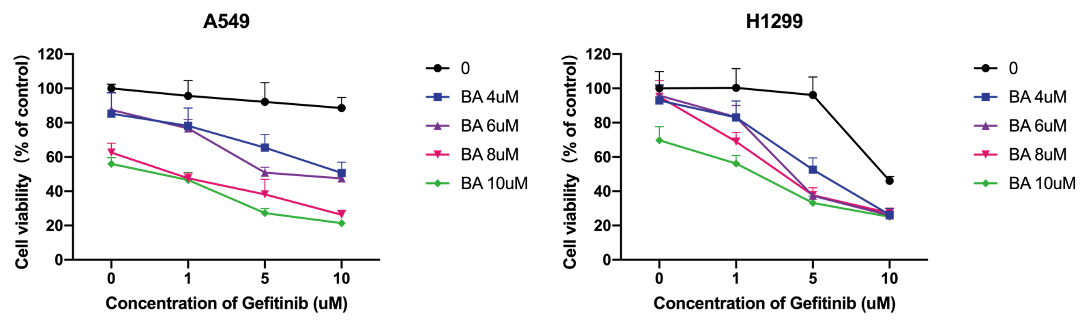
**

**Figure S5** The indicated cell lines were treated with varied concentrations of gefitinib alone, BA alone and their respective combinations for 48h, and cell viability was measured by CCK8 assay.

**
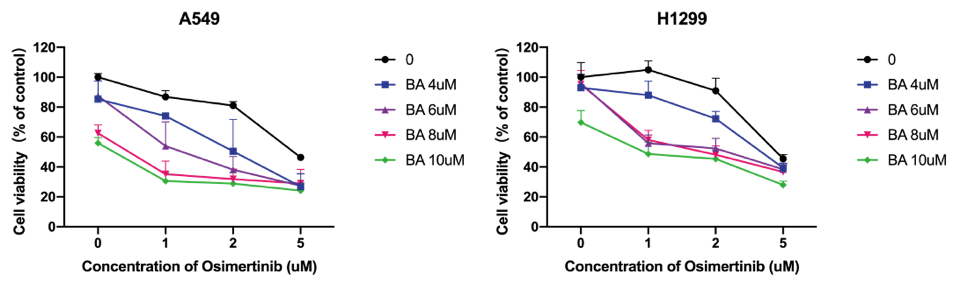
**

**Figure S6** The indicated cell lines were treated with varied concentrations of osimertinib alone, BA alone and their respective combinations for 48h, and cell viability was measured by CCK8 assay.


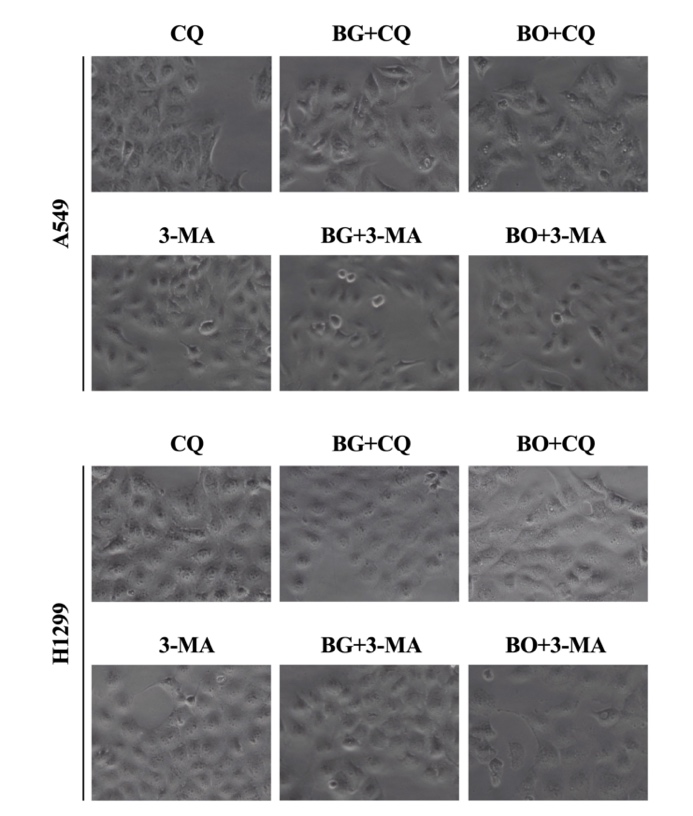


**Figure S7** Cell morphology of A549 and H1299 cells after treated with the indicated supplements for 48h.

**
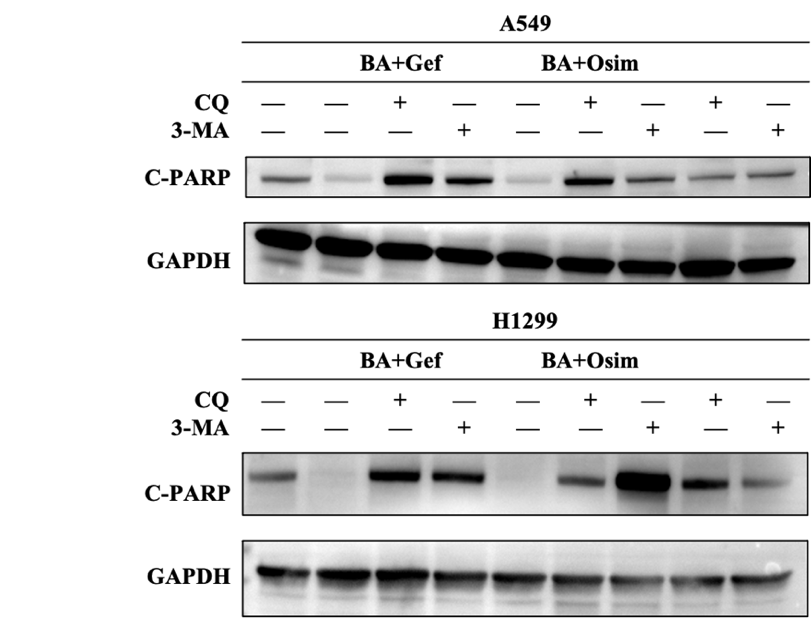
**

**Figure S8** A549 and H1299 cells were treated with the indicated supplements for 48h in the absence or presence of chloroquine (10 *μ*M) or 3-MA (5 mM). C-PARP protein was detected by immunoblotting. GAPDH was used as internal control.


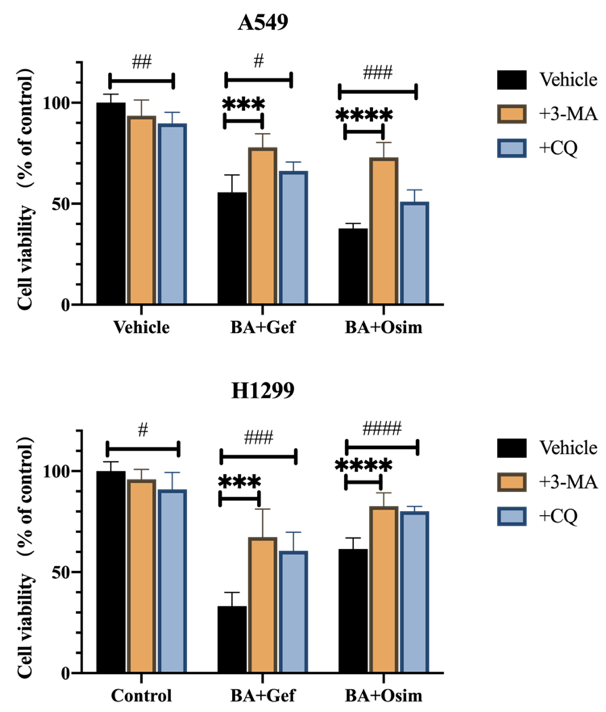


**Figure S9** A549 and H1299 cells were treated with the indicated supplements for 48h in the absence or presence of chloroquine (10 *μ*M) or 3-MA (5 mM). Cell viability was assessed by CCK8 assay (*or # *p*<0.05, ** or ## *p*<0.01, *** or ### *p*<0.001, **** or #### *p*<0.0001).


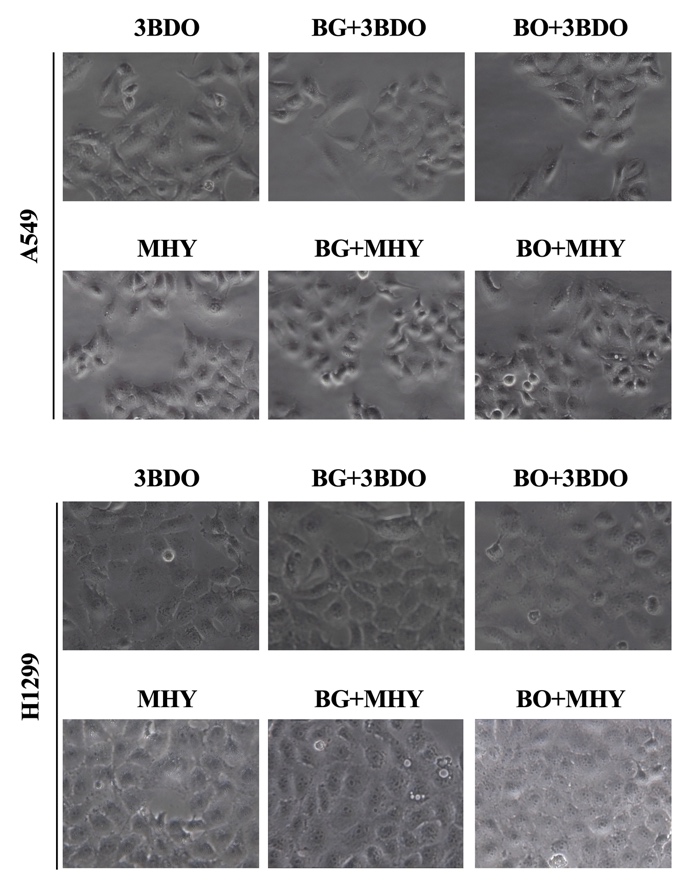


**Figure S10** Cell morphology of A549 and H1299 cells after treated with the indicated supplements for 48h.


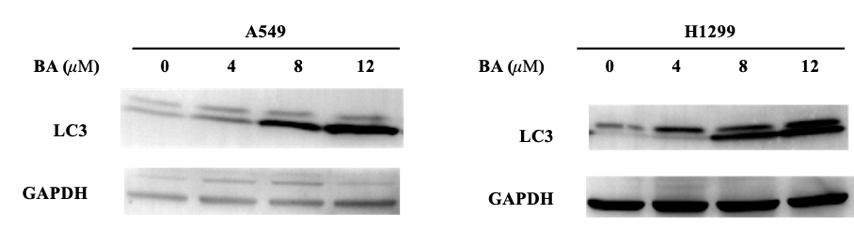


**Figure S11** The protein expression levels of LC3 with BA treatment were evaluated by western blotting.
